# SNAIL1-mediated downregulation of FOXA proteins facilitates the inactivation of transcriptional enhancer elements at key epithelial genes in colorectal cancer cells

**DOI:** 10.1371/journal.pgen.1007109

**Published:** 2017-11-20

**Authors:** Sabine Jägle, Hauke Busch, Vivien Freihen, Sven Beyes, Monika Schrempp, Melanie Boerries, Andreas Hecht

**Affiliations:** 1 Institute of Molecular Medicine and Cell Research, Faculty of Medicine, University of Freiburg, Freiburg, Germany; 2 German Cancer Consortium (DKTK), Freiburg, Germany; 3 German Cancer Research Center (DKFZ), Heidelberg, Germany; 4 Institute of Experimental Dermatology and Institute of Cardiogenetics, University of Lübeck, Lübeck, Germany; 5 Faculty of Biology, University of Freiburg, Freiburg, Germany; 6 BIOSS Centre for Biological Signalling Studies, University of Freiburg, Freiburg, Germany; Institute of Technology, UNITED STATES

## Abstract

Phenotypic conversion of tumor cells through epithelial-mesenchymal transition (EMT) requires massive gene expression changes. How these are brought about is not clear. Here we examined the impact of the EMT master regulator SNAIL1 on the FOXA family of transcription factors which are distinguished by their particular competence to induce chromatin reorganization for the activation of transcriptional enhancer elements. We show that the expression of *SNAIL1* and *FOXA* genes is anticorrelated in transcriptomes of colorectal tumors and cell lines. In cellular EMT models, ectopically expressed Snail1 directly represses FOXA1 and triggers downregulation of all FOXA family members, suggesting that loss of FOXA expression promotes EMT. Indeed, cells with CRISPR/Cas9-induced FOXA-deficiency acquire mesenchymal characteristics. Furthermore, ChIP-seq data analysis of FOXA chromosomal distribution in relation to chromatin structural features which characterize distinct states of transcriptional activity, revealed preferential localization of FOXA factors to transcriptional enhancers at signature genes that distinguish epithelial from mesenchymal colon tumors. To validate the significance of this association, we investigated the impact of FOXA factors on structure and function of enhancers at the *CDH1*, *CDX2* and *EPHB3* genes. FOXA-deficiency and expression of dominant negative FOXA2 led to chromatin condensation at these enhancer elements. Site-directed mutagenesis of FOXA binding sites in reporter gene constructs and by genome-editing *in situ* impaired enhancer activity and completely abolished the active chromatin state of the *EPHB3* enhancer. Conversely, expression of FOXA factors in cells with inactive *CDX2* and *EPHB3* enhancers led to chromatin opening and *de novo* deposition of the H3K4me1 and H3K27ac marks. These findings establish the pioneer function of FOXA factors at enhancer regions of epithelial genes and demonstrate their essential role in maintaining enhancer structure and function. Thus, by repressing FOXA family members, SNAIL1 targets transcription factors at strategically important positions in gene-regulatory hierarchies, which may facilitate transcriptional reprogramming during EMT.

## Author summary

Cancer patient mortality is overwhelmingly due to distant organ metastases. Epithelial-mesenchymal transition is a process thought to facilitate local invasion and dissemination of cancer cells, thereby promoting metastasis. The conversion of epithelial cells into mesenchymal, fibroblast-like cells requires profound gene expression changes. A few transcription factors like SNAIL1 can initiate these changes, but are unlikely to be solely responsible for all of them. In our study we asked, whether destabilization of epithelial gene expression programs could involve FOXA transcription factors. FOXA factors represent a special subgroup of regulatory proteins, so-called pioneer factors, with unique roles in the activation of transcriptional enhancers which are key regulatory DNA elements that orchestrate spatio-temporal gene expression. In a model of colorectal cancer we found that SNAIL1 represses FOXA factors, and demonstrate that FOXA factors are associated with enhancer elements at epithelial signature genes. Indeed, FOXA factors are sufficient to initiate enhancer activation and necessary to maintain their activity. Our findings indicate that SNAIL1 induces pervasive repression of epithelial genes through a hierarchical scheme of alterations in transcription factor expression which may be applicable to other instances of cell fate changes and transcriptional reprogramming.

## Introduction

The formation of distant organ metastases and acquired therapy resistance represent major challenges for the successful treatment of cancer. The process of epithelial-mesenchymal transition (EMT) is widely believed to promote initial steps in the invasion-metastasis cascade [1]. EMT was also linked to reduced cancer cell sensitivity against chemo- and radiotherapy [2–4]. Phenotypic changes of tumor cells that undergo EMT include loss of apical-basal cell polarity, alterations in cell-cell and cell-matrix attachment, increased motility, and enhanced invasiveness [1]. The observed adaptations in cellular phenotype are caused by extensive transcriptional reprogramming, during which the expression of epithelial and mesenchymal genes is down- and upregulated, respectively [1,5]. EMT and the accompanying gene expression changes are triggered by members of the SNAIL, ZEB and TWIST families of transcription factors which largely act as transcriptional repressors but can also activate gene expression [1,5]. However, it is unlikely that these transcriptional regulators are directly involved in the regulation of all genes whose expression is affected during EMT.

Transcriptional enhancers are *cis*-acting DNA elements with pivotal functions in gene regulation [6]. Aside from their decisive importance in physiological control of gene expression, it is increasingly recognized that landscapes of active enhancers change dramatically during tumorigenesis [7–10]. The defining hallmark of enhancers is their ability to stimulate transcription from a linked promoter in a manner that is largely independent of orientation and distance. In agreement with their central roles in orchestrating differential gene expression, enhancers function in a highly context-dependent manner and can adopt different functional states that are reflected by characteristic chromatin structural features [6]: Inactive and latent enhancers are not bound by transcription factors, and their DNA is packaged into nucleosomes which are devoid of distinctive histone modifications. In contrast, chromosomal DNA of poised and active enhancers is bound by transcription factors and nucleosome-free. Besides, when compared to promoter regions, nucleosomes flanking poised and active enhancers display elevated levels of mono- and dimethylated lysine 4 in histone H3 (H3K4me1/2). Active enhancers can be distinguished from poised enhancers by their association with the acetyltransferase p300 and by the appearance of acetylated lysine 27 in H3 (H3K27ac) in adjacent chromatin. The dynamic changes in enhancer structure and function depend on the presence or absence of multifactorial assemblies of transcriptional regulators and their co-factors which are recruited to enhancer regions through sequence-specific DNA-binding. Pioneer factors form a particular subgroup of enhancer-binding proteins which are distinct from other transcription factors due to their ability to occupy DNA-binding sites even when these are presented in a nucleosomal configuration [11]. Accordingly, pioneer factors are uniquely required for the initial steps of chromatin opening that antecede further events in enhancer activation.

FOXA1, FOXA2, and FOXA3 are closely related DNA-binding proteins which are known to function as pioneer factors [11,12]. They form a subgroup of the evolutionary conserved family of forkhead domain transcriptional regulators and play essential roles in the development of endoderm and endoderm-derived organs [12]. In the adult, FOXA genes are widely expressed in different tissues including the mammary gland, lung, pancreas, liver, intestine, and the prostate where they control cellular differentiation and organ function [12–14]. FOXA factors are also implicated in the tumorigenesis of several organs. For example, FOXA1 and FOXA2 cooperate with nuclear hormone receptors in endocrine-driven tumors of the breast, prostate and liver [15,16]. In lung, breast and pancreatic cancer, FOXA factors were shown to be tightly linked to an epithelial cell state and to suppress the adoption of a mesenchymal phenotype [17–20]. Fittingly, in mouse prostate tumor cells, FOXA1 and SNAIL2/SLUG reciprocally repress each other [21], and FOXA1 was reported to stimulate promoter activity of the epithelial marker gene *CDH1* (coding for E-CADHERIN) in human breast cancer cells [22].

During EMT, a pervasive repression of epithelial genes takes place. A possible mechanistic explanation for this could be that EMT-inducing transcriptional regulators target transcription factors at strategically important positions in gene-regulatory hierarchies to inactivate enhancer elements at a broad range of epithelial genes. Here we tested this hypothesis and show that SNAIL1 downregulates all three FOXA family members in a colorectal cancer (CRC) model of EMT. The loss of FOXA factors in turn impairs the structure and function of enhancers at the tumor suppressor genes *CDH1*, *EPHB3* and *CDX2* which are integral to the differentiation and tissue integrity of the intestinal epithelium [23–26]. It appears that the EMT process engages a hierarchical scheme of changes in transcription factor activity to achieve massive transcriptional reprogramming.

## Results

### Expression of the EMT inducer Snail1 reduces FOXA expression

**Fig 1:**
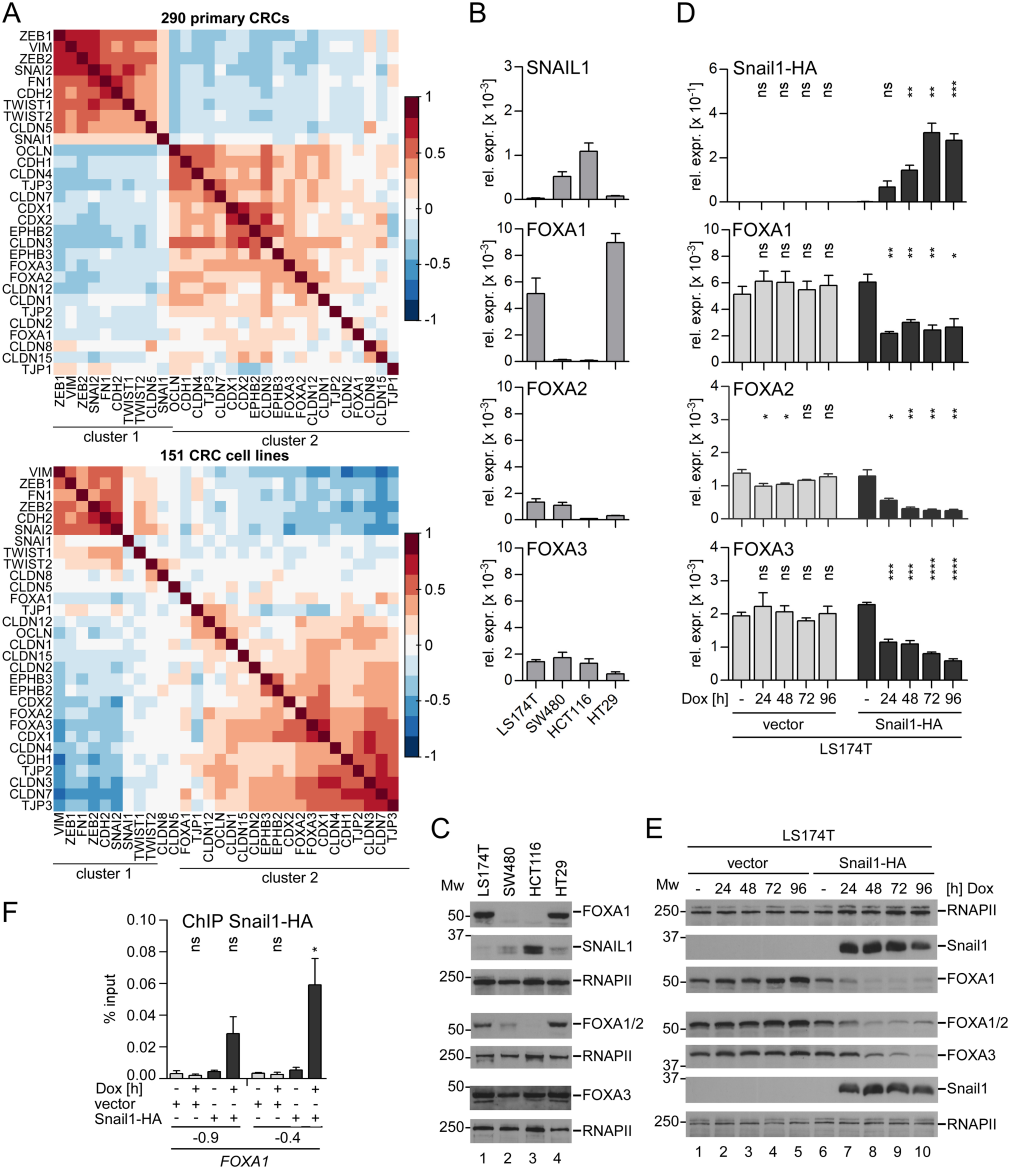
SNAIL1 is anticorrelated and interferes with FOXA expression. (A) Pairwise correlation analyses of mRNA expression data from 290 primary CRCs (GSE14333) (upper panel) and from 151 CRC cell lines (GSE59857) (lower panel). Correlation coefficients are indicated by the different colors and the color scale shown. (B) qRT-PCR analyses to assess *SNAIL1*, *FOXA1*, *FOXA2*, and *FOXA3* relative expression (rel. expr.) levels in a panel of CRC cell lines. Data are shown as mean and standard error of the mean (SEM); n = 3. (C) Western Blot to analyze SNAIL1, FOXA1, FOXA1/2 and FOXA3 protein expression in a panel of CRC cell lines. FOXA2 is detected simultaneously with FOXA1 due to crossreactivity of the antibody used (S2 Fig). M_W_ = molecular weight in kDa. RNA polymerase II (RNAPII) immunodetection served as loading control. For every membrane used for protein detection an individual loading control is shown. (D) qRT-PCR analyses to assess *Snail1*, *FOXA1*, *FOXA2*, and *FOXA3* relative expression (rel. expr.) levels in LS174T cells stably transduced with Dox-inducible retroviral control or Snail1-HA expression vectors. Shown is the mean and SEM; n = 3. (E) Western Blot to analyze Snail1-HA, FOXA1, FOXA1/2, and FOXA3 protein levels upon Dox-induced Snail1-HA expression in LS174T cells. M_W_ = molecular weight in kDa. To monitor equal protein loading RNA polymerase II (RNAPII) was detected. (F) ChIP analysis to test for Snail1-HA occupancy at the *FOXA1* promoter in LS174T cells. Data were calculated as percent input. Shown are the mean and SEM; n = 3.

Expression of EMT inducers leads to profound changes in cell morphology and is characterized by massive changes in gene expression. In different tumor entities expression of FOXA proteins has been associated with expression of epithelial genes [17,18,20]. To find out if this correlation can also be observed in CRC, we performed pairwise correlation analyses using gene expression data from 290 primary CRCs (GSE14333). Indeed, *FOXA1*, *FOXA2*, and *FOXA3* clustered together with epithelial genes that are involved in the formation of adherens junctions (*CDH1*) and tight junctions (*OCLN*, *TJP1-3*, *CLDN 1*, *2*, *3*, *4*, *7*, *8*, *12*, *15*) (Fig 1A, upper panel, cluster 2). Moreover, *FOXA1*, *FOXA2*, and *FOXA3* were also grouped together with the tumor/invasion suppressor genes *EPHB2* and *EPHB3*, and with the intestine-specific differentiation genes *CDX1* and *CDX2* (Fig 1A, upper panel, cluster 2). In contrast, mesenchymal marker genes (*VIM*, *FN1*, *CDH2*; coding for N-CADHERIN) and EMT inducers (*SNAI1*, *SNAI2*, *ZEB1*, *ZEB2*, *TWIST1*, *TWIST2*) formed a separate cluster of co-expressed genes (Fig 1A, upper panel, cluster 1). Members of the two clusters typically showed opposing expression levels in CRC transcriptomes (S1 Fig, panel A). The association of FOXA genes with epithelial genes and their separation from mesenchymal genes observed in human colorectal tumors was also evident when we analyzed transcriptome data from 151 CRC cell lines (GSE59857) (Fig 1A, lower panel; S1 Fig, panel B). We also found that comparably higher expression levels of the mesenchymal signature genes in cluster 1 are correlated with metastasized CRC (Duke’s stage D), while expression levels of epithelial genes in cluster 2 are significantly higher in transcriptomes from Duke’s stage A tumors (S1 Table). Thus, the inversely correlated up- and downregulation of mesenchymal and epithelial genes including the FOXA family, respectively, correlates with malignant CRC progression.

We also investigated expression of *SNAIL1* and FOXA factors in a panel of four CRC cell lines by quantitative reverse transcription PCR (qRT-PCR) and Western Blotting. In this model, the expression of *FOXA1* turned out to be mutually exclusive with that of *SNAIL1*, while elevated *SNAIL1* expression was not paralleled by lower levels of *FOXA2* and *FOXA3* (Fig 1B and 1C). These relationships were observed on RNA and protein levels except for *FOXA2*. The apparent discrepancy between FOXA2 transcript and protein profiles probably is due to considerable crossreactivity of the FOXA2 antibody with FOXA1 (S2 Fig). Accordingly, Western Blot signals in LS174T and HT29 cells represent a combination of FOXA1 and FOXA2 expression. Nonetheless, the data shown in Fig 1B and 1C provide experimental evidence for anticorrelated expression of *SNAIL1* and FOXA factors.

The negative correlation of FOXA proteins with the EMT inducers in human colorectal tumors and CRC cell lines suggested a possible regulation of FOXA factors by EMT inducers. To directly test if SNAIL1 can interfere with the expression of FOXA genes, we chose two different EMT model systems based on doxycycline- (Dox-) inducible expression of epitope-tagged murine Snail1-HA in the CRC cell lines LS174T and HT29 [27]. Upon Dox-mediated Snail1-HA induction, expression of *FOXA1* and *FOXA3* factors was reduced over time in both cell lines (Fig 1D and 1E and S3 Fig panels A, B). *FOXA2* expression was not changed in HT29 cells, which per se show low *FOXA2* levels. Yet, *FOXA2* RNA and protein levels dropped in LS174T cells (Fig 1D and 1E and S3 Fig panels A, B). Due to the downregulation of *FOXA1* upon Snail1-HA expression and their opposing expression in CRC cell lines, we wondered whether *FOXA1* might be directly repressed by Snail1-HA. Indeed, ChIP analyses showed an enrichment of Snail1-HA at two sites of the *FOXA1* promoter after 6h of Dox treatment in LS174T and HT29 cells (Fig 1F and S3 Fig panel C). To test whether this interaction constitutes a repressive event we performed reporter gene experiments with a *FOXA1* promoter construct in LS174T and HT29 cells (S4 Fig). In both cell lines activity of the wild-type FOXA1 promoter was significantly reduced by coexpression of Snail1-HA. This inhibition was abolished upon mutation of two E-box motifs representing potential SNAIL1 binding sites as indicated by the ChIP experiments.

**Fig 2:**
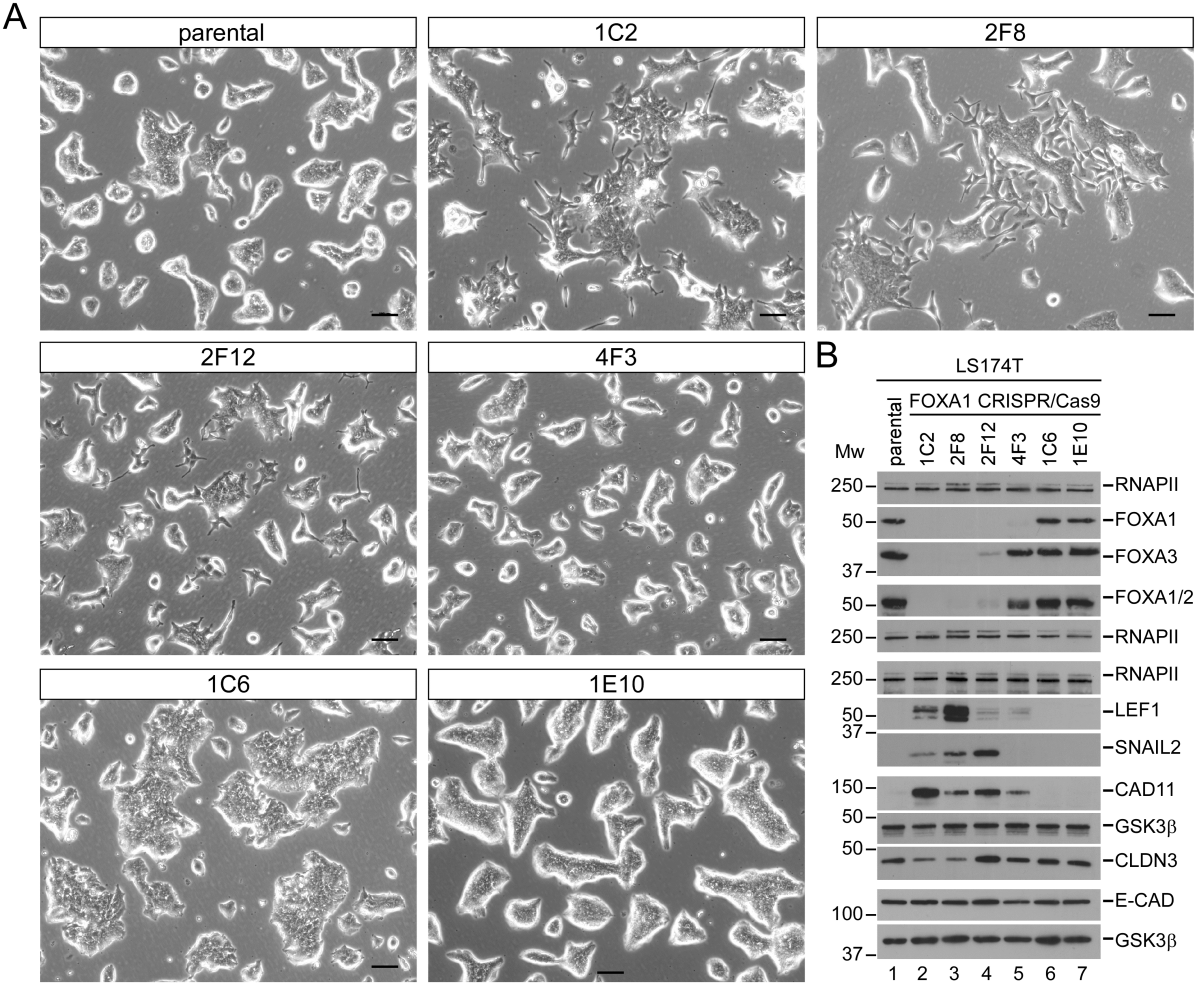
LS174T cell clones with absent/low FOXA expression show a mesenchymal morphology and upregulation of mesenchymal marker proteins. (A) Phase contrast images of parental LS174T cells and cell clones subjected to CRISPR/Cas9-mediated genome editing of the FOXA1 locus. Scale bar: 100 μm. (B) Western Blot analyses to assess expression of FOXA1, FOXA2, FOXA3, E-CADHERIN (E-CAD), CLAUDIN3 (CLDN3), CADHERIN11 (CAD11), LEF1 and SNAIL2/SLUG in parental LS174T cells and cell clones subjected to CRISPR/Cas9-mediated genome editing of the FOXA1 locus. RNA polymerase II (RNAPII) and GSK3β immunodetections served as loading controls. Analytes which were detected on separate membranes are grouped together including their corresponding loading controls. M_W_ = molecular weight in kDa.

In view of their anticorrelated expression and the downregulation of FOXA family members by SNAIL1 we sought to provide functional evidence that loss of FOXA proteins might promote EMT. To address this we used CRISPR/Cas9-mediated genome editing and single cell cloning, aiming to isolate derivatives of the epithelial CRC cell line LS174T with a knockout of the *FOXA1* gene. Among 56 cell clones analyzed we obtained 22 in which genome editing had not been effective. These cells morphologically resembled the parental LS174T cell line (Fig 2, e. g. clones 1C6, 1E10). However, we also found four FOXA1-deficient clones (Fig 2, clones 1C2, 2F8, 2F12, 4F3). Interestingly, these FOXA1^KO^ cell clones displayed heterogeneous morphologies, either looking like the parental LS174T cells (Fig 2A, clone 4F3), or presenting with a more mesenchymal appearance (Fig 2A, clones 1C2, 2F8, 2F12). Upon closer examination it turned out that clone 4F3 had lost the FOXA1 protein, but continued to express FOXA2 and FOXA3 (Fig 2B and S2 Table). In contrast, the mesenchymal-looking derivatives in addition to FOXA1 deficiency exhibited much reduced expression of FOXA2 and FOXA3 (Fig 2B and S2 Table, clone 2F12; FOXA1^KO^A2A3^lo^) or their complete absence (Fig 2B and S2 Table, clones 1C2, 2F8; FOXA1^KO^A2A3^neg^). The molecular basis for the concomitant reduction or loss of expression of FOXA2 and FOXA3 upon knockout of FOXA1 in these cell clones is not clear. Irrespective of this, the acquisition of a more mesenchymal morphology by FOXA1^KO^A2A3^lo/neg^ cells was accompanied by the upregulation of SNAIL2/SLUG, and LEF1, two transcription factors with EMT-inducing capacities [1,5], and of CADHERIN11 (CDH11, alternative name: OB-CADHERIN), a marker of mesenchymal cells [1,28] (Fig 2B, S5 Fig). CADHERIN11 expression was elevated in all four FOXA1^KO^ clones. Likewise, the increase in LEF1 levels was seen in all FOXA1^KO^ clones, albeit FOXA1^KO^A2A3^neg^ clones showed considerably higher LEF1 expression compared to FOXA1^KO^ and FOXA1^KO^A2A3^lo^ clones. SNAIL2/SLUG induction was restricted to the three FOXA1^KO^A2A3^lo/neg^ clones and did not occur in the FOXA1^KO^ clone 4F3. When inspecting intracellular distribution in FOXA1 wild-type (FOXA1^WT^) and FOXA1^KO^A2A3^lo/neg^ cells by immunofluorescence staining, SNAIL2/SLUG unfortunately remained below the detection limit, but LEF1 showed the expected nuclear localization. As anticipated, CADHERIN11 appeared membranous and marked cell boundaries (S6 Fig). We also examined the expression of epithelial marker proteins E-CADHERIN and CLAUDIN3. RNA and protein levels of E-CADHERIN, an adherens junction component, did not change in FOXA1^KO^ clones compared to wild-type controls (Fig 2B, S5 Fig). However, E-CADHERIN exhibited diffuse intracellular localization and was no longer concentrated at cell-cell interfaces in the absence of FOXA1 (S6 Fig). The tight junction protein CLAUDIN3 was downregulated specifically in FOXA1^KO^A2A3^neg^ clones and appeared mislocalized as well (Fig 2B, S5 and S6 Figs). The repeated observation that phenotypic and molecular changes are graded and most pronounced in FOXA1^KO^A2A3^neg^ clones is in agreement with the known redundancy among FOXA factors [29,30].

**Fig 3:**
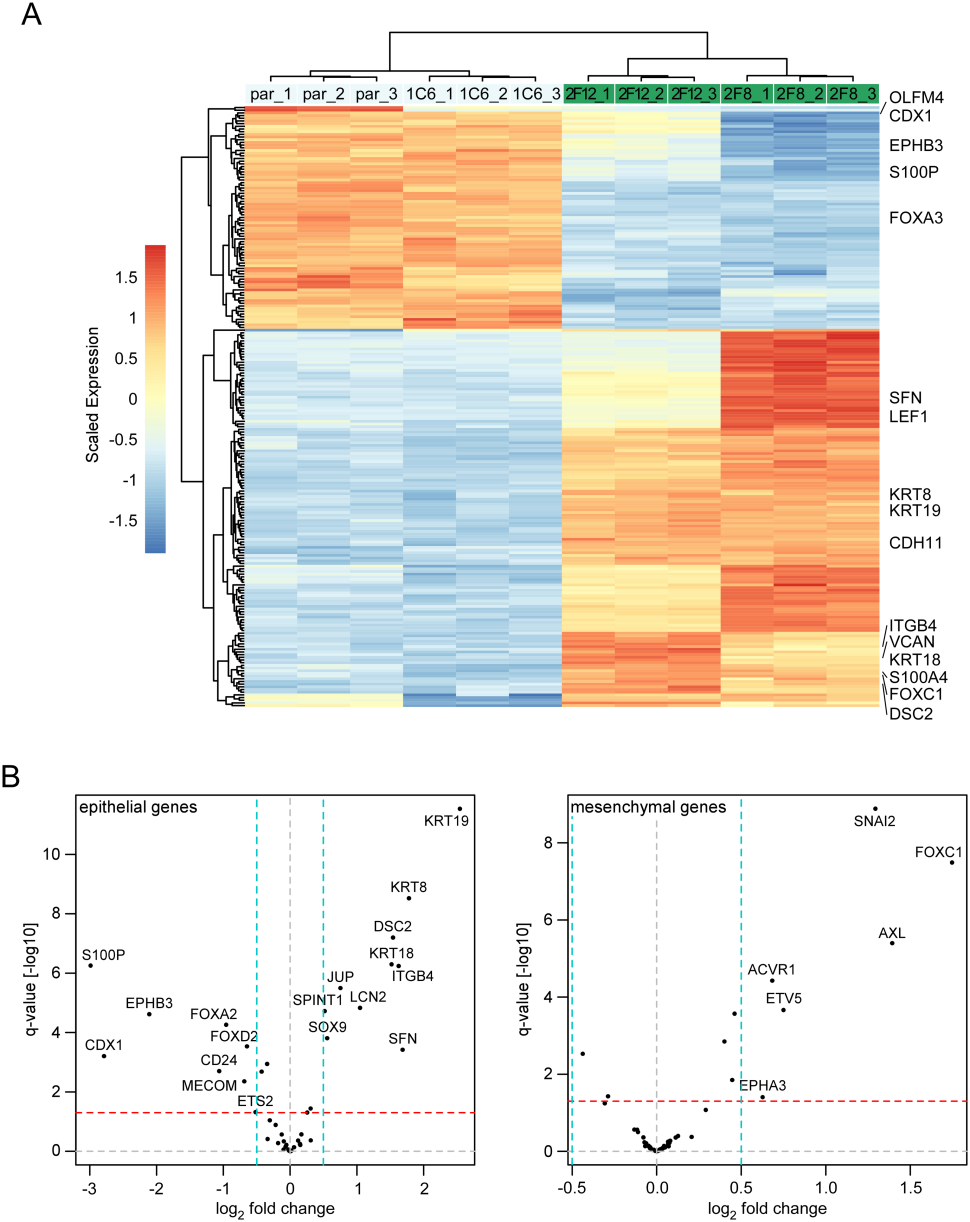
LS174T cell clones with absent/low FOXA expression show massive changes in gene expression and deregulation of a CRC EMT signature. (A) Hierarchical clustering of gene expression profiles for parental (par) LS174T cells and LS174T cell clones subjected to CRISPR/Cas9-mediated genome editing of the FOXA1 locus. Expression values were scaled gene-wise to have zero mean and unit variance across all samples. Rows and columns were hierarchically clustered using complete linkage. Scaled expression differences are indicated by color shading according to the color scale shown. 1C6: FOXA1^WT^; 2F12: FOXA1^KO^A2A3^lo^; 2F8: FOXA1^KO^A2A3^neg^. Cutoffs: p-value < 0.05 and log_2_ fold-change > 1.5. Three independent RNA isolates were examined for each cell type. Selected examples for epithelial and mesenchymal marker genes are highlighted. (B) Volcano plots to visualize changes in the expression of epithelial (left panel) and mesenchymal components (right panel) of an EMT gene signature for colon cancer [35]. The epithelial gene set of this signature is significantly deregulated (p = 0.0005, undirected gene set enrichment test), while the mesenchymal gene set is significantly upregulated (p = 0.05, directed gene set enrichment test). Dashed horizontal and vertical lines indicate thresholds for q-values and log_2_ fold change.

Altered cell morphology and the results of the targeted analyses suggested that FOXA-deficiency leads to a loss of epithelial features and the acquisition of more mesenchymal characteristics. To corroborate this we conducted global gene expression profiling experiments with the parental LS174T cells, the FOXA1^WT^ clone 1C6, and the FOXA1^KO^A2A3^lo/neg^ clones 2F8 and 2F12. Hierarchical clustering confirmed differences between the transcriptomes of all FOXA1^WT^ cells and the FOXA1^KO^A2A3^lo/neg^ clones (Fig 3A). Using thresholds of an adjusted p-value < 0.05 and a log_2_ fold change > 1.5 we found that 145 and 85 genes were significantly up- and downregulated, respectively, in FOXA1^KO^A2A3^lo/neg^ clones compared to FOXA1^WT^ cells (Fig 3A, S3 Table). Among the differentially expressed genes we identified additional examples of genes characteristic for intestinal epithelial cells (e. g. *CDX1*, *EPHB3*, *OLFM4*) [31,32], EMT, invasion and metastasis (e. g. *FOXC1*, *S100A4/FSP1*, *VCAN*) [1,33–35] (Fig 3A). As expected from the results described above, gene expression patterns in FOXA1^KO^A2A3^lo^ and FOXA1^KO^A2A3^neg^ clones were not identical but nevertheless closely related. Therefore, for subsequent comparisons the transcriptome data of FOXA1^KO^A2A3^lo/neg^ clones were merged. Gene set enrichment analysis based on the KEGG and REACTOME databases pinpointed the features “Pathways in cancer”, “Hemostasis”, “Focal adhesion”, “WNT signaling pathway”, and “Adherens junction” as the five most significantly upregulated in FOXA1^KO^A2A3^lo/neg^ clones (S4 Table). Top downregulated features are related to protein/RNA metabolism, and mitochondrial functions (S4 Table). To specifically interrogate gene expression changes related to EMT in FOXA1^KO^A2A3^lo/neg^ clones we focused on 45 and 54 genes (S5 Table) from an unsupervised analysis of 326 transcriptomes marking the EMT program in epithelial and mesenchymal colon tumors, respectively [35]. Expression of both gene sets turned out to be affected in FOXA1^KO^A2A3^lo/neg^ clones (Fig 3B). Epithelial genes were significantly up- and downregulated (p-value<0.0005, undirected gene set enrichment test), while the mesenchymal genes were preferentially upregulated (p-value<0.05, directed gene set enrichment test). In summary, an anticorrelation of SNAIL1 and FOXA factors can be observed in transcriptomes from colorectal tumors and CRC cell lines. Moreover, in CRC cellular EMT models, Snail1-HA expression leads to downregulation of FOXA factors and SNAIL1 appears to directly repress *FOXA1* by binding to its promoter. Furthermore, loss of FOXA expression in epithelial CRC cells results in more mesenchymal gene expression and morphology. Altogether these findings support the view that coordinate downregulation of FOXA genes by SNAIL1 facilitates EMT.

### FOXA1 and FOXA2 bind preferentially to enhancers in distal intergenic regions of EMT-associated epithelial genes

**Fig 4:**
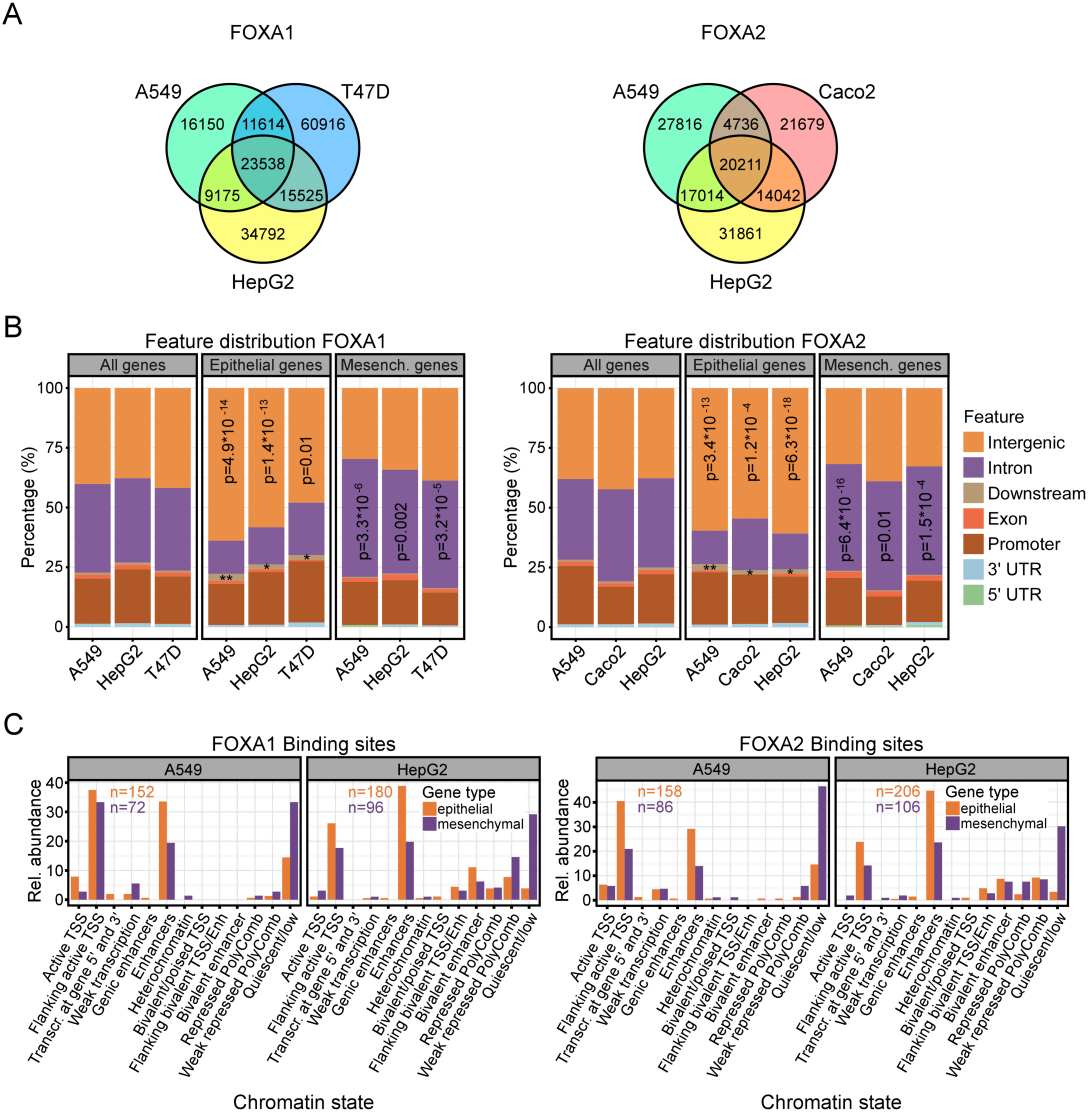
FOXA1 and FOXA2 bind preferentially to intergenic enhancer regions of epithelial genes. (A) Venn diagrams showing numbers of common and cell-type-specific ChIP-seq peaks of FOXA1 (left) and FOXA2 (right) in A549, HepG2, T47D and Caco2 cells. (B) Feature distribution of FOXA1 (left) and FOXA2 (right) ChIP-seq peaks for all genes (All genes) and genes differentially expressed in epithelial (Epithelial genes) or mesenchymal colon tumors (Mesench. genes). Peak locations were categorized into seven features according to their locations within 5’-UTR, 3’-UTR, promoter, exonic, downstream, intronic, or distal intergenic regions. Promoter regions were defined as 3 kb upstream and downstream of a transcription start site. Significant differences in the fractions of ChIP-seq peaks located in distal intergenic and intronic features for epithelial versus mesenchymal genes were assessed using a hypergeometric test relative to all genes. See S7 Fig, panel A for peak numbers. p-Values for FOXA1 occupancy at downstream regions of epithelial genes: A549: 1.076 x 10^−03^ (**); HepG2: 3.451 x 10^−02^ (*); T47D: 0.017 (*). p-Values for FOXA2 occupancy at downstream regions of epithelial genes: A549: 1.932 x 10^−03^ (**); Caco2: 0.044 (*); HepG2: 5.634 x 10^−02^ (*). (C) Relative (rel.) abundance of chromatin states for FOXA1 and FOXA2 ChIP-seq peak regions in distal intergenic regions of epithelial and mesenchymal genes for A549 and HepG2 cells. Genomic locations were annotated according to HMM on chromatin states. Bars denote the relative abundance of ChIP-seq peaks (in %) for 13 out of 15 states represented in the peak regions. The potential functional impact of transcription factor binding decreases from left to right: binding to an active transcription start site (TSS) to binding in a quiescent region. The numbers inside the bar plots indicate the total number of peaks for each condition.

To further explore the potential impact of FOXA factors on epithelial and mesenchymal gene expression we aimed to elucidate common and cell-type-specific regulatory patterns of FOXA1 und FOXA2 on a genome-wide level. For this, we collected six ChIP-Seq datasets for FOXA1 and FOXA2 in human A549 lung adenocarcinoma cells, HepG2 hepatocellular carcinoma cells, T47D breast cancer cells, and Caco2 CRC cells. The data was reanalyzed using bwa-mem and MACS2 for read alignment and peak calling [36,37]. We found between 67,000 and 129,000 peaks per cell type and transcription factor with a false discovery rate corrected p-value < 0.01. In line with previous results [38] the peak sites were specific to the cell types with only 30–40% of peaks being common to all (Fig 4A). The annotation of ChIP-seq peak locations with respect to genomic features showed a prevalent association of both FOXA1 and FOXA2 with distal intergenic, intronic and promoter regions irrespective of the cell type (Fig 4B, “All genes”). We then asked whether FOXA1 and FOXA2 ChIP-seq peak distribution deviated from this pattern at the colon cancer EMT signature genes introduced above [35]. Between 222 and 429 FOXA1 and FOXA2 ChIP-seq peaks were associated with either the epithelial or mesenchymal genes per condition, indicating multiple FOXA binding regions to be present at each gene (S7 Fig, panel A). A hypergeometric test showed a significant difference in the preferential location of the FOXA1 and FOXA2 ChIP-seq peaks. They were enriched in distal intergenic and downstream regions for epithelial genes and in intronic regions for mesenchymal genes (Fig 4B). To further investigate whether the differences in FOXA1 and FOXA2 genomic localizations near epithelial and mesenchymal genes are of functional significance, we annotated the intergenic ChIP-seq peak loci with their cell-type-specific chromatin states. The latter were predicted by a multivariate Hidden Markov Model (ChromHMM v1.10) from the Roadmap epigenomics project [39,40]. In brief, a 15-state HMM was learned on 127 epigenomes by virtually concatenating consolidated data corresponding to the core set of 5 chromatin marks. The HMM captured their key interactions from which the posterior probability of each state per genomic bin and reference epigenome was calculated. Such chromatin marks are highly specific to the respective cells or tissues. Therefore, we applied the HMM to A549 and HepG2 cells which are contained in the 127 epigenomics project data (Samples E114 and E118) and annotated FOXA1 and FOXA2 ChIP-seq peaks according to the most likely state of the bin which contained the peak summit. This showed that FOXA1 and FOXA2 preferentially bind to quiescent sites in the distal intergenic regions of mesenchymal genes (Fig 4C; S8 Fig, panel A). In contrast, at epithelial genes, FOXA1 and FOXA2 predominantly associate with regions flanking active transcription start sites and with enhancers (Fig 4C; S8 Fig, panel A). Moreover, FOXA1 and FOXA2 ChIP-seq peaks associated with epithelial genes have significantly more reads than those associated with mesenchymal genes irrespective of cell type and transcription factor (S8 Fig, panel B).

To further test the idea that there is a special link between FOXA proteins and regulatory elements at epithelial genes, we performed a bootstrapping approach to estimate whether the number of FOXA1/FOXA2 ChIP-seq peaks at epithelial genes is significantly high or low. We repeatedly selected by chance N out of all 22,000 annotated genes and counted the number of associated peaks, where N is 45 or 54, i.e. the number of epithelial or mesenchymal genes, respectively. An example for the resulting distribution of associated peak numbers from 10,000 trials for the FOXA1 ChIP-seq data from HepG2 cells is depicted in S7 Fig, panel B where the red lines indicate the number of associated peaks for the epithelial and mesenchymal genes. Corresponding p-values were calculated from fitting a skewed normal distribution to the histogram and are displayed as well. Remarkably, the group of epithelial genes but not the mesenchymal gene group has significantly more peaks associated with it than random groups of genes. Thus, based on the combined information derived from the various analyses of the ChIP-Seq datasets a functional impact of FOXA1 and FOXA2 on epithelial gene expression is highly likely.

### Transcriptional enhancers at the epithelial genes *CDH1*, *CDX2* and *EPHB3* are regulated by FOXA factors

FOXA expression correlated with that of epithelial genes, and FOXA1 and FOXA2 were found to be associated with regulatory regions at epithelial genes. These observations prompted us to investigate the potential consequences of FOXA downregulation on the structure and function of transcriptional enhancers at epithelial genes in colorectal cancer. To identify candidate cis-regulatory elements targeted by FOXA factors, we performed literature search, interrogated the ECR browser for evolutionary conserved regions (ECRs), and integrated this information with the results of the FOXA1 and FOXA2 ChIP-seq analysis. As a result, we examined in detail the impact of FOXA factors on enhancer regions at the *CDH1*, *CDX2* and *EPHB3* genes which are among the signature genes of epithelial colon tumors and which are downregulated in Snail1-expressing LS174T cells (S11 Fig, panels B, C) [27,35,41].

**Fig 5:**
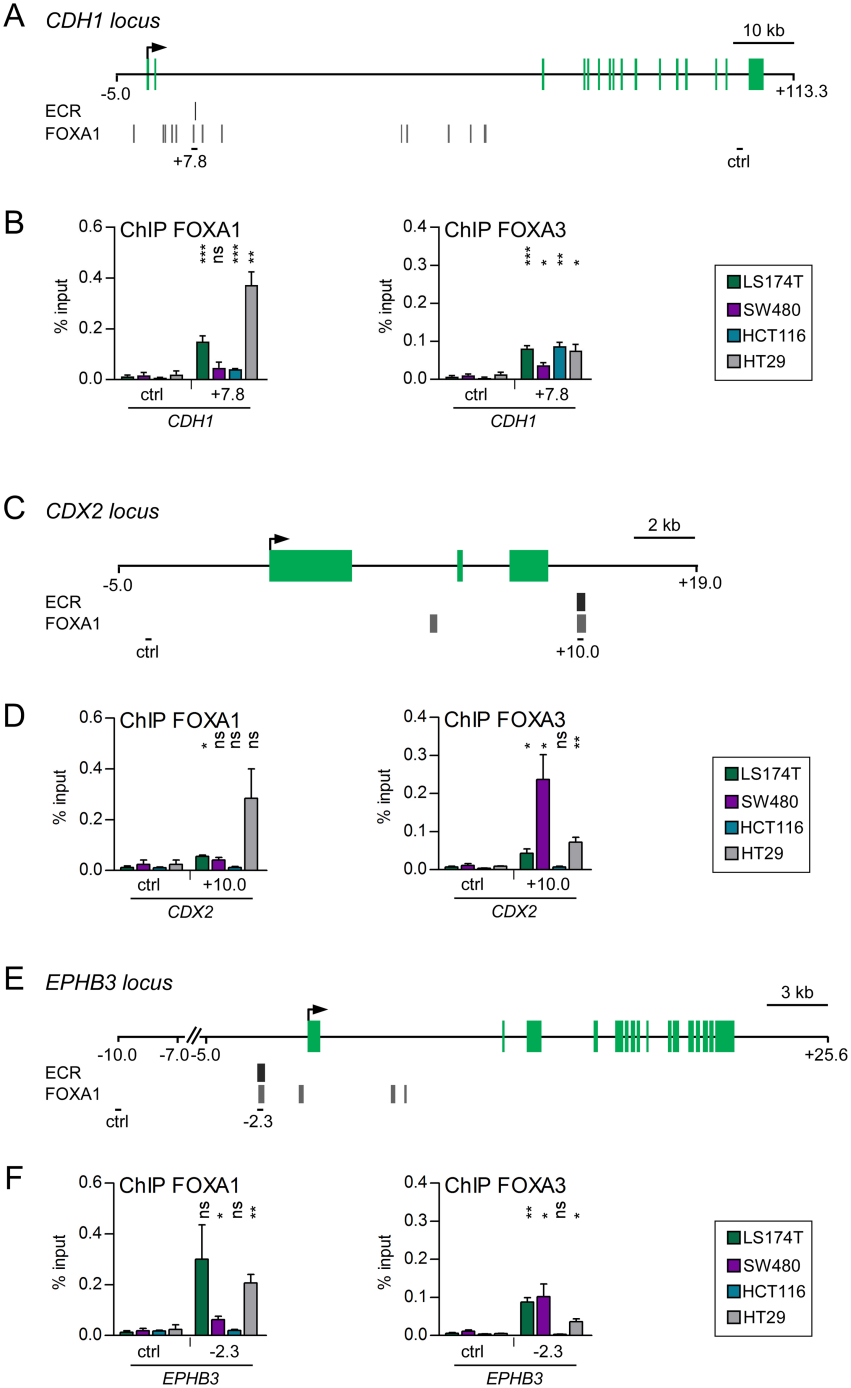
FOXA1 and FOXA3 are regulators of the *CDH1*, *CDX2* and *EPHB3* enhancers. (A, C, E) Schemes of the *CDH1* (A), the *CDX2* (C), and the *EPHB3* (E) gene loci. Gene annotation is based on the Ensembl genome browser. Exons are depicted in green. Transcriptional start sites are indicated by arrows. The positions of ECRs with predicted FOX binding motifs and signals from FOXA1 ChIP-seq analyses from the ENCODE project are shown below the schemes of the gene loci. The PCR-amplified regions analyzed in ChIP experiments are indicated by black bars. (B, D, F) ChIP analyses to test for binding of FOXA1 and FOXA3 at the *CDH1* (B), the *CDX2* (D) and the *EPHB3* (F) loci in the indicated CRC cell lines. Data were calculated as percent input. Shown are the mean and SEM; n≥3. Statistical significance was calculated relative to the control regions at the respective gene loci.

Alotaibi et al. identified two enhancers at the *CDH1* locus at +7.8 kb and +11.5 kb that were activated during mesenchymal-epithelial transition in NMuMG cells [42]. Analyses of ECRs of the *CDH1* locus revealed DNA sequence conservation with a predicted FOXA binding motif at +7.8 kb (S9 Fig, panel A, FOXA motif I). Moreover, the ENCODE data base lists several FOXA1 ChIP-seq peaks at the *CDH1* locus in different cell lines, especially in the second intron. Notably, at the +7.8 kb enhancer region a FOXA1 ChIP-seq signal overlapped with an ECR (Fig 5A). To find out if FOXA factors could be involved in the function of the intronic *CDH1* enhancer in CRC cells, we performed ChIP analyses. Since FOXA1 and FOXA2 are highly similar proteins and can act redundantly [12], we focused on FOXA1 and FOXA3 occupancy. FOXA3 occupied the *CDH1* +7.8 kb enhancer in all CRC cells (Fig 5B), albeit the level of enrichment was slightly lower in SW480 cells. Moreover, in LS174T and HT29 cells showing high FOXA1 expression (Fig 1B and 1C) this region was also bound by FOXA1. Interestingly, in the panel of CRC cells used for these analyses, LS174T and HT29 cells showed highest *CDH1* expression (S10 Fig, panel A). In fact, the combined patterns of FOXA1/A3 occupancy at the *CDH1* +7.8 kb enhancer closely reflects *CDH1* expression levels in the four CRC cell lines under investigation (S10 Fig, panel A). This raises the possibility that FOXA factors contribute to the control of *CDH1* expression in CRC cells through their occupancy of the *CDH1* +7.8 kb enhancer.

The CDX2 transcription factor is a key regulator of cellular identity and differentiation in the intestinal epithelium [25,43]. Loss of CDX2 expression has been associated with poor tumor differentiation [44–46]. Watts et al. showed that in adult mouse liver a *Cdx2* downstream enhancer element is occupied by Foxa2 [47]. Moreover, the DNA sequence of this region is evolutionary conserved, contains FOXA binding motifs (S9 Fig, panel B), and coincides with ENCODE FOXA1 ChIP-seq peaks in HepG2 hepatocellular carcinoma and T-47D breast cancer cells (Fig 5C). To test for binding of FOXA proteins at this site of the *CDX2* locus in CRC cells we performed ChIP analyses. Indeed, this region was occupied by FOXA1 in LS174T and HT29 cells. Furthermore, FOXA3 was bound at the *CDX2* +10.0 kb region in LS174T, SW480 and HT29 cells (Fig 5D). Even though binding did not correlate with *CDX2* expression in the CRC cells (S10 Fig, panel B), this result suggests that FOXA proteins act upon a transcriptional enhancer at the *CDX2* locus in CRC cells.

In CRC, the tumor suppressor gene *EPHB3* was shown to promote cellular adhesion and to prevent tumor cell spreading [48,49]. As previously shown, EPHB3 expression is controlled by an enhancer at -2.3 kb at the *EPHB3* locus [50]. This enhancer integrates input from ETS proteins, Notch and Wnt/β-Catenin signaling, and the E-box binding protein ASCL2 [27,50–52]. Notably, this region also contains predicted FOXA binding motifs (S9 Fig, panel C) and FOXA1 ChIP-seq peaks are present in HepG2 cells (Fig 5E). In line with this, we detected by ChIP FOXA1 occupancy at the *EPHB3* −2.3 kb enhancer in LS174T and HT29 cells (Fig 5F). Furthermore, FOXA3 was found to be present at the *EPHB3* enhancer at -2.3 kb in all CRC cells except for HCT116 cells (Fig 5F). Similar to *CDX2*, occupancy of FOXA1 and FOXA3 did not correlate with expression of *EPHB3* (S10 Fig, panel C). Nonetheless, the results from the ChIP analyses suggest that FOXA1 and FOXA3 can affect *EPHB3* enhancer activity.

On the one hand, we observed that upon Snail1 expression in CRC cells the expression of FOXA proteins is downregulated. On the other hand, FOXA proteins act on transcriptional enhancers of the epithelial genes *CDH1*, *CDX2* and *EPHB3*. Therefore, we were interested to examine if occupancy of FOXA factors at these enhancers changes upon Snail1 expression in LS174T cells. Indeed, at all three enhancers FOXA1 binding was diminished over time upon Snail1 induction (S11 Fig). This was paralleled by downregulation of *CDH1*, *CDX2*, and *EPHB3*, even though *CDX2* expression eventually recovered as was noticed before [27,41].

**Fig 6:**
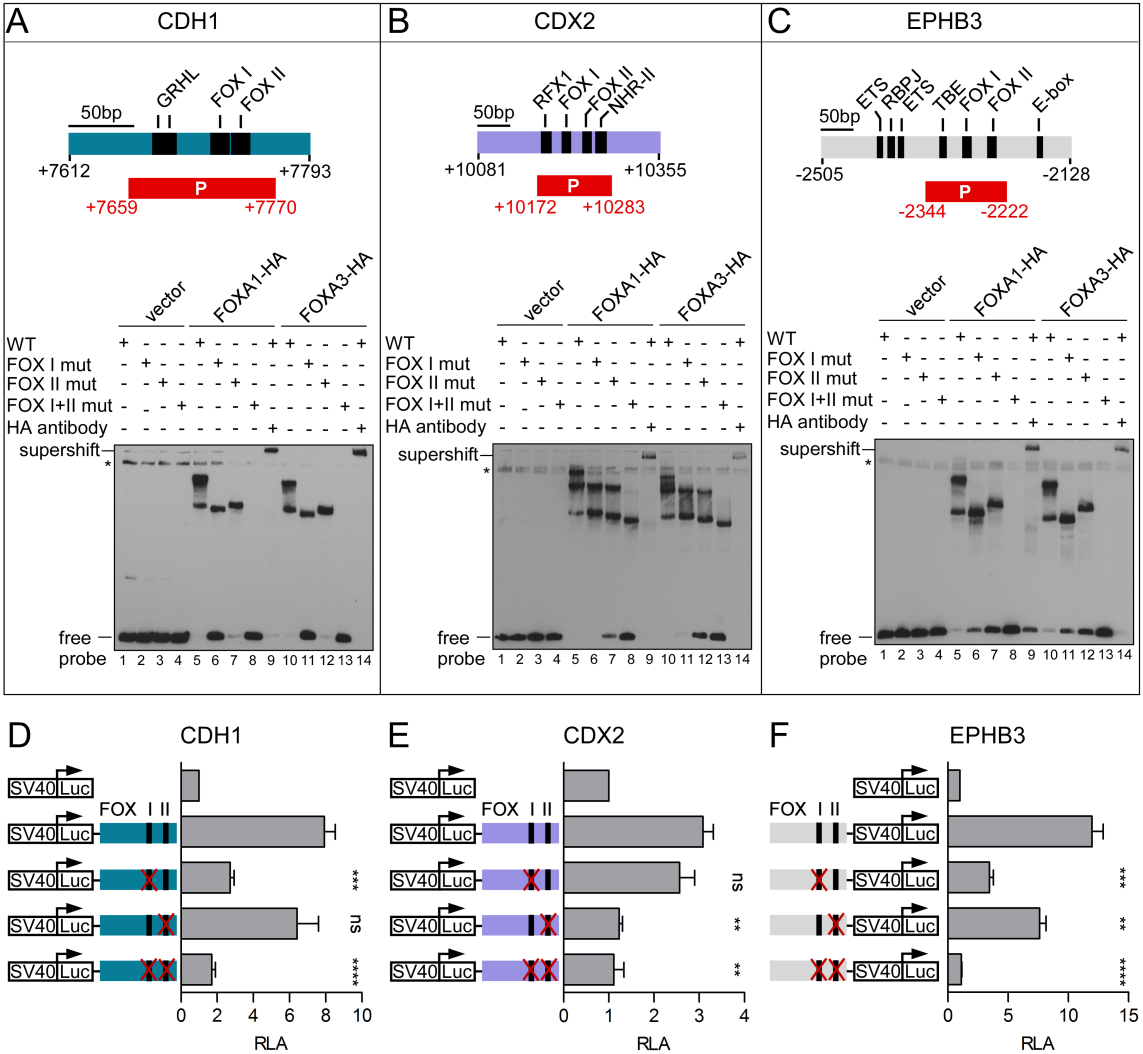
*CDH1*, *CDX2* and *EPHB3* enhancer activity is dependent on FOXA binding. (A) (Top) Scheme of the ECR at +7.8 kb at the *CDH1* locus with the described tandem GRHL binding sites [42] and two potential FOX binding motifs. (Bottom) EMSA to test for *in vitro* binding of FOXA1 and FOXA3 to the *CDH1* +7.8 kb enhancer. Asterisks: non-specific bands. mut: mutated, P: probe. (B) (Top) Scheme of the *CDX2* +10.0 kb enhancer with the described RFX1, FOXA and nuclear hormone receptor-II (NHR-II) transcription factor binding sites [47]. (Bottom) EMSA to test for *in vitro* binding of FOXA1 and FOXA3 to the *CDX2* +10.0 kb enhancer. (C) (Top) Scheme of the *EPHB3* −2.3 kb enhancer with its known transcription factor binding sites. TBE: TCF/LEF-binding element. (Bottom) EMSA to test for *in vitro* binding of FOXA1 and FOXA3 to the *EPHB3* −2.3 kb enhancer. (D, E, F) Luciferase reporter assay in LS174T cells with constructs covering the *CDH1* (D), the *CDX2* (E), and the *EPHB3* (F) enhancer. Mutations of the respective FOX binding motifs are indicated by red crosses. Shown is the mean and SEM; n≥3. RLA: relative luciferase activity. Statistical significance was calculated relative to the wild-type luciferase reporter constructs.

Since occupancy of the *CDH1* +7.8 kb and *EPHB3* −2.3 kb enhancers by FOXA factors had not yet been described, we validated binding of FOXA1 and FOXA3 to the predicted FOXA binding motifs at these enhancers *in vitro* by EMSAs. As a control, we also analyzed *in vitro* binding of FOXA1 and FOXA3 to the *CDX2* +10.0 kb enhancer. At the *CDH1* enhancer we detected two protein::DNA complexes (Fig 6A, lane 5 and 10). This result was unexpected, since only one highly conserved binding motif was predicted (S9 Fig, panel A, FOX I motif). However, manual inspection of the sequence revealed a second, less conserved potential binding motif (S9 Fig, panel A, FOX II motif). Indeed, mutation of either one of the two FOX motifs led to disappearance of one of the two protein::DNA complex while the other persisted (Fig 6A, lane 6/7 and 11/12), suggesting that these two motifs can be bound by FOXA1 and FOXA3. The difference in the migration pattern of the protein::DNA complexes upon mutation of either the first (FOX I) or the second (FOX II) binding motif probably is due to the DNA-bending capacity of FOXA proteins [53] which is known to influence migration of protein::DNA complexes [54]. No binding of FOXA1 or FOXA3 to the *CDH1* enhancer was detected upon double mutation of the two FOX motifs (Fig 6A, lane 8 and 13). Specificity of the binding reaction was demonstrated by the occurrence of a supershift upon addition of an anti-HA antibody (Fig 6A, lane 9 and 14). At the *EPHB3* enhancer we detected a similar FOXA1 and FOXA3 binding pattern, validating binding of FOXA1 and FOXA3 to the two predicted FOX motifs (Fig 6C). Surprisingly, at the *CDX2* enhancer we observed three protein::DNA complexes (Fig 6B, lane 5 and 10), even though only two FOXA binding motifs were described [47]. By manual inspection of the sequence we could not find any evidence for a further potential FOXA binding motif. Possibly, at the *CDX2* enhancer FOXA1 and FOXA3 can bind to a motif deviating from the consensus FOXA motif. Irrespective of this, the disappearance of one and two protein::DNA complexes upon individual and double mutation of the two FOXA motifs, respectively, validated that both known FOXA motifs at the *CDX2* enhancer can be bound by FOXA1 and FOXA3 (Fig 6B). To test the importance of the FOXA binding sites for *CDH1*, *CDX2* and *EPHB3* enhancer activity, we performed luciferase reporter assays in LS174T cells (Fig 6D–6F). The respective enhancer activity was lost almost completely upon mutation of the *CDH1* FOX I motif and the *CDX2* FOX II motif, while mutations of the other FOX motifs were neutral (Fig 6D and 6E). *EPHB3* enhancer activity was diminished upon mutation of either one of the two FOX binding motifs and enhancer activity was entirely wiped out when both FOX binding sites were mutated (Fig 6C). Thus, these analyses corroborated binding of FOXA1 and FOXA3 to the *CDH1* +7.8 kb, the *CDX2* +10.0 kb and the *EPHB3* −2.3 kb enhancers and demonstrated the dependence of *CDH1*, *CDX2* and *EPHB3* enhancer activity on FOXA binding.

### Interfering with FOXA function compromises *CDH1*, *CDX2*, and *EPHB3* enhancer structure

**Fig 7:**
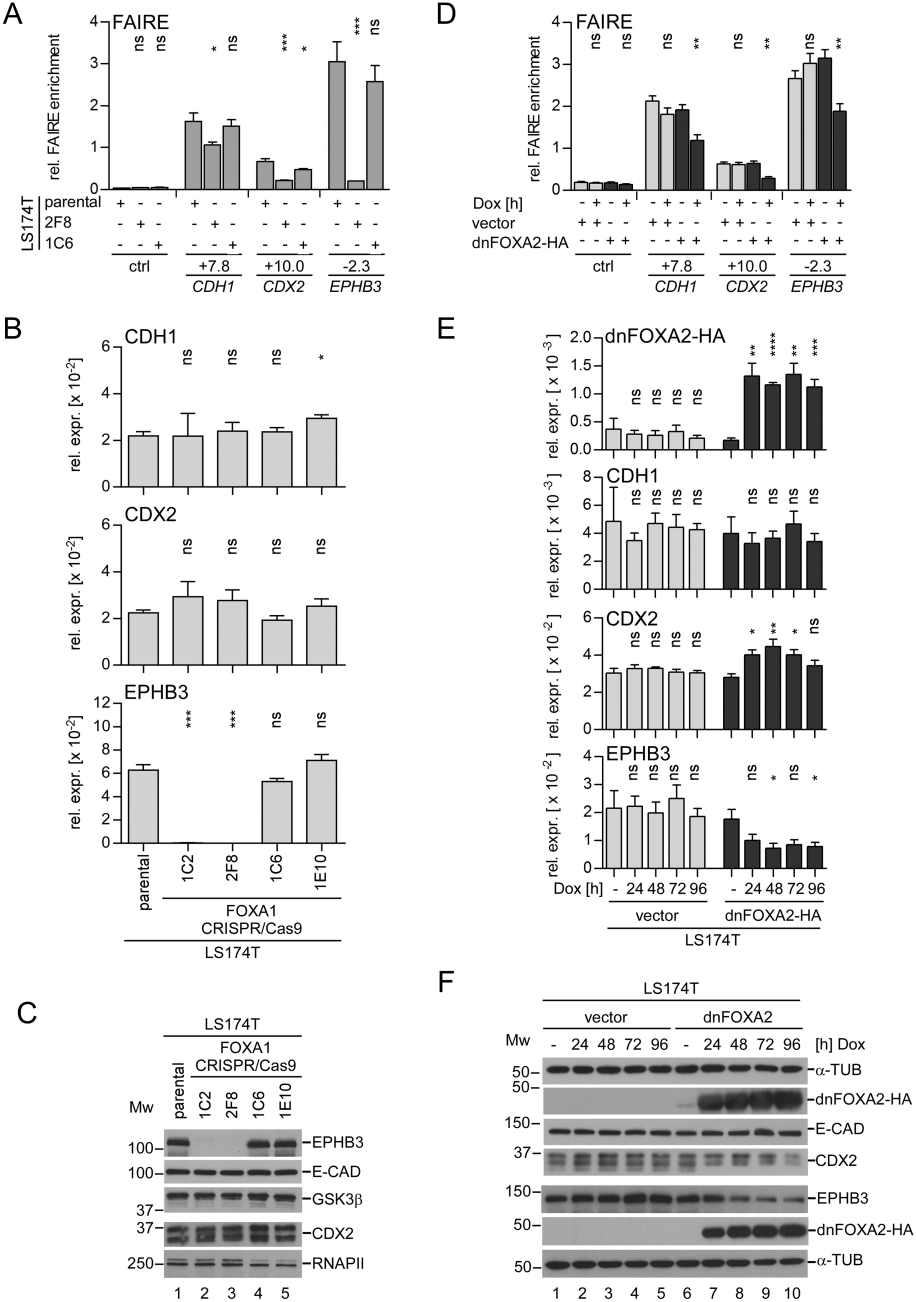
Interfering with FOXA function alters chromatin structure at the *CDH1*, *CDX2*, and *EPHB3* enhancers. (A) FAIRE analyses of the *CDH1* +7.8 kb, the *CDX2* +10.0 kb and the *EPHB3* −2.3 kb enhancers in parental LS174T cells, the FOXA1^KO^A2A3^neg^ cell clone 2F8, and the FOXA1^WT^ cell clone 1C6. As control (ctrl) the *AXIN2* locus at -11.0 kb was analyzed. Data are given as relative (rel.) FAIRE enrichment; n = 4. (B) qRT-PCR analyses to assess *CDH1*, *CDX2* and *EPHB3* relative expression (rel. expr.) levels in parental LS174T cells, FOXA1^KO^A2A3^neg^ cell clones (1C2, 2F8), and FOXA1^WT^ cell clones (1C6, 1E10) as indicated. For *CDH1*, the results shown here were extracted from the data set presented in S5 Fig. Data are shown as mean and SEM; n = 3. (C) Assessment of E-CADHERIN (E-CAD), CDX2 and EPHB3 protein levels by Western Blotting in cytoplasmic (E-CAD, EPHB3, GSK3β) and nuclear fractions (CDX2, RNAPII) derived from parental LS174T cells, FOXA1^KO^A2A3^neg^ cell clones (1C2, 2F8), and FOXA1^WT^ cell clones (1C6, 1E10). GSK3β and RNA polymerase II (RNAPII) immunodetections served as loading controls. M_W_ = molecular weight in kDa. (D) FAIRE analyses of the *CDH1* +7.8 kb, the *CDX2* +10.0 kb and the *EPHB3* −2.3 kb enhancers in LS174T cells stably transduced with Dox-inducible retroviral control or dnFOXA2-HA expression vectors treated without or with Dox for 96h. As control (ctrl) the *EPHB3* locus at -7.0 kb was analyzed. Data are given as relative (rel.) FAIRE enrichment; n = 4. (E) qRT-PCR analyses to assess *dnFOXA2-HA*, *CDH1*, *CDX2*, and *EPHB3* relative expression (rel. expr.) levels in LS174T cells stably transduced with Dox-inducible retroviral control or dnFOXA2-HA expression vectors. Data are shown as mean and SEM; n = 4. For detection of dnFOXA2-HA expression a primer pair amplifying cDNA from endogenous full-length FOXA2 as well as truncated dnFOXA2-HA transcripts was used. (F) Western Blot analyses showing equal E-CADHERIN (E-CAD) levels and reduced CDX2 and EPHB3 protein expression upon Dox-induced dnFOXA2-HA expression in LS174T cells stably transduced with Dox-inducible retroviral control or dnFOXA2-HA expression vectors. M_W_ = molecular weight in kDa. α-TUBULIN (α-TUB) immunodetection served as loading control.

FOXA proteins can influence enhancer function and gene expression in different ways. They can act as classical transcription factors that promote transcription by interaction with the basal transcription machinery. In addition, FOXA proteins can function as pioneer factors, whereby they make use of a protein domain at their C-termini which can interact with the core histones H3 and H4 [55]. Thereby, FOXA factors have the potential to displace histones, to open compacted chromatin and thus to allow other transcription factors to get access to their DNA binding sites [56]. In order to gain more insight into the role of FOXA proteins at the *CDH1*, *CDX2* and *EPHB3* enhancers, we analyzed how their structure was affected when interfering with FOXA function. For these experiments we employed parental LS174T cells, the FOXA1^WT^ cell clone 1C6 and the FOXA1^KO^A2A3^neg^ cell clone 2F8 (Fig 7A–7C). Additionally, we expressed a Dox-inducible HA-tagged dominant negative (dn) FOXA2 protein in LS174T cells (Fig 7D–7F). The dnFOXA2-HA protein lacks the FOXA2 transactivation function but has the same DNA binding specificity as FOXA1 and FOXA3, and readily occupies the *CDH1*, *CDX2*, and *EPHB3* enhancers in living cells (S12 Fig). To study enhancer structure in different states of FOXA functionality we performed formaldehyde-assisted isolation of regulatory elements (FAIRE) [57]. As expected, the *CDH1* +7.8 kb, the *CDX2* +10 kb and the *EPHB3* −2.3 kb enhancer regions showed elevated FAIRE enrichment signals compared to a negative control region in LS174T cells and their derivatives with unperturbed FOXA expression and function. This indicates that the chromatin at the *CDH1*, *CDX2* and *EPHB3* enhancer regions is in an open conformation. However, differences between the three enhancer loci were observed. When compared to the control region, the FAIRE signal was approximately three times higher at the *CDX2* enhancer, ten times higher at the *CDH1* enhancer and fifteen times higher at the *EPHB3* enhancer (Fig 7A and 7D). More importantly, however, irrespective of the differences in relative FAIRE enrichment at the three different enhancers, the FAIRE signals were decreased at all enhancer regions in FOXA1^KO^A2A3^neg^ cells and upon Dox-induced dnFOXA2-HA expression (Fig 7A and 7D). Thus, the absence of FOXA proteins and perturbing FOXA function in LS174T cells by expression of a dnFOXA-HA protein consistently induced chromatin condensation at the *CDH1*, *CDX2* and *EPHB3* enhancer regions.

We also tested whether alterations in enhancer structure translated into changes in gene expression. As observed before (Fig 2) E-CADHERIN mRNA and protein levels were not changed in FOXA1^KO^A2A3^neg^ cells and upon dnFOXA2-HA induction (Fig 7B, 7C, 7E and 7F). Similarly, CDX2 expression was not affected by FOXA-deficiency (Fig 7B and 7C). However, it was transiently increased on mRNA but decreased on protein level in cells expressing dnFOXA2-HA (Fig 7E and 7F). In contrast, EPHB3 expression was reduced by 57% upon expression of dnFOXA2-HA (Fig 7D and 7E), and it was entirely abrogated when all three FOXA factors were missing (Fig 7B and 7C). Thus, FOXA proteins are required to maintain the transcription of *EPHB3*, but not that of *CDH1* and *CDX2*. Nonetheless, enhancer structure at all three genes depends on fully functional FOXA1, FOXA2, and FOXA3.

### FOXA binding is essential for *EPHB3* enhancer activity and EPHB3 expression

**Fig 8:**
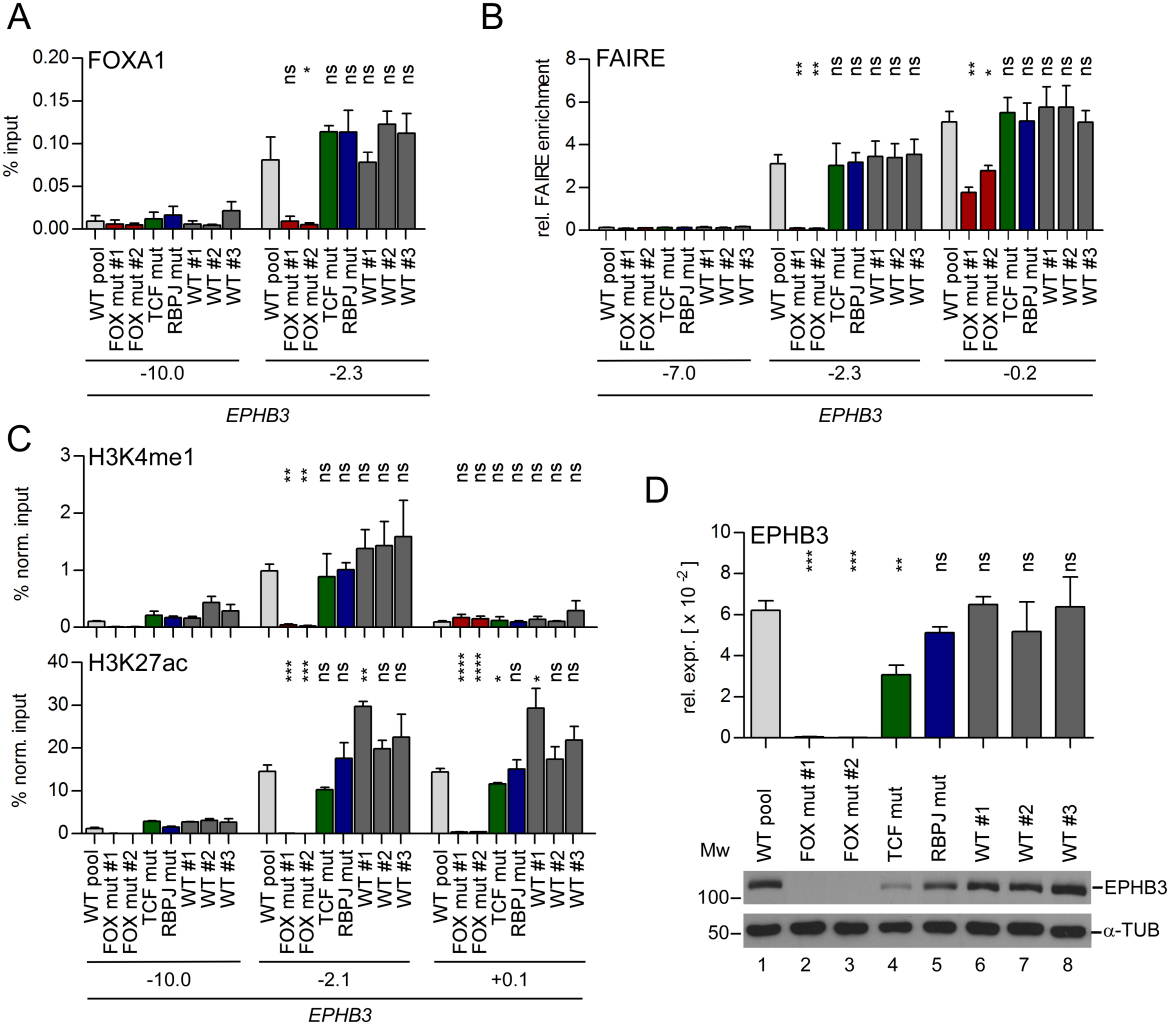
Mutation of the FOX binding sites at the *EPHB3* enhancer leads to chromatin compaction at the enhancer and promoter and abolishes *EPHB3* expression. (A) ChIP analysis to monitor FOXA1 occupancy at the *EPHB3* locus in LS174T cell clones genome-edited with the CRISPR/Cas9 system. Data are given as percent input. Shown is the mean and SEM; n≥3. Statistical significance was calculated relative to the LS174T wild-type cell pool. (B) FAIRE analyses to test chromatin compaction at the *EPHB3* enhancer and promoter in LS174T cell clones genome-edited with the CRISPR/Cas9 system. Data are given as relative (rel.) FAIRE enrichment; n = 3. Statistical significance was calculated relative to the LS174T wild-type cell pool. (C) ChIP analyses to monitor presence of the histone modifications H3K27ac and H3K4me1 at the *EPHB3* locus in LS174T cell clones genome-edited with the CRISPR/Cas9 system. Data are given as percent input normalized (norm.) to H3 to compensate for variations in nucleosome density; n≥3. Statistical significance was calculated relative to the LS174T wild-type cell pool. (D) qRT-PCR (upper) and Western Blot (lower) analyses to assess EPHB3 expression in LS174T cell clones with mutations in transcription factor binding sites at the *EPHB3* enhancer. Data are shown as mean and SEM; n = 3; rel. expr.: relative expression. M_W_ = molecular weight in kDa. α-TUBULIN (α-TUB) immunodetection served as loading control.

The results so far suggest that FOXA proteins are important for maintaining *CDH1*, *CDX2* and *EPHB3* enhancer structures. However, it cannot be strictly excluded that combined FOXA-deficiency exerts indirect effects on these regulatory elements through deregulation of other genes. The same applies to dnFOXA2-HA. In addition, due to its ability to bind to DNA, dnFOXA2-HA might function as a place holder protein. Thereby, it could contribute to the maintenance of an open chromatin structure that allows other transcription factors to bind to the *CDH1*, *CDX2* and *EPHB3* enhancers and support gene expression. Accordingly, we investigated how structure of an enhancer and expression of a linked gene were affected when binding of FOXA proteins at a defined chromosomal location was prevented. We chose the *EPHB3* enhancer and used CRISPR/Cas9-mediated genome editing to generate mutations in the FOX binding motifs in the genome of LS174T cells (S13 Fig, panels A, B). For comparison, we utilized a similar approach to mutate the RBPJ and TCF binding motifs at the *EPHB3* enhancer (S13 Fig, panels A, B). We validated by EMSA that the mutations introduced abolished binding of the respective transcription factors *in vitro* (S13 Fig, panels C-G). After the identification of cell clones in which the desired mutations had been introduced on both alleles (S14 Fig, S2 Table), we tested for FOXA1 binding at the *EPHB3* enhancer. As expected, mutation of the FOX binding sites completely abolished FOXA1 enhancer occupancy (Fig 8A). In contrast, mutation of the TCF and RBPJ motifs had no impact on the interaction of FOXA1 with the *EPHB3* enhancer. Next, we investigated by FAIRE chromatin accessibility at the *EPHB3* enhancer and promoter elements, and by ChIP the distribution of histone modifications characteristic for different functional states of enhancers and promoters. While mutations of the RBPJ and TCF binding sites did not affect the chromatin state at the *EPHB3* enhancer, disrupting FOX binding sites led to complete chromatin condensation (Fig 8B). Notably, chromatin condensation was observable not only at the *EPHB3* enhancer but also at the *EPHB3* promoter (Fig 8B, position -0.2). The level of the general enhancer mark H3K4me1 was unchanged in cell clones with mutated RBPJ and TCF binding sites, the wild-type control cell clones and the wild-type cell pool (Fig 8C). However, in cell clones with mutated FOX binding sites, H3K4me1 was absent from the *EPHB3* enhancer. We also analyzed the distribution of the histone modification H3K27ac, which is indicative of active enhancers and promoters. This revealed somewhat variable levels of H3K27ac at the *EPHB3* enhancer and promoter in wild-type cells. Nonetheless, significantly reduced H3K27ac levels were detected at the *EPHB3* promoter when the TCF binding site at the *EPHB3* enhancer was mutated. Even more strikingly, H3K27ac was completely undetectable at the *EPHB3* promoter and the enhancer upon mutation of the two FOX binding sites at the *EPHB3* enhancer (Fig 8C). Of note, the graded effects on *EPHB3* promoter and enhancer structure upon mutation of different transcription factor binding sites were paralleled by corresponding changes in mRNA and protein expression levels. In the LS174T wild-type cell pool, the control cell clones, and the cell clone with a mutated RBPJ binding site, *EPHB3* expression was not significantly altered. Upon mutation of the TCF binding site at the *EPHB3* enhancer, *EPHB3* expression was reduced but not entirely turned off. Remarkably, mutation of the two FOX binding sites at the *EPHB3* enhancer completely prevented *EPHB3* expression (Fig 8D). To rule out that the absence of *EPHB3* expression was a result of changes in FOXA expression or due to altered activity of other *EPHB3* activators like Notch- and Wnt/β-Catenin signaling, we performed expression analyses. The levels of FOXA1, FOXA2, and FOXA3 were similar in all analyzed cell clones, except for lower FOXA3 expression in the wild-type control cell clone #2 (S15 Fig, panels A, B). In this cell clone also the expression of the FOXA1/FOXA2 target gene *MUC2* was lower compared to the other cell clones and the wild-type cell pool (S15 Fig, panel C). To test the functionality of Notch- and Wnt/β-Catenin signaling, we analyzed the abundance of the Notch1 intracellular domain (NICD), indicative of an activated Notch receptor, and of TCF7L2 and β-CATENIN, mediating Wnt/β-Catenin signaling in the intestine (S15 Fig, panel B). Furthermore, we checked expression of the Notch- and Wnt/β-Catenin target genes NRARP and AXIN2, respectively (S15 Fig, panel C). NICD, TCF7L2 and β-CATENIN levels were similar and the expression of NRARP and AXIN2 varied only little between the different cell clones. From this we conclude that there are no differences in Notch- and Wnt/β-catenin signaling activity that could explain the effects seen at the *EPHB3* enhancer in the LS174T cell clones with mutated FOX binding sites. Taken together, preventing FOXA binding to the *EPHB3* enhancer destroys enhancer structure and function and precludes *EPHB3* expression.

### Overexpression of FOXA1 and FOXA3 reveals their pioneer function at the *CDX2* and *EPHB3* enhancer elements

To complement the FOXA loss of function studies, we next investigated how FOXA1 and FOXA3 overexpression would affect chromatin structure at the *CDH1*, *CDX2* and *EPHB3* enhancers. For this, we generated derivatives of HCT116 CRC cells with Dox-inducible expression of HA-epitope-tagged FOXA1 and FOXA3. The HCT116 cell line was chosen because it has little or no endogenous expression of FOXA1. Hence, the *CDH1*, *CDX2* and *EPHB3* enhancers were found to be devoid of FOXA1 in HCT116 cells (Figs 1 and 5). Likewise, FOXA3 is absent from *CDX2* and *EPHB3* enhancers. Furthermore, chromatin at the *CDX2* and *EPHB3* enhancers appears to be completely condensed (see below) and both genes are silent in this cell line. Thereby, at the *CDX2* and *EPHB3* loci the impact of inducible FOXA1-HA and FOXA3-HA expression in a naïve chromatin setting could be studied. In addition, the *CDH1* gene which displayed occupancy of the +7.8 kb enhancer by FOXA3 and which is expressed in HCT116 cells (Fig 5 and S10 Fig), served as an informative control.

**Fig 9:**
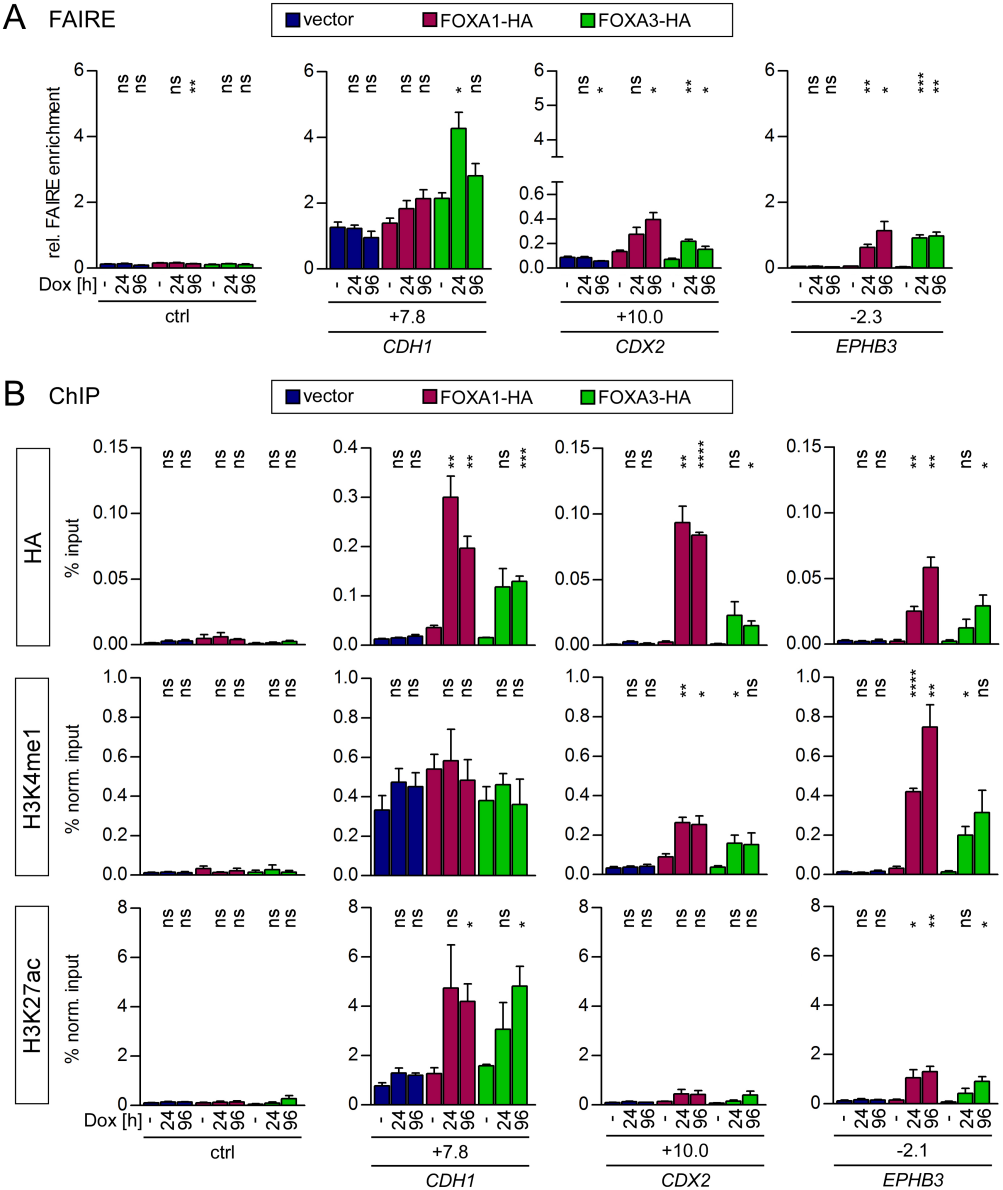
Expression of FOXA1 or FOXA3 in HCT116 cell clones leads to chromatin opening at the *CDH1*, *CDX2* and *EPHB3* enhancers and induces changes in H3K27ac/H3K4me1 deposition. (A) FAIRE analyses showing chromatin decompaction at the *CDH1*, *CDX2*, and *EPHB3* enhancers upon Dox-induced FOXA1-HA or FOXA3-HA expression in HCT116 cells. Data are given as relative (rel.) FAIRE enrichment; n = 3. As control (ctrl) the *EPHB3* locus at -7.0 kb was analyzed. (B) ChIP analyses to test for recruitment of FOXA1-HA/FOXA3-HA to the *CDH1*, the *CDX2*, and the *EPHB3* enhancers and presence of the histone modifications H3K27ac and H3K4me1 in HCT116 cells stably transduced with Dox-inducible retroviral control, FOXA1-HA or FOXA3-HA expression vectors. Data are given as percent input (HA) or as percent input normalized (norm.) to H3 (H3K27ac, H3K4me1) to compensate for variations in nucleosome density; n = 3. As control (ctrl) the *EPHB3* locus at -10.0 kb was analyzed.

Upon successful verification of Dox-inducible expression of FOXA1-HA and FOXA3-HA in HCT116 derivatives (S16 Fig) we first assessed general chromatin accessibility at the *CDH1*, *CDX2* and *EPHB3* enhancers by conducting FAIRE analyses. This revealed that the *CDH1* enhancer is in an open conformation in empty vector-transduced cells and in the absence of exogenous FOXA1-HA and FOXA3-HA when compared to a control region (Fig 9A). Still, expression of FOXA1-HA and FOXA3-HA resulted in a trend towards further chromatin opening (FOXA1-HA) and a clear induction of a more open chromatin structure at the *CDH1* enhancer (FOXA3-HA), respectively (Fig 9A). ChIP analyses revealed high levels of occupancy of the *CDH1* enhancer by ectopically expressed FOXA1-HA and FOXA3-HA (Fig 9B). This, however, did not affect the already high levels of the general enhancer mark H3K4me1 that are present under control conditions (Fig 9B). On the other hand, elevated FOXA1-HA and FOXA3-HA binding was accompanied by an increase in the abundance of the active enhancer mark H3K27ac. In line with this, we detected an upregulation of *CDH1* expression upon FOXA1-HA and FOXA3-HA induction even though *CDH1* levels were nonspecifically elevated at later time points in control cells (S16 Fig). In summary, it appears that the *CDH1* +7.8 kb enhancer adopts at least a partially active state in HCT116 cells. However, it can be further activated by overexpression of FOXA1-HA and FOXA3-HA. In contrast, we could not detect an elevated FAIRE signal for the *CDX2* and *EPHB3* enhancers when compared to a control region, suggesting that these enhancers are not accessible in HCT116 cells (Fig 9A). However, ectopic FOXA1-HA and FOXA3-HA were able to open chromatin at the condensed *CDX2* and *EPHB3* enhancers over time (Fig 9A). Both factors were recruited to the *CDX2* and *EPHB3* enhancers (Fig 9B). While this did not or only marginally increase the levels of the active enhancer histone mark H3K27ac, we observed a striking *de novo* deposition of H3K4me1 at the *CDX2* and *EPHB3* enhancers (Fig 9B). Despite these effects on their enhancer regions, expression of *CDX2* and *EPHB3* was not changed upon FOXA1-HA and FOXA3-HA induction (S16 Fig). Taken together, FOXA1-HA and FOXA3-HA were able to invade fully condensed chromatin at the *CDX2* and *EPHB3* enhancers which was accompanied by chromatin opening and the generation of a poised enhancer state. From this we conclude that FOXA1 and FOXA3 act as pioneer transcription factors at the *CDX2* and *EPHB3* enhancers.

## Discussion

During EMT, complementary gains and losses of mesenchymal and epithelial traits, respectively, are the result of extensive gene expression changes [1,5]. Central to the induction of EMT processes is a small number of transcription factors from the SNAIL, ZEB and TWIST families [1,5]. Ectopic expression of one of these factors often suffices to elicit a complete EMT [1,5,27,58,59], and SNAIL1 and ZEB1, for instance, were reported to repress key epithelial genes like *CDH1* by binding to their promoter regions [5,60,61]. Nonetheless, it is unlikely that the full extent of transcriptional reprogramming that accompanies EMT, can be explained by an immediate action of EMT inducers at the promoter regions of all relevant genes. Therefore, we examined the hypothesis that EMT inducers broadly destabilize epithelial gene expression by downregulating the FOXA family of transcription factors and consequently by widespread impairment of enhancer function.

### Control of FOXA expression by SNAIL1

Bioinformatic analyses of transcriptomes from colorectal tumors and cell lines showed that the expression of the FOXA family is anticorrelated with that of EMT-inducing transcription factors including SNAIL1 and with that of markers of mesenchymal cells in both sample sets. Anti-parallel expression of FOXA1 and SNAIL1 was experimentally confirmed in a small cohort of four CRC cell lines. These findings extend previous reports which demonstrated an inversely related expression of FOXA factors and EMT inducers, and the association of FOXA factors with the epithelial cell state in breast, lung, pancreatic, hepatocellular and prostate cancer [17–22,62]. Moreover, we observed that the expression of Snail1 in the two epithelial CRC cell lines LS174T and HT29 led to rapid downregulation of FOXA1 and occupancy of its promoter by Snail1. Thus, we identify FOXA1 as a novel and direct target gene of SNAIL1 in CRC. Likewise, SNAIL2/SLUG directly represses the FOXA1 promoter in prostate cancer cells [21]. Possibly, FOXA1 is a universal target of the SNAIL family in a wide range of cancer cell types. Interestingly, this may be a two-way relationship since repression of SNAIL2/SLUG by FOXA1 and FOXA2 has also been reported [20,21] which matches our observation that SNAIL2/SLUG expression is strongly elevated in FOXA1^KO^A2A3^lo/neg^ clones. Hence, the FOXA and SNAIL genes could provide another example of crossregulation and mutual repression in the context of EMT [63–66].

Although FOXA1, FOXA2, and FOXA3 showed the same anticorrelation with EMT inducers in global transcriptome analyses, FOXA2 and FOXA3 expression in CRC cell lines was not as strictly opposite to that of SNAIL1 when compared to FOXA1. The observed differences between FOXA expression profiles could arise from the presence or absence of regulatory factors that selectively affect individual FOXA genes independently of EMT. Overlapping but not completely identical FOXA expression patterns in the intestine suggest that gene-specific control mechanisms exist [12–14]. Nonetheless, FOXA2 and FOXA3 were also downregulated upon ectopic expression of Snail1. Therefore, it is possible that FOXA2 and FOXA3 just like FOXA1 are directly regulated by SNAIL1. Alternatively, SNAIL1 could indirectly effectuate the downregulation of FOXA2 and FOXA3, for example as a consequence of the repression of FOXA1 which is known to stimulate FOXA2 and FOXA3 gene expression [18,67]. Irrespective of their potential common control by SNAIL1 and the precise mechanism thereof, coordinated deregulation and simultaneous loss of multiple or all FOXA family members in the course of EMT may be a necessity in view of their well-known functional redundancy and the co-expression of multiple *FOXA* genes in different organs and tissues, including the small and large intestine [12–14,68].

### FOXA factors as determinants of epithelial gene expression

FOXA factors are thought to be critical determinants of the epithelial cell state and they were reported to antagonize the mesenchymal conversion of tumor cells [17–22,62]. Our finding that derivatives of an epithelial colorectal cancer cell line adopt mesenchymal morphology and gene expression upon complete loss of FOXA proteins further supports and extends these assumptions. This notwithstanding, a molecular explanation for the preeminent functional importance of FOXA factors in epithelial maintenance is lacking. In this regard, however, two distinguishing features and key properties of FOXA factors provide interesting clues. Genome-wide mapping studies of chromosomal FOXA binding sites revealed that FOXA1 and FOXA2 are predominantly or almost exclusively found at promoter-distal regions which are likely to represent transcriptional enhancers in many cases [38,69]. Enhancers vastly outnumber promoter regions and play superior roles in the spatio-temporal control of gene expression [6]. FOXA proteins in turn are of particular importance for enhancer function due to their ability to act as pioneer factors [11]. As such, FOXA factors vitally contribute to the implementation and maintenance of active enhancer states [38,69]. A function of FOXA factors at enhancer regions of epithelial genes would readily explain their key role in preserving the epithelial cell phenotype. In support of this idea we demonstrate here by a variety of gain-of-function and loss-of-function experiments that FOXA factors act upon transcriptional enhancer elements at the *CDH1*, *CDX2* and *EPHB3* genes. FOXA function is required to maintain the active chromatin structural states of these enhancers and intact FOXA binding motifs are vital for full enhancer activity. Moreover, FOXA1 and FOXA3 function as pioneer factors at the *CDX2* +10.0 kb and *EPHB3* −2.3 kb enhancer regions. FOXA factors might perform a similar function at the *CDH1* +7.8 kb enhancer but its already open chromatin state and occupancy by FOXA3 in HCT116 cells so far preclude any conclusions in this regard.

*CDH1* and *EPHB3* are essential for an intact tissue architecture of the intestinal epithelium and possess tumor suppressor activity [23,24,70]. *CDX2* plays a role in anterior-posterior patterning of the gut tube, impacts on differentiation of intestinal epithelial cells, and represents yet another tumor suppressor in colorectal carcinogenesis [25,31,71]. In a recent study we additionally described a role for FOXA1 at the -8.4 kb enhancer of the *EPHB2* gene which is closely related to *EPHB3* in terms of structure and function and which belongs to the gene signature of intestinal epithelial stem cells [23,41,70,72]. The *CDH1*, *CDX2* and the *EPHB2/B3* genes represent only a small sampling of genes but their outstanding roles in the intestinal epithelium and the involvement of FOXA in their regulation begins to shed light on the molecular underpinnings of the intimate connection between FOXA factors and the epithelial cell state.

### Gene-specific impact of FOXA pioneer factors on enhancer function and transcriptional activity and their standing in regulatory hierarchies

Experimentally interfering with FOXA activity similarly compromised structure and function of the *CDH1*, *CDX2* and *EPHB3* enhancers. Yet, there are differences between the three FOXA factors and their propensity to occupy the different enhancer regions in different cells. For instance, levels of FOXA1 occupancy are quite similar at the *CDH1*, *CDX2* and *EPHB3* enhancers in HT29 cells whereas enrichment of FOXA1 at the *EPHB3* −2.3 kb enhancer is much higher compared to the *CDX2* +10.0 kb enhancer in LS174T cells. Similarly, endogenous FOXA3 associates with the *CDH1* +7.8 kb enhancer but not with the *CDX2* +10.0 kb and *EPHB3* −2.3 kb enhancers in HCT116 cells. Furthermore, there are instances where FOXA occupancy and enhancer structural states appear to be disconnected from transcriptional activity of an associated gene. Thus, chromatin structure at the *CDH1*, *CDX2* and *EPHB3* enhancers underwent partial closure in FOXA1/FOXA2/FOXA3 triple negative cells and in the presence of dnFOXA2-HA but only *EPHB3* transcription decreased. Conversely, FOXA1 and FOXA3 occupy the *CDX*2 and *EPHB3* enhancers in SW480 and HT29 cells where expression of these genes is low or absent. We believe that these observations can be explained by the known existence of different functional states of enhancers, the multifactorial composition of enhancer complexes, and the functional cooperation among their constituents. Accordingly, FOXA proteins might function as pioneer factors and establish poised enhancer states for example at the *CDX*2 and *EPHB3* enhancers in SW480 and HT29 cells. However, additional transcription factors would be needed to stabilize the poised state and to further convert poised into active enhancers in order to stimulate promoter activity [10]. These additional factors most certainly exhibit enhancer- and cell-type-specificity. Differential activity of nuclear effectors of the Notch pathway in LS174T and HT29 cells provides an example for this and can explain the lack of *EPHB3* enhancer activity and of *EPHB3* expression in HT29 cells despite enhancer occupancy by FOXA1 and FOXA3 [50]. Differential occupancy of the *CDH1*, *CDX2* and *EPHB3* enhancers by FOXA3 in HCT116 cells may also be attributable to the absence of cooperating transcription factors specific for the *CDX2* and *EPHB3* regulatory elements even though this deficit apparently can be overcome by raising the levels of FOXA3.

A picture that emerges from these considerations, is that FOXA factors are necessary but not sufficient to ensure proper gene expression. Even so, FOXA pioneer factors function at an early and decisive step in the series of events leading to transcriptional activation. In addition, FOXA factors are involved in the regulation of multiple epithelial genes. Therefore, the repression of FOXA genes eliminates transcription factors at strategically important positions in gene-regulatory hierarchies and provides an amplifying effect which together may greatly facilitate transcriptional reprogramming during EMT.

## Materials and methods

### Cloning

For the construction of FOXA1-HA, FOXA3-HA and dominant negative (dn) FOXA2-HA expression plasmids, the respective coding regions were PCR-amplified from cDNA from LS174T cells (FOXA1), from the CCSB human ORFeome V5.1 collection (FOXA3), and from cDNA from SW403 cells (dnFOXA2). The resultant cDNA fragments were cloned into a pCS2+ vector carrying the coding region for a hemagglutinin (HA)-epitope tag. The amplified coding region of FOXA1 contains a base pair exchange representing a natural variant (G247A, SNP rs7144658), which leads to an amino acid change (A82T). This sequence variation however does not influence functional properties [73]. The sequence coding for a human dnFOXA2-HA protein was generated analogous to the previously published mouse dnFoxa2 [74]. To generate retroviral vectors, the coding regions of FOXA1-HA, FOXA3-HA and dnFOXA2-HA were transferred into the pRetroX-Tight-Pur vector (Clontech, Saint-Germain-en-Laye, France). The generation of the luciferase reporter plasmids carrying the *EPHB3* upstream region from -2684 bp to -2093 bp is described in [50]. To generate the *CDH1* and *CDX2* luciferase reporter constructs the respective regions were PCR amplified from genomic DNA from LS174T cells with the primers listed in S6 Table. The *CDH1* amplified sequence exhibits a natural base variation (G>A), representing the SNP rs1078621 (MAF/MinorAlleleCount: A = 0.4541/2274 (1000 Genomes)). To mutate the FOX binding sites, the Stratagene QuikChange site-directed mutagenesis protocol was used with the primers listed in S6 Table. The *FOXA1* luciferase reporter construct was generated as previously described [75]. All plasmids were sequence-verified.

### Cell culture, generation of stable cell lines and doxycycline treatment

LS174T, SW480, HCT116, HT29 and SW403 colorectal cancer cells were cultivated in DMEM supplemented with 10% (v/v) FCS, 10 mM HEPES, 1/100 (v/v) MEM non-essential amino acids solution and 1/100 (v/v) penicillin/streptomycin at 37°C and 5% CO_2_. Stable cell lines with Dox-inducible protein expression were generated by retroviral transduction using the pRetroX-Tight-Pur vector system. As recipient cells either LS174T and HT29 cells stably transfected with the pN1pβ-actin-rtTA2S-M2-IRES-EGFP plasmid [76] or HCT116 cells were used. In HCT116 cells, expression of a reverse tetracycline transactivator was provided through the insertion of an UbC promoter-rtTA3 cassette from the pTRIPZ vector (Open Biosystems/GE Healthcare, Freiburg, Germany) into pRetroX-Tight-Pur. Successfully infected LS174T, HT29 and HCT116 cells were selected using 1.0 and 2.0 μg/ml puromycin, respectively. Generation of LS174T and HT29 cells expressing murine Snail1-HA upon addition of Dox was described in [27]. To induce protein expression in stable cell lines, 0.1 μg/ml Dox (LS174T) and 1.0 μg/ml Dox (HCT116, HT29) was used.

### Luciferase reporter assays

For luciferase reporter assays, 1 x 10^5^ cells per well were seeded in 24-well plates. 4–6 h after seeding cells were transfected with 500 ng firefly reporter and 10 ng Renilla luciferase expression plasmids using the FuGENE6 (#E2691, Promega, Mannheim, Germany) transfection reagent. For Snail1 cotransfection experiments 5 ng Snail1-HA expression plasmid were used. After 48 h cells were harvested and lysed with lysis buffer (0.1% (v/v) NP-40; 250 mM KCl; 50 mM Tris-phosphate pH 7.8; 10% (v/v) glycerol) for at least 30 min at 4°C with gentle agitation. Lysates were cleared from cellular debris by centrifugation (16,000 x g, 10 min, 4°C). Firefly and Renilla luciferase activities were measured in duplicates using a luminometer. For calculation of the relative luciferase activity (RLA) the Firefly luciferase activity was normalized to the Renilla luciferase activity to compensate for differences in transfection efficiency.

### Immunofluorescence

For immunofluorescence stainings, 4 x 10^5^ cells were seeded in 24-well plates on round cover slips (12 mm diameter) coated with 0.2% gelatine. The following day, cells were washed with PBS and fixed with 4% paraformaldehyde for 30 min at room temperature. Thereafter, cells were washed three times with PBS, followed by permeabilization at 4°C with 0.2% Triton X-100 for 30 min. After three further washing steps with PBS cells were incubated in 5% sheep serum at 4°C for 1 h. Subsequently, cells were washed once and incubated with the primary antibodies listed in S7 Table at 4°C over night. Next, cells were washed three times with PBS, incubated with Alexa555- or Alexa488-coupled secondary antibodies and 0.3 μM DAPI at room temperature for 1 h, washed again three times and mounted using Mowiol supplemented with 10 mg/ml n-propyl gallate. Stainings were imaged using a Zeiss Axio Observer Z1 fluorescence microscope with ApoTome2 attachment and equipped with a Zeiss AxioCam. For image acquisition and initial processing the Zeiss AxioVision program SE64 was used. Upon assembly of images in one file and software-mediated enlargement of selected areas, brightness and contrast were adjusted (Canvas^™^, Canvas GFX, Inc., Fort Lauderdale, USA) whereby all panels in S6 Fig were treated in the same way.

### Formaldehyde-assisted isolation of regulatory elements

Formaldehyde-assisted isolation of regulatory elements (FAIRE) was performed as described [57] with slight modifications. Cells were crosslinked with 1% formaldehyde at room temperature for 7 min. Crosslinking was quenched by adding 125 mM glycine for 5 min. Afterwards cells were washed twice with ice-cold PBS, scraped off from the plates, spun down (850 x g, 4 min, 4°C) and washed again with ice-cold PBS. Cell lysis was performed using lysis buffer 1 (50 mM HEPES-KOH pH 7.5; 140 mM NaCl; 1 mM EDTA; 10% (v/v) glycerol; 0.5% (v/v) NP-40; 0.25% (v/v) Triton X-100) (10 min, 4°C) and lysis buffer 2 (10 mM Tris-HCl pH 8.0; 200 mM NaCl; 1 mM EDTA; 0.5 mM EGTA) (10 min, RT) with gentle agitation followed by resuspension of the cell pellet in lysis buffer 3 (10 mM Tris-HCl pH 8.0; 100 mM NaCl; 1 mM EDTA; 0.5 mM EGTA; 0.1% (w/v) sodium-deoxycholate; 0.5% (w/v) N-lauroylsarcosine). In between the lysis steps cells were spun down at 1,300 x g for 5 min at 4°C. To shear the DNA, the lysates were sonicated using the Bioruptor Plus device (Diagenode, Seraing, Belgium) with the following settings: 20–30 cycles (30 sec on/ 30 sec off) and high amplitude, yielding DNA fragments in the range of 100–750 bp. Lysates were cleared from cellular debris by centrifugation at 15,000 x g for 5 min at 4°C. To isolate the DNA, two consecutive phenol-chloroform extractions were performed followed by DNA precipitation with 0.3 M sodium acetate (pH 5.2), 20 μg glycogen and 2 volumes 95% ethanol over night at -20°C. Then, DNA was pelleted by centrifugation (15,000 x g, 30 min, 4°C), washed with ice-cold 70% ethanol, pelleted again and resuspended in 10 mM Tris-HCl pH 7.5 followed by digestion with 10 μg RNaseA (#1119915, Roche Applied Science, Mannheim, Germany) for 1 h at 37°C. To purify the DNA the QIAquick PCR Purification Kit (#28106, Qiagen, Hilden, Germany) was used according the manufacturer’s protocol. For qPCR analysis, 40 ng of precipitated DNA was used as template. Calculation of the relative FAIRE enrichment was done using the 2^-ΔCt^ method with the signals from the FAIRE samples relative to the signals from noncrosslinked control samples. Primers are listed in S6 Table.

### Chromatin immunoprecipitation

For chromatin preparation, cells were treated with 1% formaldehyde for 10 min at RT, followed by addition of 125 mM glycine for 5 min to quench crosslinking. After two washing steps with ice-cold PBS, cells were scraped off from the plates, spun down (300 x g, 5 min, 4°C) and washed one more time with PBS. For cell lysis, cells were resuspended in NP-40 buffer (0.5% (v/v) NP-40; 85 mM KCl; 5 mM HEPES pH 7.9; 1 x Complete protease inhibitors (#1697498, Roche Applied Science, Mannheim, Germany) and incubated for 10 min on ice, followed by mechanical cell disruption by applying 50 strokes with a dounce homogenizer with tight fitting pestle. Cell nuclei were pelleted by centrifugation (4,500 x g, 5 min, 4°C), resuspended in nuclei lysis buffer (50 mM Tris-HCl pH 8.1; 10 mM EDTA; 1% (w/v) SDS; 1 x Complete protease inhibitors) and incubated for 10 min on ice. To shear the chromatin to approximately 100–750 bp fragments, lysates were sonicated for 20–30 cycles (30 sec on/ 30 sec off) with high amplitude setting in a Bioruptor Plus device (Diagenode, Seraing, Belgium). Lysates were cleared from cellular debris by centrifugation (16,000 x g, 10 min, 4°C) and the chromatin concentration was measured using a NanoDrop 2000 device (Thermo Fisher Scientific, Dreieich, Germany). For immunoprecipitation, aliquots with 100–200 μg chromatin were filled up to a volume of 1 ml with sonication buffer (50 mM HEPES pH 7.9; 140 mM NaCl; 1 mM EDTA; 1% (v/v) Triton X-100; 0.1% (w/v) sodium-deoxycholate; 0.1% (w/v) SDS; 0.5 mM PMSF; 1 x Complete protease inhibitors) and mixed with antibody (listed in S7 Table) and magnetic protein G dynabeads (#100.04D, Thermo Fisher Scientific, Dreieich, Germany), preblocked with salm sperm DNA and BSA (250 μg/ml each). Immunoprecipitations were incubated over night at 4°C. Thereafter, the samples were washed with sonication buffer, wash buffer A (50 mM HEPES pH 7.9; 500 mM NaCl; 1 mM EDTA; 1% (v/v) Triton X-100; 0.1% (w/v) sodium-deoxycholate; 0.1% (w/v) SDS; 0.5 mM PMSF; 1 x Complete protease inhibitors), wash buffer B (20 mM Tris-HCl pH 8.0; 250 mM LiCl; 1 mM EDTA; 0.5% (w/v) sodium-deoxycholate; 0.5% (v/v) NP-40; 0.5 mM PMSF; 1 x Complete protease inhibitors) and TE-buffer (10 mM Tris-HCl pH 8.0; 1 mM EDTA) (each twice) for 10 min at 4°C. To elute the immunoprecipitates from the beads, two rounds of incubation in 150 μl elution buffer (50 mM Tris-HCl pH 8.0; 1 mM EDTA; 1% (w/v) SDS; 50 mM NaHCO_3_) for 10 min at 67°C with agitation (1,400 rpm) were performed. As reference samples, aliquots with 20 μg chromatin were brought to a volume of 300 μl with elution buffer and processed in the same way. The eluates were mixed with 10 μg RNaseA and 300 mM NaCl and incubated at 67°C for 5 h to reverse the DNA-protein crosslinks, followed by digestion with proteinase K at 45°C for 1 h. DNA was purified using the QIAquick PCR Purification Kit according to the manufacturer’s protocol. For qPCR analysis, 1 μl precipitated ChIP DNA and 2% input material was used as template. Primers are listed in S6 Table. Data were calculated as percent input (FOXA1, FOXA3, HA) or as percent input normalized to H3 to compensate for variation in nucleosome density (H3K27ac, H3K4me1).

### Electrophoretic mobility shift assay

Probes for EMSA were generated by PCR using biotinylated primers. Primers are listed in S6 Table. The PCR reactions were performed with Pfu Ultra II Fusion HS polymerase (#600672, Agilent Technologies, Waldbronn, Germany) according to the manufacturer’s guidelines using 10 ng DNA plasmid containing *CDH1*, *CDX2* or *EPHB3* upstream sequences as template. The protein used for EMSA was generated by *in vitro* translation using the TNT SP6 high-yield wheat germ protein expression system (#L3260, Promega, Mannheim, Germany) according to the supplier’s protocol. For the EMSA binding reaction, 10 fmol biotinylated probe, 1 μl *in vitro* translated protein, 1 μg dI:dC, 60 μg BSA, 5 mM DTT and 1x Complete protease inhibitor were mixed in EMSA buffer (20 mM HEPES pH 7.9; 75 mM NaCl; 2 mM MgCl_2_) and incubated on ice for 30 min. Samples were loaded onto a 6% polyacrylamide gel and separated by electrophoresis in TBE buffer (22.5 mM Tris; 22.5 mM boric acid; 0.5 mM EDTA) at 120 V for 3 h 15 min. Transfer of DNA::protein complexes onto a HybondN+ membrane was performed in TBE buffer at 340 mA for 1 h, followed by UV crosslink for 15 min. For the detection of DNA::protein complexes the Chemiluminescent Nucleic Acid Detection Module kit (#89880, Thermo Fisher Scientific, Dreieich, Germany) was used according the manufacturer’s instructions followed by exposure of light-sensitive films.

### Western Blotting and immunodetection

For detection of FOXA1, FOXA1/A2, FOXA3 and Snail1 protein levels in CRC cells, nuclear extracts were used. Expression of all other proteins was analyzed using whole cell lysates. Protein isolation was performed as described [50]. The antibodies used are listed in S7 Table. Antibody::antigen complexes were detected and visualized as described [77].

### RNA isolation, cDNA synthesis and quantitative reverse transcription polymerase chain reaction (qRT-PCR)

For RNA isolation the PeqGOLD total RNA kit (#732–2871, Peqlab/VWR Life Science, Bruchsal, Germany) was used. The cDNA synthesis was done with the SuperScript II Reverse Transcriptase (RT) kit (#18064014, Thermo Fisher Scientific, Dreieich, Germany) according the manufacturer’s protocol with the following modifications: 500–2000 ng total RNA, 5 μM oligo-dT primer and 50 units of SuperScript II RT were used. qRT-PCR was performed with the CFX96 multicolor realtime PCR detection systems (Bio-Rad Laboratories, Munich, Germany). For the PCR reaction, KAPA SYBR green reaction mix (#KAPBKK4606-305, Peqlab/VWR Life Science, Bruchsal, Germany) was mixed with 0.2 μM primer and a cDNA amount equivalent to 20 ng total RNA. For gene expression analyses, data were normalized to *GAPDH* expression and calculated using the 2^-ΔCt^ method. Primers are listed in S6 Table.

### CRISPR/Cas9 genome editing

Design of the gRNAs targeting different transcription factor binding sites in the *EPHB3* enhancer and for the FOXA1 knockout was done with the CRISPR design tool developed by the Zhang lab (http://crispr.mit.edu) [78]. The sequences of the different gRNAs targeting the *EPHB3* enhancer are the following: FOX binding sites 5’-CTGGCTGGCTGTGTTTGTCCAGG-3’; TCF binding site 5’-GCACCAAGCTCCTTTGTTCTTGG-3’ and RBPJ binding site 5’- TCTCCAGGAAGCATTCCCGTGGG-3’. For the generation of a FOXA1 knockout the gRNA with the sequence 5’- CATGTTGCCGCTCGTAGTCATGG-3’ was used. Cloning of the gRNAs into the gRNA cloning vector (#41824, addgene, Cambridge, USA) was performed as described in the gRNA synthesis protocol (https://www.addgene.org/static/cms/filer_public/13/af/13af84f5-50ee-472e-96f1-35c5b0533645/hcrispr-grna-synthesis.pdf) [79]. For Cas9-GFP expression the plasmid #44719 (addgene, Cambridge, USA) was used. For construction of the donor plasmid the DNA region from -2285 bp to -1493 bp of the *EPHB3* locus was PCR amplified from genomic DNA from LS174T cells and transferred into a pUC18 vector. Mutagenesis of the transcription factor binding sites was done according to the QuikChange site-directed mutagenesis protocol with the primers listed in S6 Table. All plasmids were sequence-verified. For genome editing in LS174T cells, 2 x 10^6^ cells were transfected with 0.5 μg Cas9-GFP, 0.5 μg gRNA, 0.5 μg dsRed expression vectors and, in the case of targeting transcription factor binding sites at the *EPHB3* enhancer, 2.5 μg donor DNA plasmid by nucleofection using the Cell Line Nucleofector kit L (#VCA-1005, Lonza, Cologne, Germany). 72 h post nucleofection, GFP+/RFP+ cells were single cell sorted. After approximately 3 weeks cells were used for analyses.

### Identification of successfully genome-edited cell clones

To identify genome-edited cell clones carrying mutations in the transcription factor binding sites in the *EPHB3* enhancer, genomic DNA from LS174T single cell clones and LS174T wild-type cells was isolated using the DNeasy Blood & Tissue kit (#69506, Qiagen, Hilden, Germany) according to the manufacturer’s instructions. The genomic DNA was used to amplify regions of the *EPHB3* locus spanning the CRISPR/Cas9 cutting site by semiquantitative polymerase chain reaction using the primers listed in S6 Table. To test for alterations in the DNA sequence induced by genome-editing, a Surveyor assay was performed according to the instruction manual (#706025, Integrated DNA Technologies, Coralville, USA). Briefly, PCR products of potentially genome-edited cell clones either alone or mixed with PCR products from LS174T wild-type cells were heat-denatured and rehybridized followed by digestion with the Surveyor Nuclease. Genome-edited LS174T cell clones were identified by a specific cleavage pattern upon Surveyor Nuclease digest. In order to prove repair by homologous recombination and integration of donor DNA into the genome, a restriction enzyme digest of the PCR products was performed identifying the specific mutations introduced by the donor DNA. All mutations were sequence-verified. LS174T FOXA1 knockout cell clones were identified by Western Blot analyses.

### Transcriptome analysis

Total RNA was extracted in biological triplicates from untreated parental LS174T cells as well as the FOXA1^WT^ clone 1C6, and the FOXA1^KO^A2A3^lo/neg^ clones 2F8 and 2F12. Biotinylated cRNA was prepared with the Ambion MessageAmp kit for Illumina arrays according to the manufacturer’s protocol. Quality of cRNA was controlled using RNA Nano Chip Assay on an Agilent 2100 and hybridized to Illumina HumanHT12-v4 BeadChips (Illumina, San Diego, CA, USA) according to the manufacturer’s protocol. Raw microarray data were chip-wise processed using the Bioconductor R package beadarray [80] and subsequently quantile normalized together. Illumina Probes were mapped to Entrez IDs using the Illumina Human v4 annotation data (Version 1.26) from Bioconductor. If several probes mapped to the same Entrez ID, the one having the largest interquartile range was retained. Microarray data were deposited in GEO under the access ID GSE106073. Differential gene expression analysis between treatment groups was calculated using the R limma package [81]. P-values were corrected for multiple testing using Benjamini & Hochberg. Gene set enrichment analyses were performed with GAGE algorithm [82], which tests whether a gene set is significantly perturbed relative to all genes. For functional annotation, we used pathways from KEGG and REACTOME as provided by the CP:KEGG and CP:REACTOME gene set collection from the Molecular Signatures Database (MSigDB, v. 6.1) [83]. Only gene sets having more than 5 and less than 500 genes were considered in order to retain those that are statistically robust and biologically informative.

### ChIP-Seq data analysis

We reanalyzed six ChIP-Seq data sets from the GEO database for FOXA1 binding in T47D, HepG2 and A549 cells (GSM803409, GSM803432, GSM1010725) and for FOXA2 binding in Caco2, HepG2 and A549 cells (GSE66218, GSM803403 and GSM1010724). Individual fastq files were trimmed using trimmomatic and aligned to the Human Genome version 19 (hg19) by bwa-mem [84,85]. The resulting bam files were merged in case of replicates and subjected to peak calling by MACS2 [37] using default parameters and a false-discovery rate corrected p-value < 0.01. Peak locations were further annotated according to the known genes in hg19 using the R/Bioconductor package ChIPseeker [86]. The annotation was lumped into seven features: Promoter region (3 kb upstream and downstream of a transcription start site), exonic, intronic, 5’-region, 3’-region, downstream (> 3 kb) and distal intergenic. ChIP-seq peaks from different samples were counted as overlapping if they were located less than 1000 bp apart. The functional annotation of the chromatin states was derived from a 15-state multivariate HMM that is based on five histone marks (H3K4me1, H3K4me3, H3K36me3, H3K27me3, H3K9me3) and 127 epigenomes from the epigenome roadmap consortium [39,40]. The genome was discretized into 200 bp bins each for which the most likely chromatin state was assigned according to the posterior probability of the trained HMM. The FOXA1 and FOXA2 ChIP-seq data were annotated according to the label of the bins coinciding with the respective peak summits. Genome browser views of chromatin states, gene loci and ChIP-seq peaks were generated using the WashU EpiGenome Browser (http://epigenomegateway.wustl.edu/).

### Pairwise correlation analyses and processing of microarray gene expression data

Pairwise correlation analyses and calculation of relative gene expression levels based on microarray data from CRC tumors and cell lines were performed as described [50,41]. The genes included in the studies were the mesenchymal marker genes *VIM* (VIMENTIN), *FN1* (FIBRONECTIN) and *CDH2* (N-CADHERIN), the EMT inducers *SNAI1* (SNAIL1), *SNAI2* (SNAIL2), *ZEB1*, *ZEB2*, *TWIST1* and *TWIST2*, the epithelial marker gene *CDH1* (E-CADHERIN), the EPHB receptors *EPHB2* and *EPHB3*, the intestine-specific differentiation markers *CDX1* and *CDX2*, and the tight junction proteins *OCLN* (OCCLUDIN), *TJP1*, *TJP2*, *TJP3*, and *CLDN* (CLAUDIN) *1*, *2*, *3*, *4*, *5*, *7*, *8*, *12*, *15* that are expressed in the human intestine [87].

### Statistical analysis

Statistical analysis was performed using the unpaired, two-tailed Student’s *t*-test. Unless otherwise stated, the comparison was between Dox-treated versus untreated cells and between parental versus genome-edited cells. Significant changes are shown by the respective p-values represented with *: p < 0.05; **: p < 0.01; ***: p < 0.001. Non-significant changes are denoted with ns.

## Supporting information

S1 Fig Expression of FOXA family members clusters with epithelial markers and is inversely correlated with that of mesenchymal genes. (A, B) Unsupervised hierarchical clustering of relative gene expression levels from 290 primary CRCs (GSE14333) (A) and 151 CRC cell lines (GSE59857) (B). Expression of FOXA family members in relation to that of genes characteristic for mesenchymal cells (*FN1*, *SNAI1*, *SNAI2*, *TWIST*, *VIM*, *ZEB1*) and epithelial cells (*CDH1*, *CDX2*, *CLDN3*, *EPHB3*) was analyzed. Color scale represents relative expression levels as depicted by the bars on the left side of the panels.
(TIF)

S2 Fig Crossreactivity of FOXA antibodies. Western Blot analyses to test the specificity of the FOXA antibodies. Immunodetection of the HA-tag served to verify equal protein amounts of the *in vitro* translated FOXA proteins. Immunodetection of FOXA1 (A), FOXA2 (B) and FOXA3 (C) revealed a marginal crossreactivity of the FOXA1 antibody with FOXA2 and a considerable crossreactivity of the FOXA2 antibody with FOXA1. The FOXA3 antibody showed no signs of crossreactivity with FOXA1 or FOXA2 proteins.
(TIF)

S3 Fig Snail1 expression interferes with FOXA expression in HT29 cells. (A) qRT-PCR analyses to assess *Snail1*, *FOXA1*, *FOXA2*, and *FOXA3* relative expression (rel. expr.) levels in HT29 cells stably transduced with Dox-inducible retroviral control or Snail1-HA expression vectors. Shown is the mean and SEM; n = 3. (B) Western Blot to analyze Snail1-HA, FOXA1, FOXA1/2, and FOXA3 protein levels upon Dox-induced Snail1-HA expression in HT29 cells. M_W_ = molecular weight in kDa. To monitor equal protein loading RNA polymerase II (RNAPII) was detected. (C) ChIP analysis to test for Snail1-HA occupancy at the *FOXA1* promoter in HT29 cells. Data were calculated as percent input. Shown are the mean and SEM; n = 4.
(TIF)

S4 Fig Snail1 represses *FOXA1* promoter activity in LS174T and HT29 cells. (A, B) Luciferase reporter assay in LS174T (A) and HT29 (B) cells with constructs harboring the *FOXA1* promoter. Mutations of the respective E-boxes are indicated by red crosses. E-box I apparently has a dual function. It is involved in activation of the FOXA1 promoter in the absence of Snail1-HA. Additionally, E-box I in part mediates the repressive effect of Snail1-HA. Shown is the mean and SEM; n≥3. RLA: relative luciferase activity. Statistical significance was calculated between samples without and with Snail1 expression.
(TIF)

S5 Fig Expression analyses of *FOXA* genes, *CDH1*, *CLDN3*, *SNAI2*, *LEF1* and *CDH11* in LS174T cells with wild-type, low, and absent FOXA expression. Expression of *FOXA1*, *FOXA2*, *FOXA3*, *CDH1*, *CLDN3*, *SNAI2*, *LEF1* and *CDH11* in parental LS174T cells and cell clones subjected to CRISPR/Cas9-mediated genome editing of the FOXA1 locus was assessed by qRT-PCR analyses. rel. expr.: relative expression. Data are shown as mean and SEM; n = 3.
(TIF)

S6 Fig Expression and cellular localization of LEF1, CADHERIN11, E-CADHERIN and CLAUDIN3 in LS174T cells with wild-type, low, and absent FOXA expression. Expression of LEF1, CADHERIN11, E-CADHERIN and CLAUDIN3 in parental LS174T cells and cell clones subjected to CRISPR/Cas9-mediated genome editing of the FOXA1 locus was assessed by immunofluorescence studies. Areas within yellow frames were enlarged fivefold and are presented on the right. Scale bars: 50 μm and 10 μm (fivefold enlargements).
(TIF)

S7 Fig Significantly higher numbers of FOXA1/FOXA2 binding sites are associated with epithelial gene loci. (A) Total numbers and genic distribution of FOXA1/FOXA2 ChIP-seq peaks in different cell lines. The p-values refer to the results of bootstrapping analyses (exemplary results for this analyses are shown in panel B) to test whether the number of FOXA1/FOXA2 ChIP-seq peaks at epithelial and mesenchymal genes is significantly different from random groups of genes. (B) FOXA1 ChIP-seq data from HepG2 cells were analyzed by a bootstrapping approach to estimate whether the number of binding regions at epithelial genes is significantly high or low. Out of all 22,000 annotated genes random groups of N = 45 or N = 54 genes representing the sample size of epithelial and mesenchymal gene groups, respectively, were selected, and the numbers of associated peaks were counted. The resulting distribution of associated peak numbers from 10,000 trials is shown. The red lines indicate the number of associated peaks for the epithelial and mesenchymal gene groups. The p-values shown were calculated from fitting a skewed normal distribution to the histogram.
(TIF)

S8 Fig FOXA1/FOXA2 ChIP-seq peaks colocalize more frequently with intergenic enhancer regions at epithelial genes. (A) Genome browser view of the 15-state chromatin model in relation to gene structure and FOXA1/FOXA2 binding regions for mesenchymal (*CDH2*, *FN1*) and epithelial (*CDH1*, *TJP2*) signature genes. Localization and width of FOXA1/FOXA2 ChIP-seq peaks are represented by the differently sized bars at the bottom of the scheme. Vertical white lines were added to facilitate aligning ChIP-seq peaks and chromatin states. (B) Box-whisker plots depicting the pileup values from the MACS output for the transcription factors FOXA1 and FOXA2 for the two cell lines A549 and HepG2. The boxplots indicate median values of the population and the boxes indicate the interquartile range (IQR) from the first to the third quantile of the pileup distribution. The notches indicate the +/-1.58 IQR/sqrt(n), where n denotes the number of respective peaks. The whiskers indicate the 1.5 IQR. sqrt: square root.
(TIF)

S9 Fig DNA sequence conservation of FOXA motifs across several species. Alignment of the DNA sequences of the FOXA motifs in the *CDH1* +7.8 kb (A), the *CDX2* +10.0 kb (B) and the *EPHB3* −2.3 kb (C) ECRs. The consensus of the FOXA motifs is highlighted by a grey box. Sequence identity is indicated by asterisks. The indicated base positions are relative to the transcriptional start site of the human DNA sequence based on the Ensembl genome browser.
(TIF)

S10 Fig Expression levels of *CDH1*, *CDX2*, and *EPHB3* in four CRC cell lines. (A-C) qRT-PCR to analyze expression of *CDH1* (A), *CDX2* (B), and *EPHB3* (C) in the indicated CRC cell lines. Data are shown as mean and SEM; n = 3.
(TIF)

S11 Fig Reduced FOXA1 binding to their enhancer regions is paralleled by downregulation of *CDH1*, *CDX2* and *EPHB3* in Snail1-HA-expressing cells. (A) ChIP analyses to test for binding of FOXA1 to the *CDH1*, the *CDX2*, and the *EPHB3* enhancers in LS174T cells stably transduced with Dox-inducible retroviral control or Snail1-HA expression vectors. Data are given as percent input; n = 3. As control (ctrl) the *EPHB3* locus at -10.0 kb was analyzed. (B) qRT-PCR to analyze expression of *Snail1-HA*, *CDH1*, *CDX2*, and *EPHB3* in LS174T cells stably transduced with Dox-inducible retroviral control or Snail1-HA expression vectors. Data are shown as mean and SEM; n = 2. (C) Western Blot to analyze CDX2, E-CADHERIN (E-CAD), and EPHB3 protein levels in nuclear extracts (CDX2; left panel) and whole cell extracts (E-CADHERIN, EPHB3; right panel), respectively, upon Dox-induced Snail1-HA expression in LS174T cells. To monitor equal protein loading RNA polymerase II (RNAPII) and α-TUBULIN (α-TUB) was detected. M_W_ = molecular weight in kDa.
(TIF)

S12 Fig Occupancy of the *CDH1*, *CDX2*, and *EPHB3* enhancers by dnFOXA2-HA. (A) EMSA to demonstrate binding of dnFOXA2-HA to the FOX binding motifs at the *EPHB3* −2.3 kb enhancer. Asterisks: non-specific bands. mut: mutated. (B) ChIP analyses using anti-HA antibodies showing recruitment of dnFOXA2-HA to the *CDH1* +7.8 kb, the *CDX2* +10.0 kb and the *EPHB3* −2.3 kb enhancers upon Dox treatment in LS174T cells stably transduced with Dox-inducible dnFOXA2-HA expression vectors. As control (ctrl) the *EPHB3* locus at -10.0 kb was analyzed. Data are given as percent input. Shown is the mean and SEM; n≥4.
(TIF)

S13 Fig Mutation of transcription factor binding sites at the *EPHB3* enhancer by genome editing. (A) Scheme of the strategy to mutate transcription factor binding sites at the *EPHB3* enhancer exemplified by targeting the FOX binding motifs. The structure of the *EPHB3* enhancer with the FOX binding sites is schematically shown at the bottom. The blue line denotes the position of the guide RNA. The structure of the donor plasmid is shown in the upper part. The left and right homology arms (approximately 600 and 750 bp, respectively) are schematically indicated by grey trapezes. (B) CRISPR/Cas9-mediated mutation of the FOX, TCF and RBPJ binding sites within the *EPHB3* enhancer. A part of the DNA sequence of the *EPHB3* enhancer with the FOX binding sites (upper), the TCF binding site (middle) and RBPJ binding site (lower) is given. The sequence of the transcription factor binding sites is written in bold. The sequence targeted by the guide RNA is highlighted by light blue shading. The PAM is indicated by the red line. The cutting site of the Cas9 nuclease is marked by the arrowhead. The mutations introduced in the transcription factor binding sites by integration of the donor plasmid are shown in lowercase. The recognition sequence of the restrictions enzymes (written in green) used to test for successful integration of the donor plasmid is indicated in the DNA sequence by the green line. (C) Scheme of the *EPHB3* enhancer and its known transcription factor binding sites. The positions of the EMSA probes P1 and P2 are shown in red and blue, respectively. (D, E) EMSA showing that the mutations introduced into the genomic DNA of LS174T cells interfere with binding of RBPJ (D), the TCF factor TCF7L2 (E) and the FOXA protein FOXA1 (E). Arrows mark the protein::DNA complexes. (F, G) Western Blot analyses to control the expression of the *in vitro* translated proteins used for EMSA shown in (D, E). M_W_ = molecular weight in kDa.
(TIF)

S14 Fig Identification of LS174T cell clones with biallelic mutations in transcription factor binding sites at the *EPHB3* enhancer. (A) Surveyor assay to test for genome alterations in LS174T cell clones subjected to CRISPR/Cas9 genome editing to mutate the FOX binding sites (left panel), the TCF binding site (middle panel), and the RBPJ binding site (right panel). Changes in the genomic DNA of cell clones were detected by cleavage of a mixture of PCR products from wild-type and cell clone DNA. The specific cleavage pattern is indicated by the arrowhead. To test for homozygous DNA alterations, PCR products of LS174T cell clones were subjected to Surveyor nuclease digest without admixture of wild-type PCR products. Asterisks: non-specific PCR products. (B) Restriction enzyme digest to test for successful integration of the donor plasmid and thus mutation of the FOX binding sites (left panel), the TCF binding site (middle panel) and the RBPJ binding site (right panel) in LS174T cell clones subjected to CRISPR/Cas9 genome editing. Changes in the cleavage pattern indicative of successful binding site mutations are shown by the arrowheads. Asterisks: non-specific PCR products. (C) Electropherograms of the sequencing analyses of PCR products from LS174T cell clones subjected to CRISPR/Cas9 genome editing showing mutations of the FOX binding sites (upper panel), the TCF binding site (lower left panel) and the RBPJ binding site (lower right panel) in the respective cell clones. The DNA sequences with the transcription factor motifs (written in bold) are given above the electropherograms. The bases that are changed compared to the wild-type sequence are written lowercase. The sequences of the mutated transcription factor binding sites are underlined in red in the electropherogram.
(TIF)

S15 Fig Genome-edited LS174T cell clones do not show phenotypically relevant differences in FOXA expression and activity of Notch and Wnt/β-Catenin signaling. (A) qRT-PCR analyses to assess *FOXA1*, *FOXA2*, and *FOXA3* relative expression (rel. expr.) levels in LS174T cell clones subjected to CRISPR/Cas9 genome editing. Data are shown as mean and SEM; n = 3. Statistical significance was calculated between the respective cell clone and the LS174T wild-type cell pool. (B) Western Blot to analyze abundance of the FOXA proteins FOXA1, FOXA1/2, and FOXA3 (left panel), NICD (indicative of active Notch signaling), and β-CATENIN (β-CAT) and TCF7L2 (mediators of Wnt/β-Catenin signaling) (right panel) in LS174T cell clones subjected to CRISPR/Cas9 genome editing. M_W_ = molecular weight in kDa. α-TUBULIN (α-TUB), GSK3β and RNA polymerase II (RNAPII) immunodetections served as loading controls. (C) qRT-PCR analyses to test for expression levels of the FOXA1/2 target gene *MUC2*, the Notch target gene *NRARP* and the Wnt/β-Catenin target gene *AXIN2* in LS174T cell clones subjected to CRISPR/Cas9 genome editing. Shown are the mean and SEM; n = 3. Statistical significance was calculated between the respective cell clone and the LS174T wild-type cell pool.
(TIF)

S16 Fig Differential impact of FOXA1 and FOXA3 on *CDH1*, *CDX2* and *EPHB3* expression. (A) qRT-PCR analyses to assess *FOXA1*, *FOXA3*, *CDH1*, *CDX2*, and *EPHB3* relative expression (rel. expr.) levels in HCT116 cells stably transduced with Dox-inducible retroviral control, FOXA1-HA or FOXA3-HA expression vectors. Data are shown as mean and SEM; n = 3. (B) Western Blot to analyze FOXA1-HA, FOXA3-HA, E-CADHERIN, CDX2 and EPHB3 protein expression in HCT116 cells stably transduced with Dox-inducible retroviral control, FOXA1-HA or FOXA3-HA expression vectors. M_W_ = molecular weight in kDa. GSK3β and α-TUBULIN (α-TUB) immunodetections served as loading controls.
(TIF)

S1 Table Correlations between clinical parameters and expression levels of cluster 1 and cluster 2 genes. (DOCX)

S2 Table Compilation and nomenclature of genome-edited LS174T cell clones used in the study. (DOCX)

S3 Table List of genes showing significantly different expression (adj. p-value < 0.05) in FOXA1 knockout clones versus parental/wild-type cells. (XLSX)

S4 Table List of KEGG/Reactome pathways with significant (adj. p-value < 0.05) overall up- and downregulation in FOXA1 knockout clones versus parental/wild-type cells. (XLSX)

S5 Table List of EMT-associated epithelial and mesenchymal genes. (DOCX)

S6 Table Sequences of oligonucleotides used in the study. (DOCX)

S7 Table Antibodies used for immunoblotting, immunofluorescence and ChIP. (DOCX)
