# Supplementary figures and images for: SNAIL1-mediated downregulation of FOXA proteins facilitates the inactivation of transcriptional enhancer elements at key epithelial genes in colorectal cancer cells
Source: PLoS Genet. 2017 Nov 20;13(11):e1007109. doi: 10.1371/journal.pgen.1007109 (PMC5714381; doi:10.1371/journal.pgen.1007109)

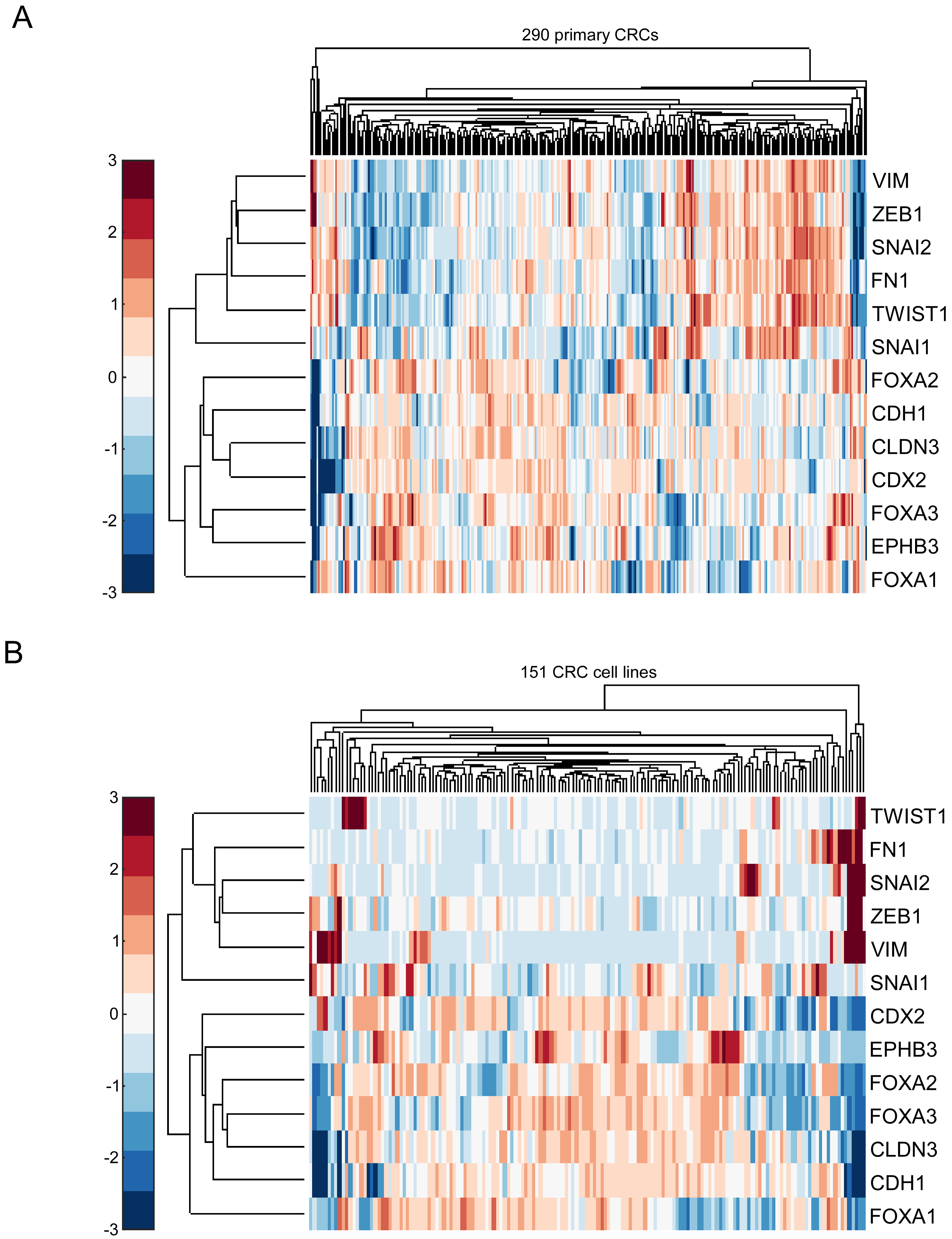

Supplement: S1 Fig — (A, B) Unsupervised hierarchical clustering of relative gene expression levels from 290 primary CRCs (GSE14333) (A) and 151 CRC cell lines (GSE59857) (B). Expression of FOXA family members in relation to that of genes characteristic for mesenchymal cells (FN1, SNAI1, SNAI2, TWIST, VIM, ZEB1) and epithelial cells (CDH1, CDX2, CLDN3, EPHB3) was analyzed. Color scale represents relative expression levels as depicted by the bars on the left side of the panels. (TIF) [file pgen.1007109.s001.tif]

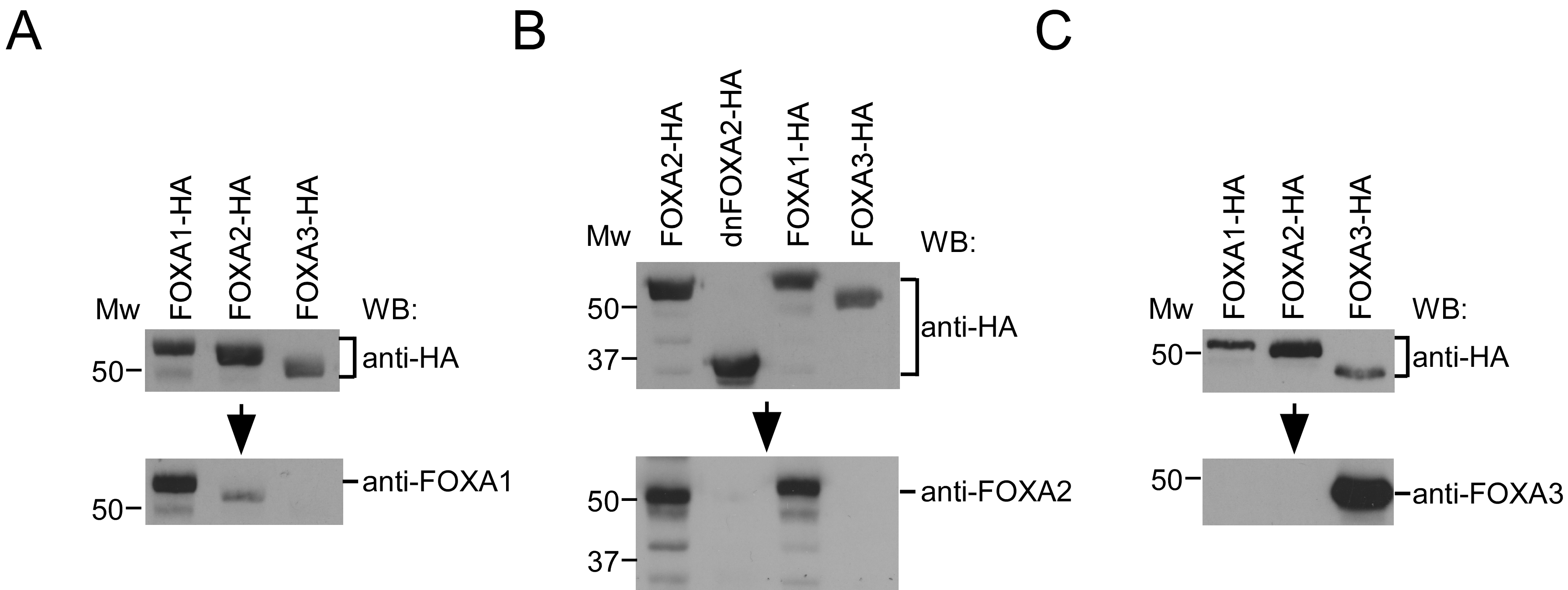

Supplement: S2 Fig — Western Blot analyses to test the specificity of the FOXA antibodies. Immunodetection of the HA-tag served to verify equal protein amounts of the in vitro translated FOXA proteins. Immunodetection of FOXA1 (A), FOXA2 (B) and FOXA3 (C) revealed a marginal crossreactivity of the FOXA1 antibody with FOXA2 and a considerable crossreactivity of the FOXA2 antibody with FOXA1. The FOXA3 antibody showed no signs of crossreactivity with FOXA1 or FOXA2 proteins. (TIF) [file pgen.1007109.s002.tif]

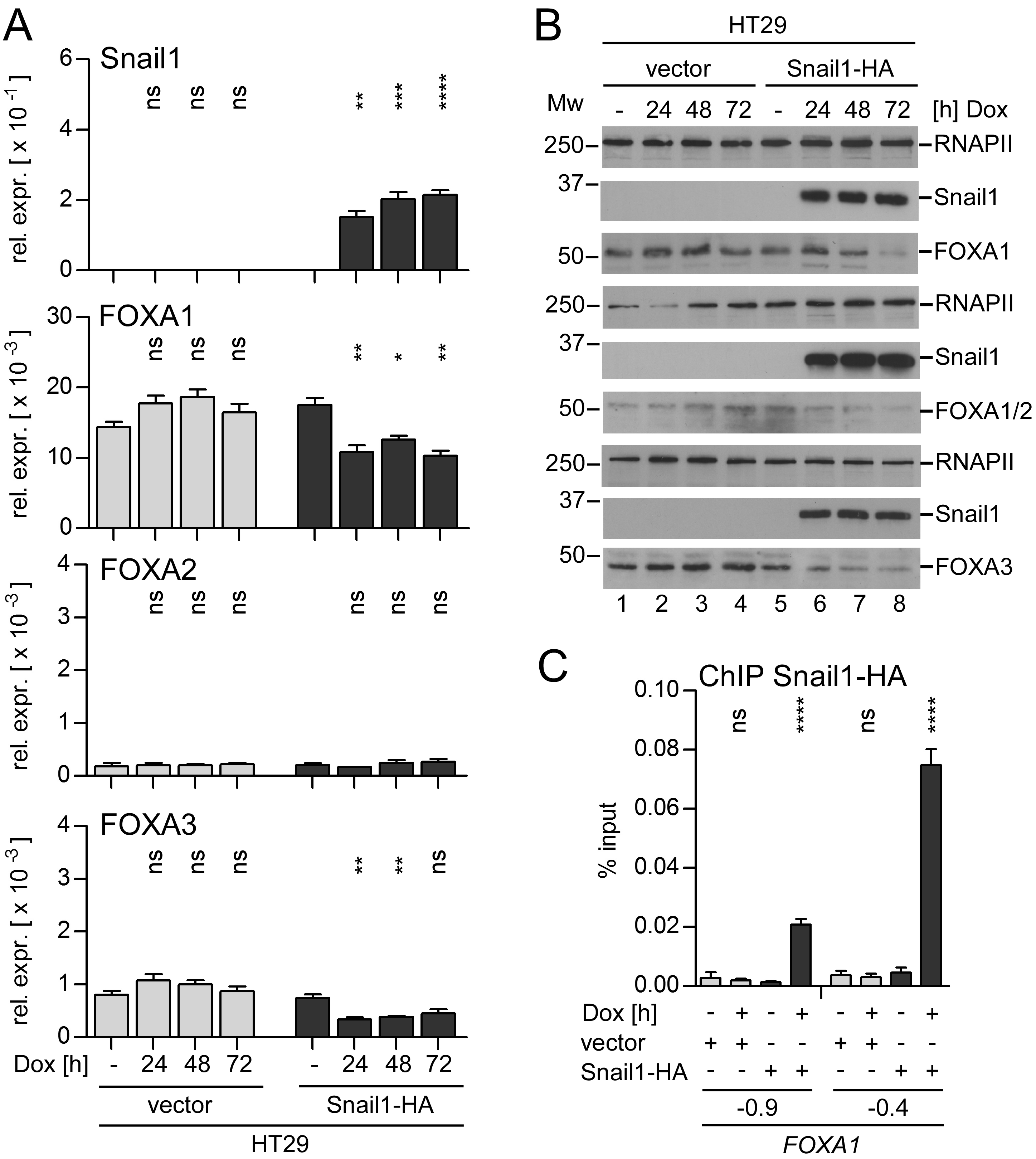

Supplement: S3 Fig — (A) qRT-PCR analyses to assess Snail1, FOXA1, FOXA2, and FOXA3 relative expression (rel. expr.) levels in HT29 cells stably transduced with Dox-inducible retroviral control or Snail1-HA expression vectors. Shown is the mean and SEM; n = 3. (B) Western Blot to analyze Snail1-HA, FOXA1, FOXA1/2, and FOXA3 protein levels upon Dox-induced Snail1-HA expression in HT29 cells. MW = molecular weight in kDa. To monitor equal protein loading RNA polymerase II (RNAPII) was detected. (C) ChIP analysis to test for Snail1-HA occupancy at the FOXA1 promoter in HT29 cells. Data were calculated as percent input. Shown are the mean and SEM; n = 4. (TIF) [file pgen.1007109.s003.tif]

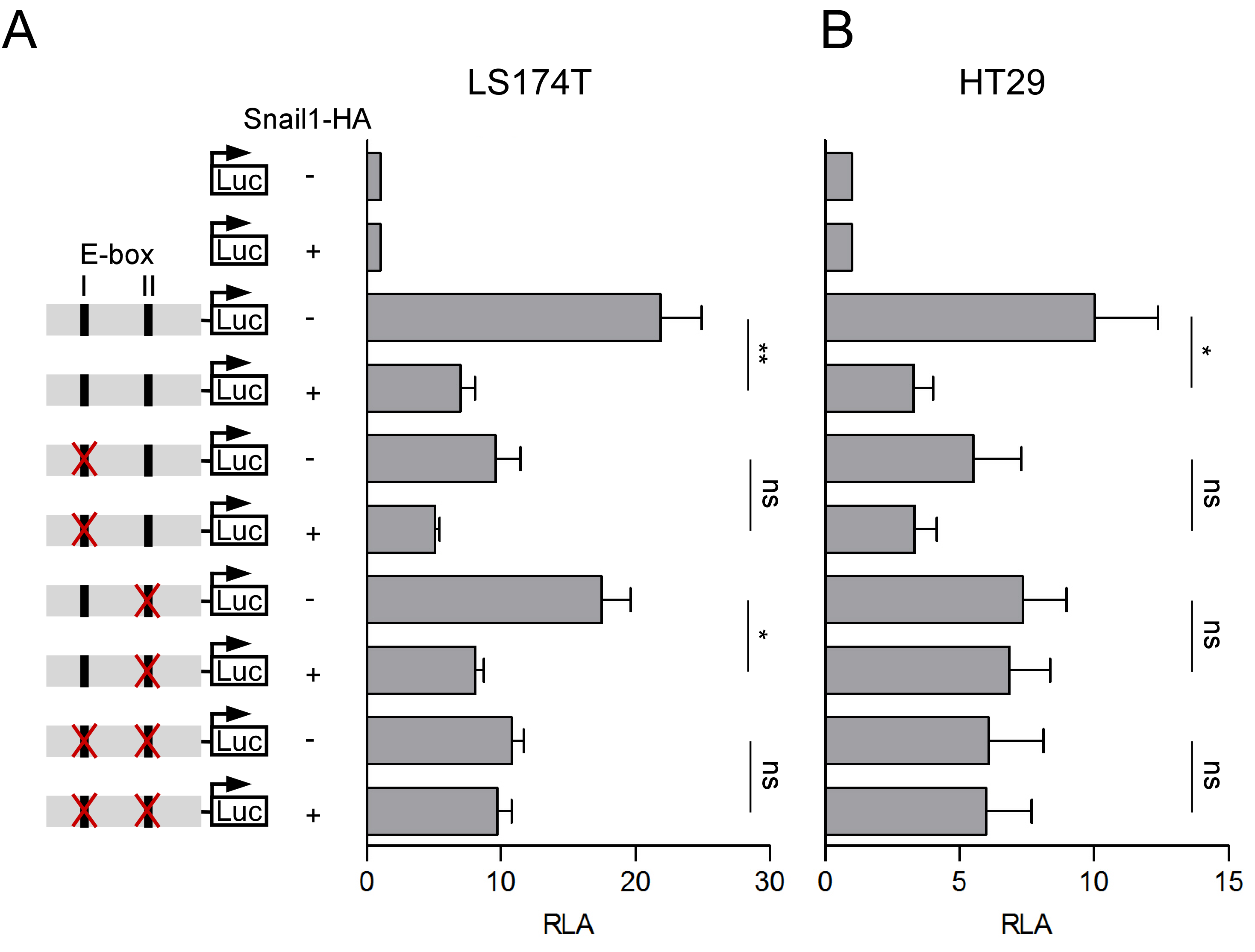

Supplement: S4 Fig — (A, B) Luciferase reporter assay in LS174T (A) and HT29 (B) cells with constructs harboring the FOXA1 promoter. Mutations of the respective E-boxes are indicated by red crosses. E-box I apparently has a dual function. It is involved in activation of the FOXA1 promoter in the absence of Snail1-HA. Additionally, E-box I in part mediates the repressive effect of Snail1-HA. Shown is the mean and SEM; n≥3. RLA: relative luciferase activity. Statistical significance was calculated between samples without and with Snail1 expression. (TIF) [file pgen.1007109.s004.tif]

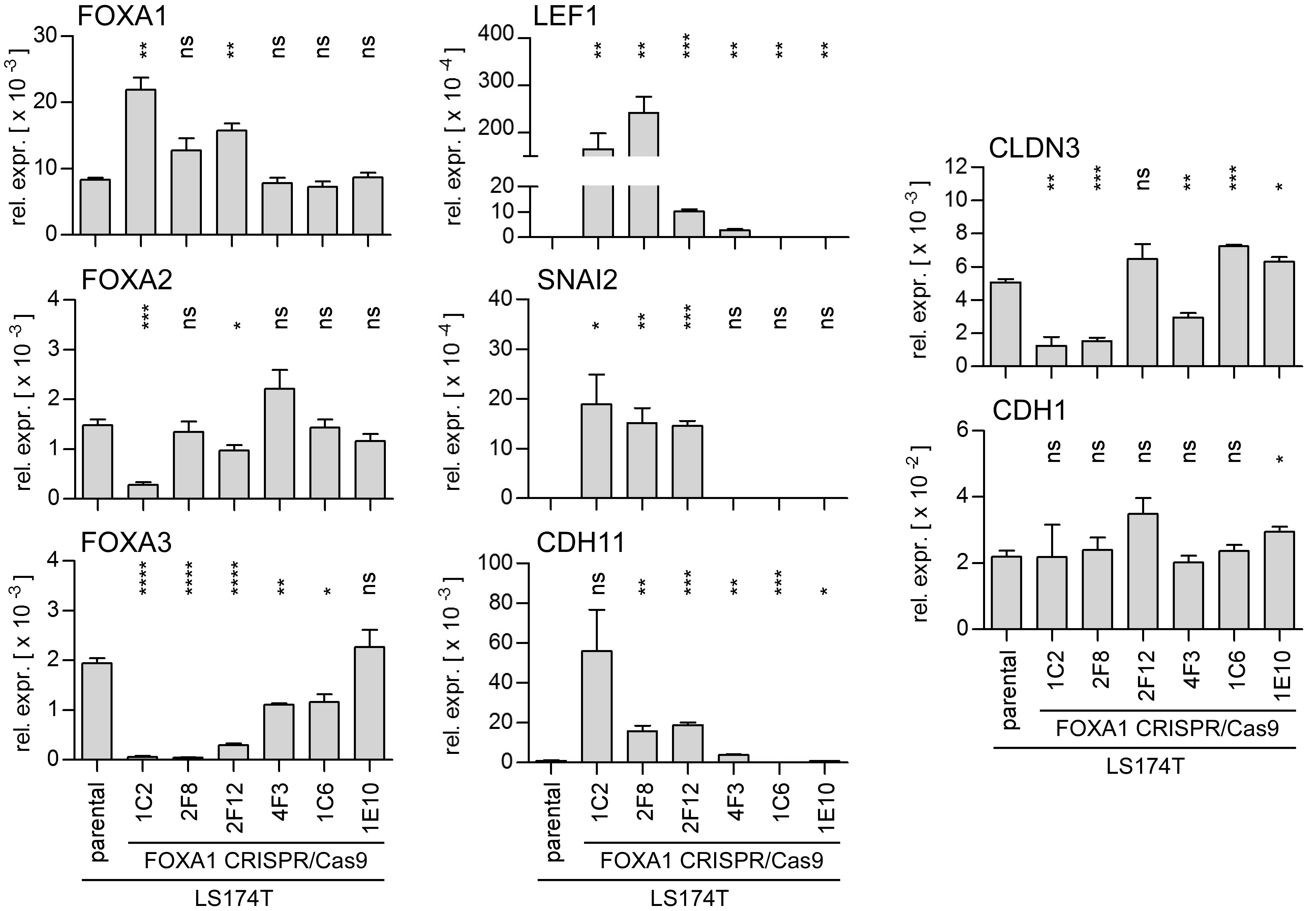

Supplement: S5 Fig — Expression of FOXA1, FOXA2, FOXA3, CDH1, CLDN3, SNAI2, LEF1 and CDH11 in parental LS174T cells and cell clones subjected to CRISPR/Cas9-mediated genome editing of the FOXA1 locus was assessed by qRT-PCR analyses. rel. expr.: relative expression. Data are shown as mean and SEM; n = 3. (TIF) [file pgen.1007109.s005.tif]

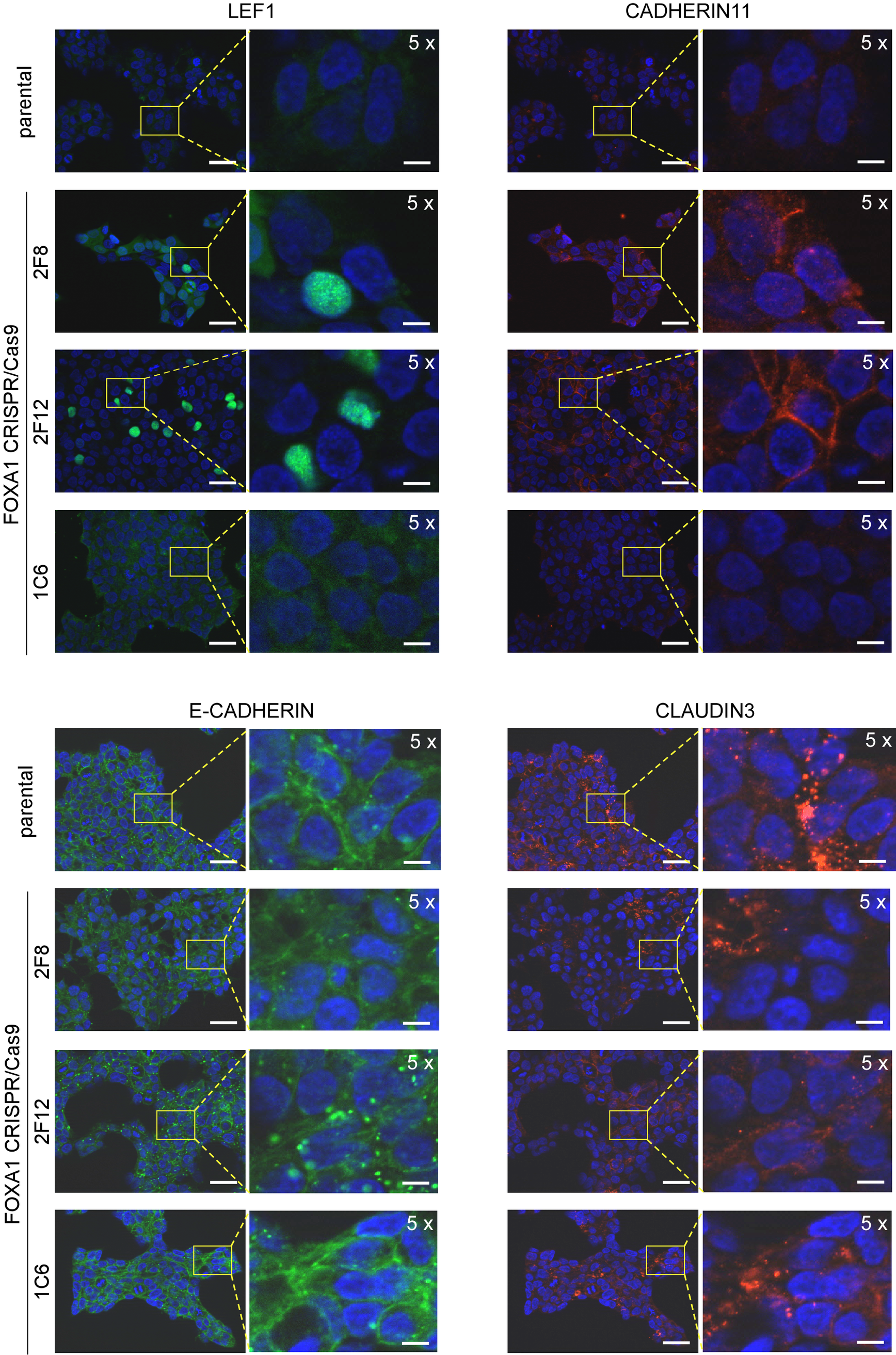

Supplement: S6 Fig — Expression of LEF1, CADHERIN11, E-CADHERIN and CLAUDIN3 in parental LS174T cells and cell clones subjected to CRISPR/Cas9-mediated genome editing of the FOXA1 locus was assessed by immunofluorescence studies. Areas within yellow frames were enlarged fivefold and are presented on the right. Scale bars: 50 μm and 10 μm (fivefold enlargements). (TIF) [file pgen.1007109.s006.tif]

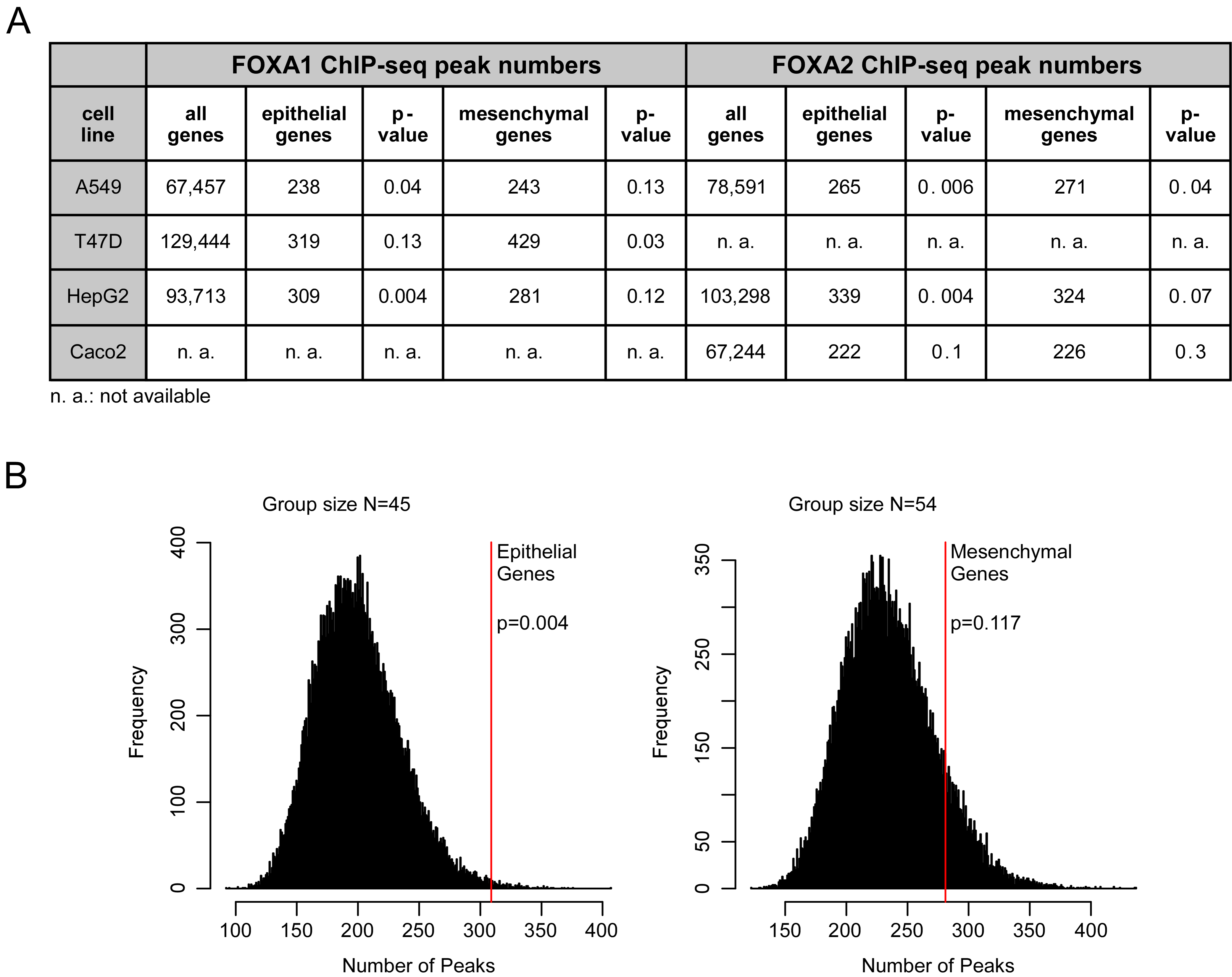

Supplement: S7 Fig — (A) Total numbers and genic distribution of FOXA1/FOXA2 ChIP-seq peaks in different cell lines. The p-values refer to the results of bootstrapping analyses (exemplary results for this analyses are shown in panel B) to test whether the number of FOXA1/FOXA2 ChIP-seq peaks at epithelial and mesenchymal genes is significantly different from random groups of genes. (B) FOXA1 ChIP-seq data from HepG2 cells were analyzed by a bootstrapping approach to estimate whether the number of binding regions at epithelial genes is significantly high or low. Out of all 22,000 annotated genes random groups of N = 45 or N = 54 genes representing the sample size of epithelial and mesenchymal gene groups, respectively, were selected, and the numbers of associated peaks were counted. The resulting distribution of associated peak numbers from 10,000 trials is shown. The red lines indicate the number of associated peaks for the epithelial and mesenchymal gene groups. The p-values shown were calculated from fitting a skewed normal distribution to the histogram. (TIF) [file pgen.1007109.s007.tif]

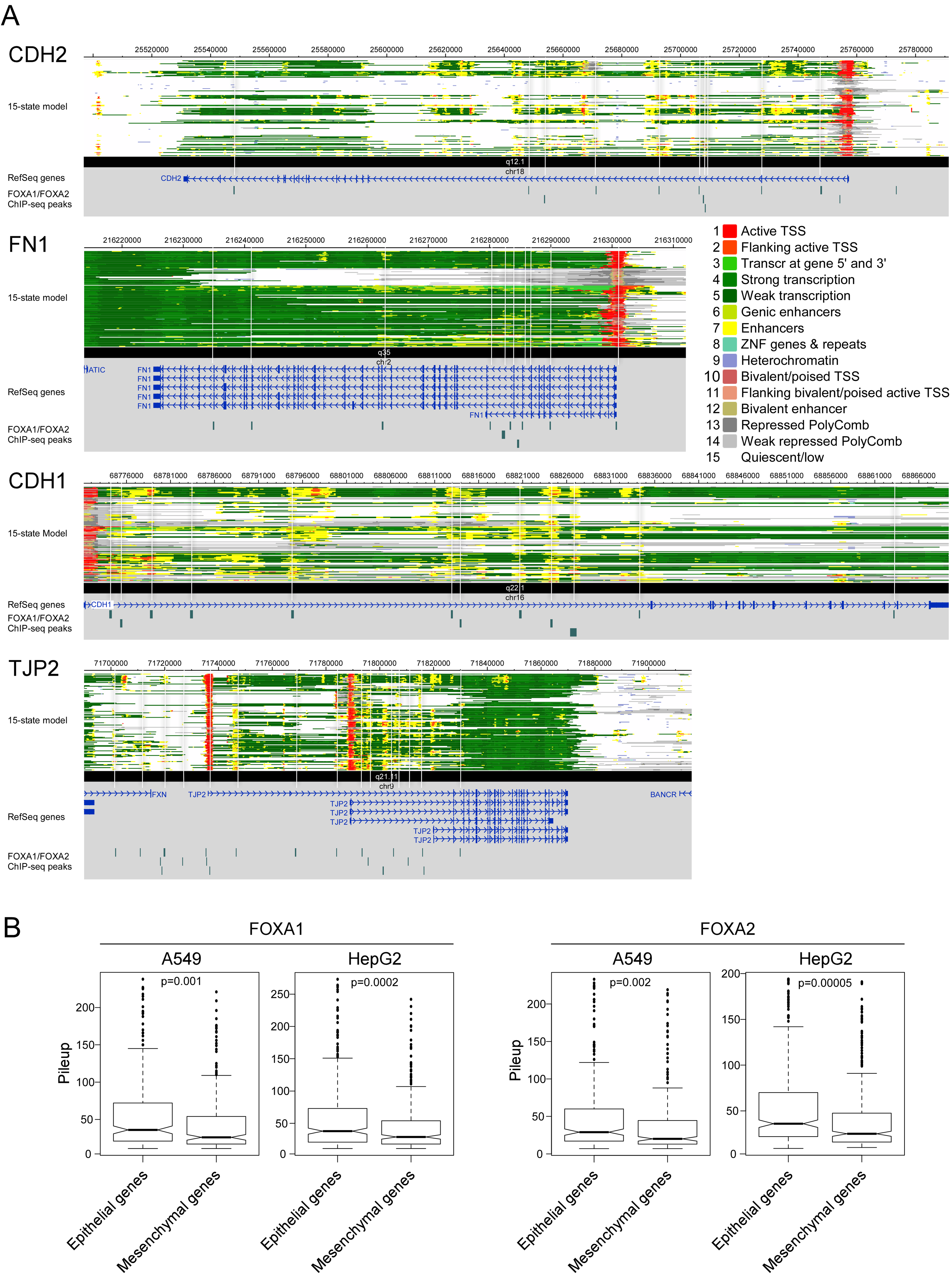

Supplement: S8 Fig — (A) Genome browser view of the 15-state chromatin model in relation to gene structure and FOXA1/FOXA2 binding regions for mesenchymal (CDH2, FN1) and epithelial (CDH1, TJP2) signature genes. Localization and width of FOXA1/FOXA2 ChIP-seq peaks are represented by the differently sized bars at the bottom of the scheme. Vertical white lines were added to facilitate aligning ChIP-seq peaks and chromatin states. (B) Box-whisker plots depicting the pileup values from the MACS output for the transcription factors FOXA1 and FOXA2 for the two cell lines A549 and HepG2. The boxplots indicate median values of the population and the boxes indicate the interquartile range (IQR) from the first to the third quantile of the pileup distribution. The notches indicate the +/-1.58 IQR/sqrt(n), where n denotes the number of respective peaks. The whiskers indicate the 1.5 IQR. sqrt: square root. (TIF) [file pgen.1007109.s008.tif]

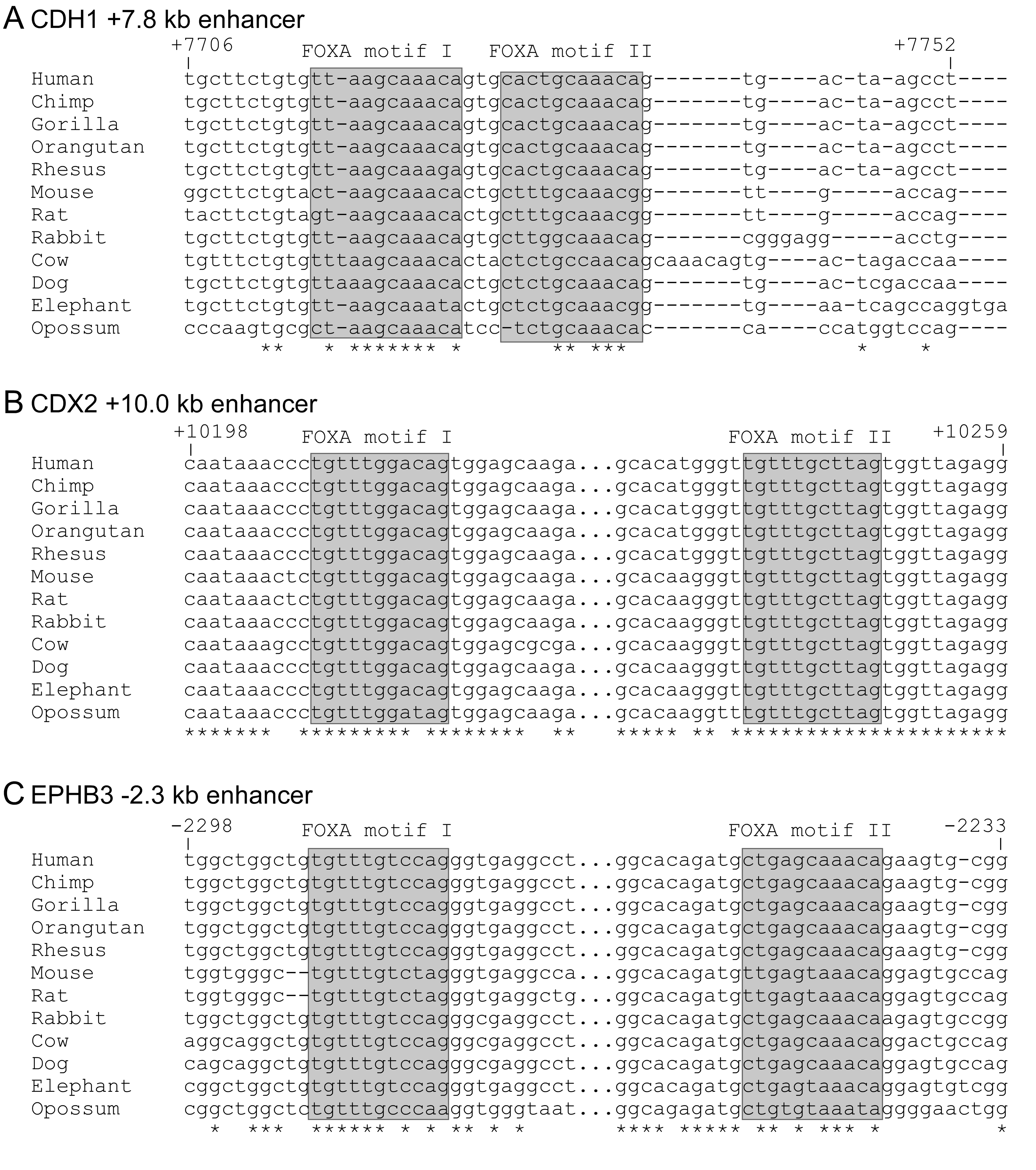

Supplement: S9 Fig — Alignment of the DNA sequences of the FOXA motifs in the CDH1 +7.8 kb (A), the CDX2 +10.0 kb (B) and the EPHB3 −2.3 kb (C) ECRs. The consensus of the FOXA motifs is highlighted by a grey box. Sequence identity is indicated by asterisks. The indicated base positions are relative to the transcriptional start site of the human DNA sequence based on the Ensembl genome browser. (TIF) [file pgen.1007109.s009.tif]

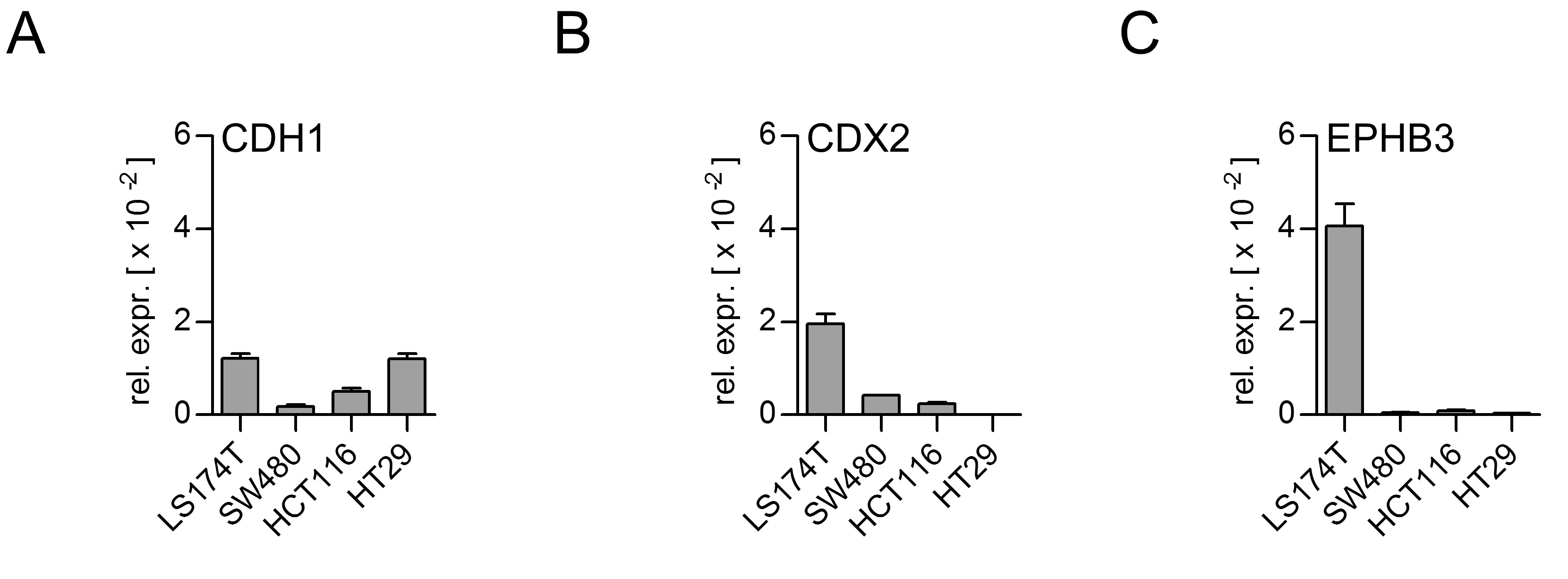

Supplement: S10 Fig — (A-C) qRT-PCR to analyze expression of CDH1 (A), CDX2 (B), and EPHB3 (C) in the indicated CRC cell lines. Data are shown as mean and SEM; n = 3. (TIF) [file pgen.1007109.s010.tif]

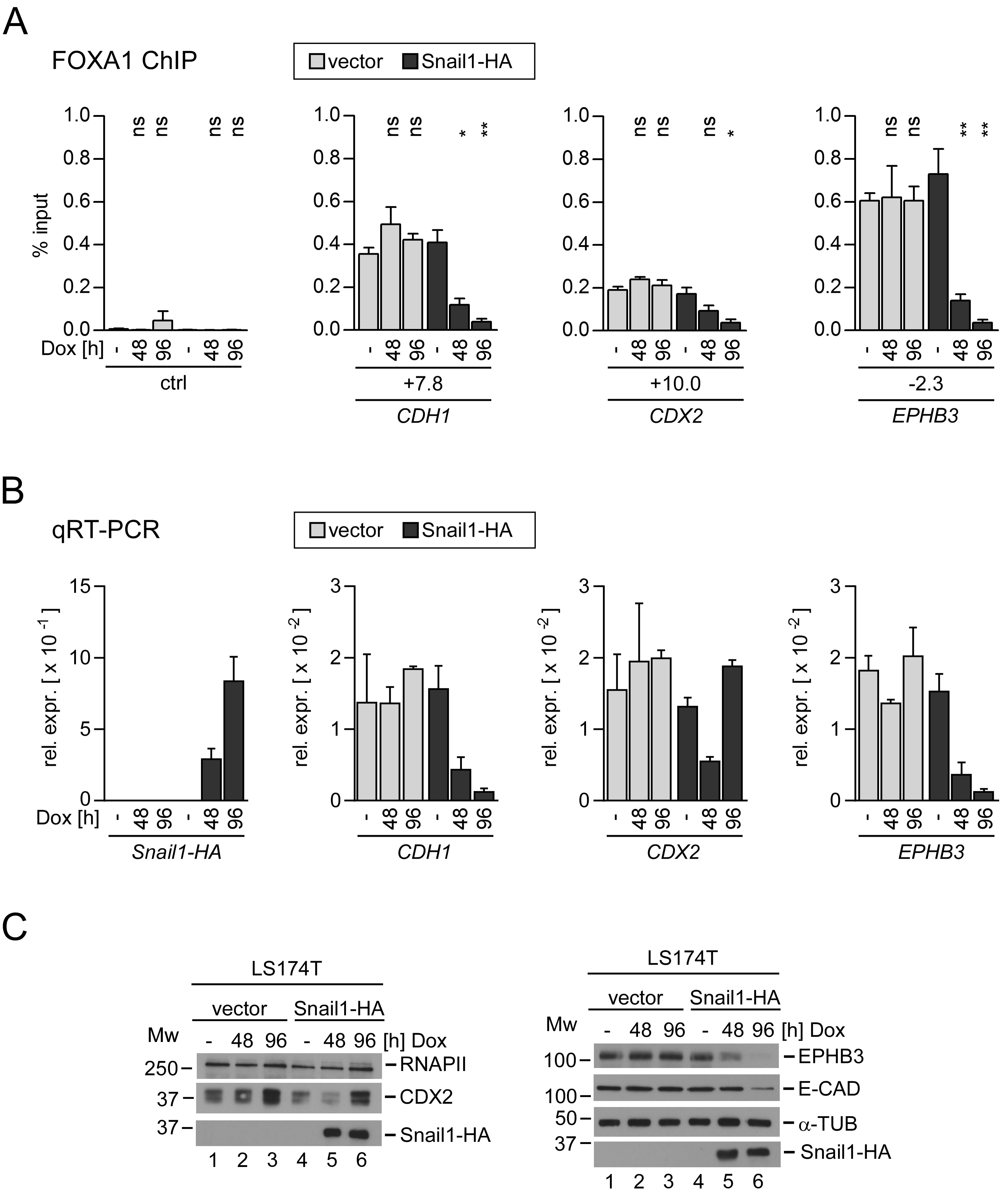

Supplement: S11 Fig — (A) ChIP analyses to test for binding of FOXA1 to the CDH1, the CDX2, and the EPHB3 enhancers in LS174T cells stably transduced with Dox-inducible retroviral control or Snail1-HA expression vectors. Data are given as percent input; n = 3. As control (ctrl) the EPHB3 locus at -10.0 kb was analyzed. (B) qRT-PCR to analyze expression of Snail1-HA, CDH1, CDX2, and EPHB3 in LS174T cells stably transduced with Dox-inducible retroviral control or Snail1-HA expression vectors. Data are shown as mean and SEM; n = 2. (C) Western Blot to analyze CDX2, E-CADHERIN (E-CAD), and EPHB3 protein levels in nuclear extracts (CDX2; left panel) and whole cell extracts (E-CADHERIN, EPHB3; right panel), respectively, upon Dox-induced Snail1-HA expression in LS174T cells. To monitor equal protein loading RNA polymerase II (RNAPII) and α-TUBULIN (α-TUB) was detected. MW = molecular weight in kDa. (TIF) [file pgen.1007109.s011.tif]

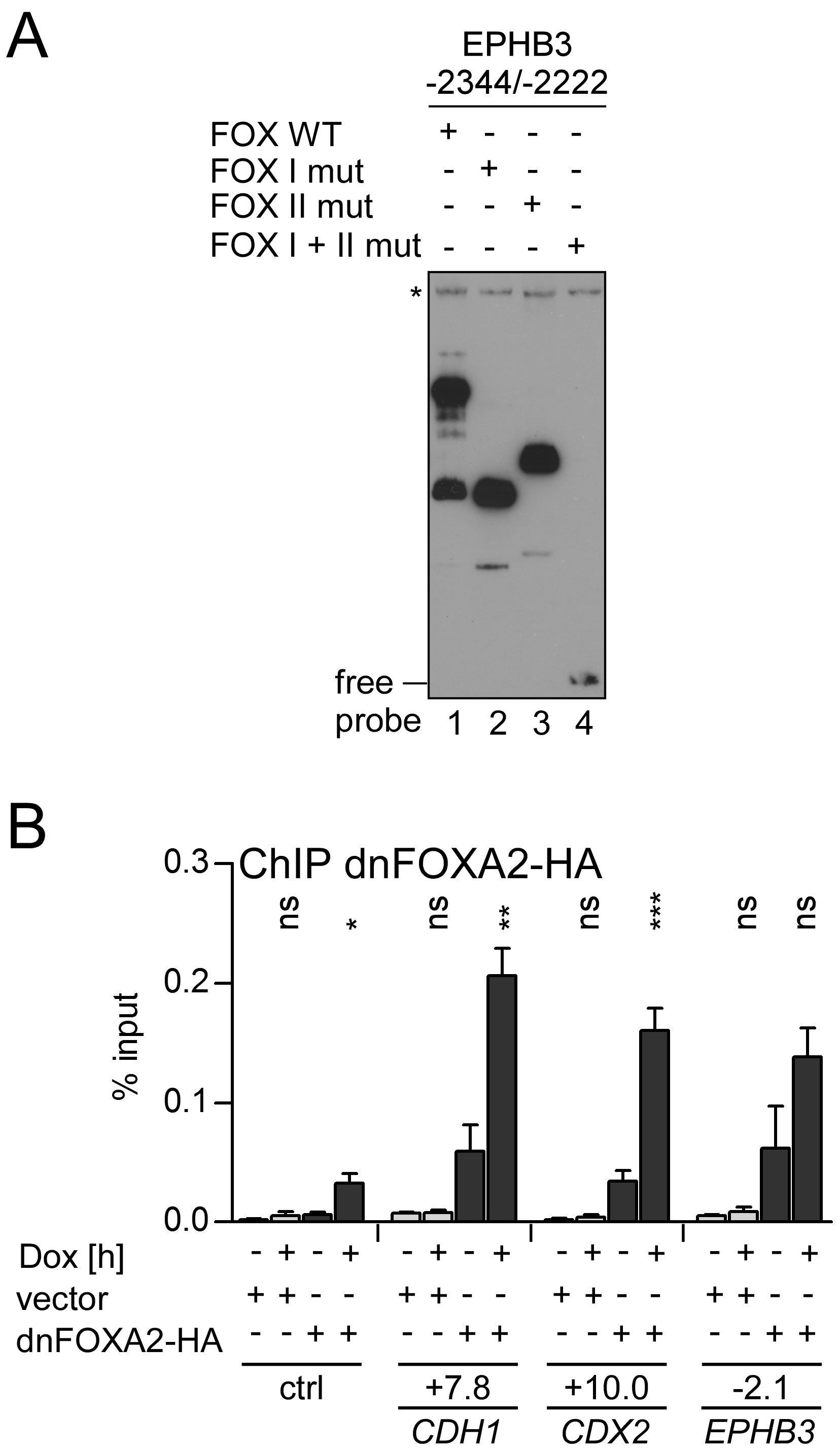

Supplement: S12 Fig — (A) EMSA to demonstrate binding of dnFOXA2-HA to the FOX binding motifs at the EPHB3 −2.3 kb enhancer. Asterisks: non-specific bands. mut: mutated. (B) ChIP analyses using anti-HA antibodies showing recruitment of dnFOXA2-HA to the CDH1 +7.8 kb, the CDX2 +10.0 kb and the EPHB3 −2.3 kb enhancers upon Dox treatment in LS174T cells stably transduced with Dox-inducible dnFOXA2-HA expression vectors. As control (ctrl) the EPHB3 locus at -10.0 kb was analyzed. Data are given as percent input. Shown is the mean and SEM; n≥4. (TIF) [file pgen.1007109.s012.tif]

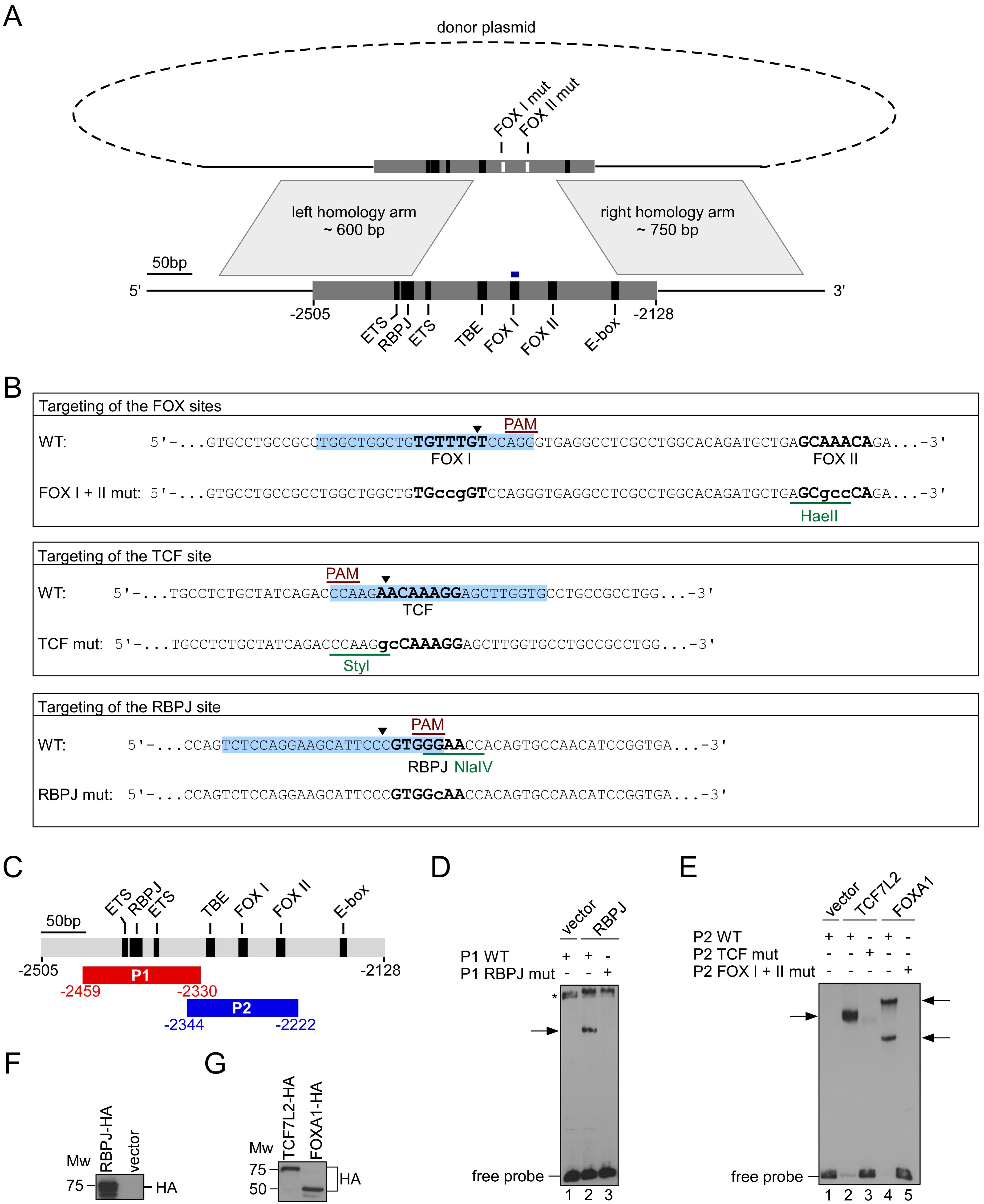

Supplement: S13 Fig — (A) Scheme of the strategy to mutate transcription factor binding sites at the EPHB3 enhancer exemplified by targeting the FOX binding motifs. The structure of the EPHB3 enhancer with the FOX binding sites is schematically shown at the bottom. The blue line denotes the position of the guide RNA. The structure of the donor plasmid is shown in the upper part. The left and right homology arms (approximately 600 and 750 bp, respectively) are schematically indicated by grey trapezes. (B) CRISPR/Cas9-mediated mutation of the FOX, TCF and RBPJ binding sites within the EPHB3 enhancer. A part of the DNA sequence of the EPHB3 enhancer with the FOX binding sites (upper), the TCF binding site (middle) and RBPJ binding site (lower) is given. The sequence of the transcription factor binding sites is written in bold. The sequence targeted by the guide RNA is highlighted by light blue shading. The PAM is indicated by the red line. The cutting site of the Cas9 nuclease is marked by the arrowhead. The mutations introduced in the transcription factor binding sites by integration of the donor plasmid are shown in lowercase. The recognition sequence of the restrictions enzymes (written in green) used to test for successful integration of the donor plasmid is indicated in the DNA sequence by the green line. (C) Scheme of the EPHB3 enhancer and its known transcription factor binding sites. The positions of the EMSA probes P1 and P2 are shown in red and blue, respectively. (D, E) EMSA showing that the mutations introduced into the genomic DNA of LS174T cells interfere with binding of RBPJ (D), the TCF factor TCF7L2 (E) and the FOXA protein FOXA1 (E). Arrows mark the protein::DNA complexes. (F, G) Western Blot analyses to control the expression of the in vitro translated proteins used for EMSA shown in (D, E). MW = molecular weight in kDa. (TIF) [file pgen.1007109.s013.tif]

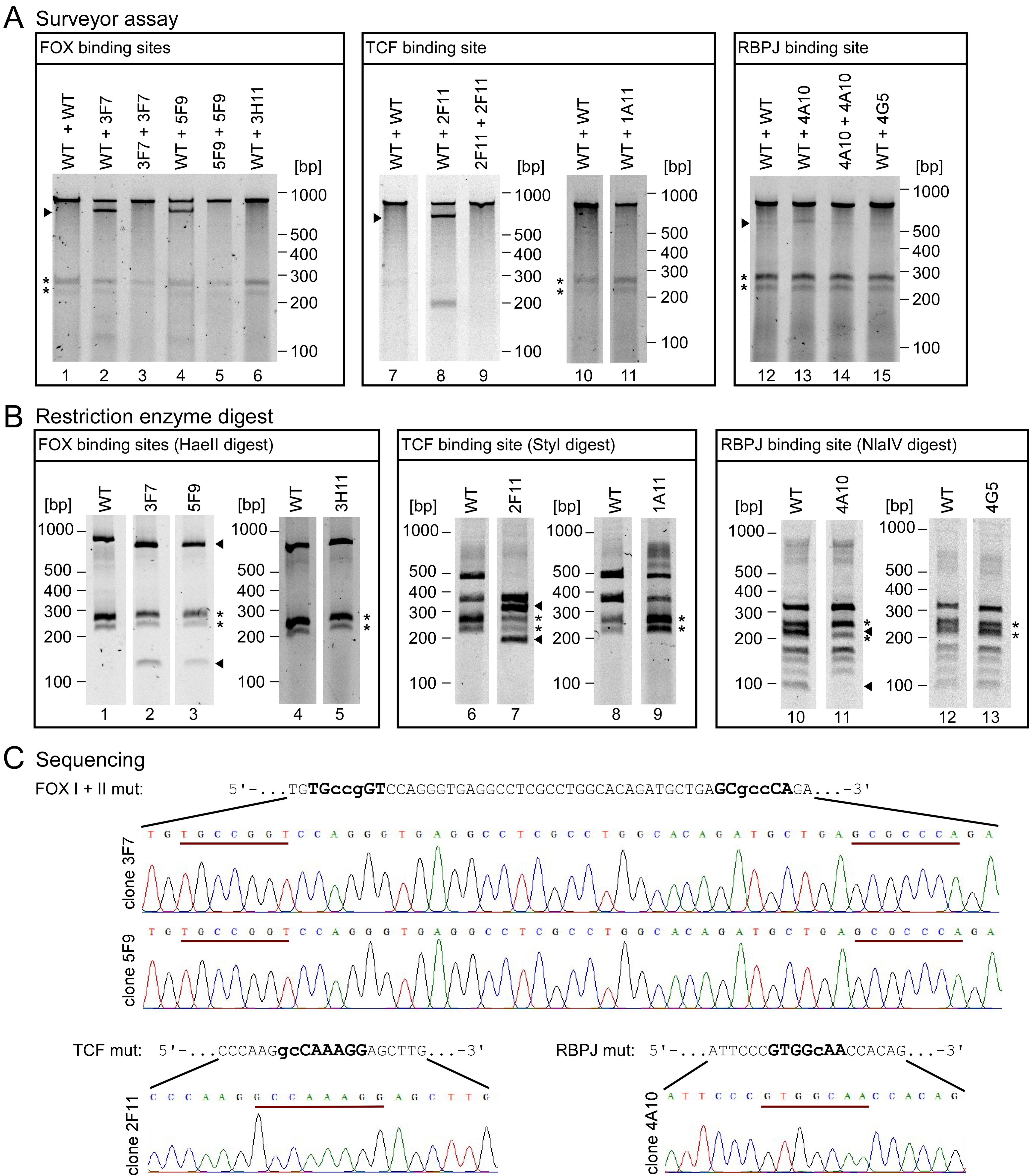

Supplement: S14 Fig — (A) Surveyor assay to test for genome alterations in LS174T cell clones subjected to CRISPR/Cas9 genome editing to mutate the FOX binding sites (left panel), the TCF binding site (middle panel), and the RBPJ binding site (right panel). Changes in the genomic DNA of cell clones were detected by cleavage of a mixture of PCR products from wild-type and cell clone DNA. The specific cleavage pattern is indicated by the arrowhead. To test for homozygous DNA alterations, PCR products of LS174T cell clones were subjected to Surveyor nuclease digest without admixture of wild-type PCR products. Asterisks: non-specific PCR products. (B) Restriction enzyme digest to test for successful integration of the donor plasmid and thus mutation of the FOX binding sites (left panel), the TCF binding site (middle panel) and the RBPJ binding site (right panel) in LS174T cell clones subjected to CRISPR/Cas9 genome editing. Changes in the cleavage pattern indicative of successful binding site mutations are shown by the arrowheads. Asterisks: non-specific PCR products. (C) Electropherograms of the sequencing analyses of PCR products from LS174T cell clones subjected to CRISPR/Cas9 genome editing showing mutations of the FOX binding sites (upper panel), the TCF binding site (lower left panel) and the RBPJ binding site (lower right panel) in the respective cell clones. The DNA sequences with the transcription factor motifs (written in bold) are given above the electropherograms. The bases that are changed compared to the wild-type sequence are written lowercase. The sequences of the mutated transcription factor binding sites are underlined in red in the electropherogram. (TIF) [file pgen.1007109.s014.tif]

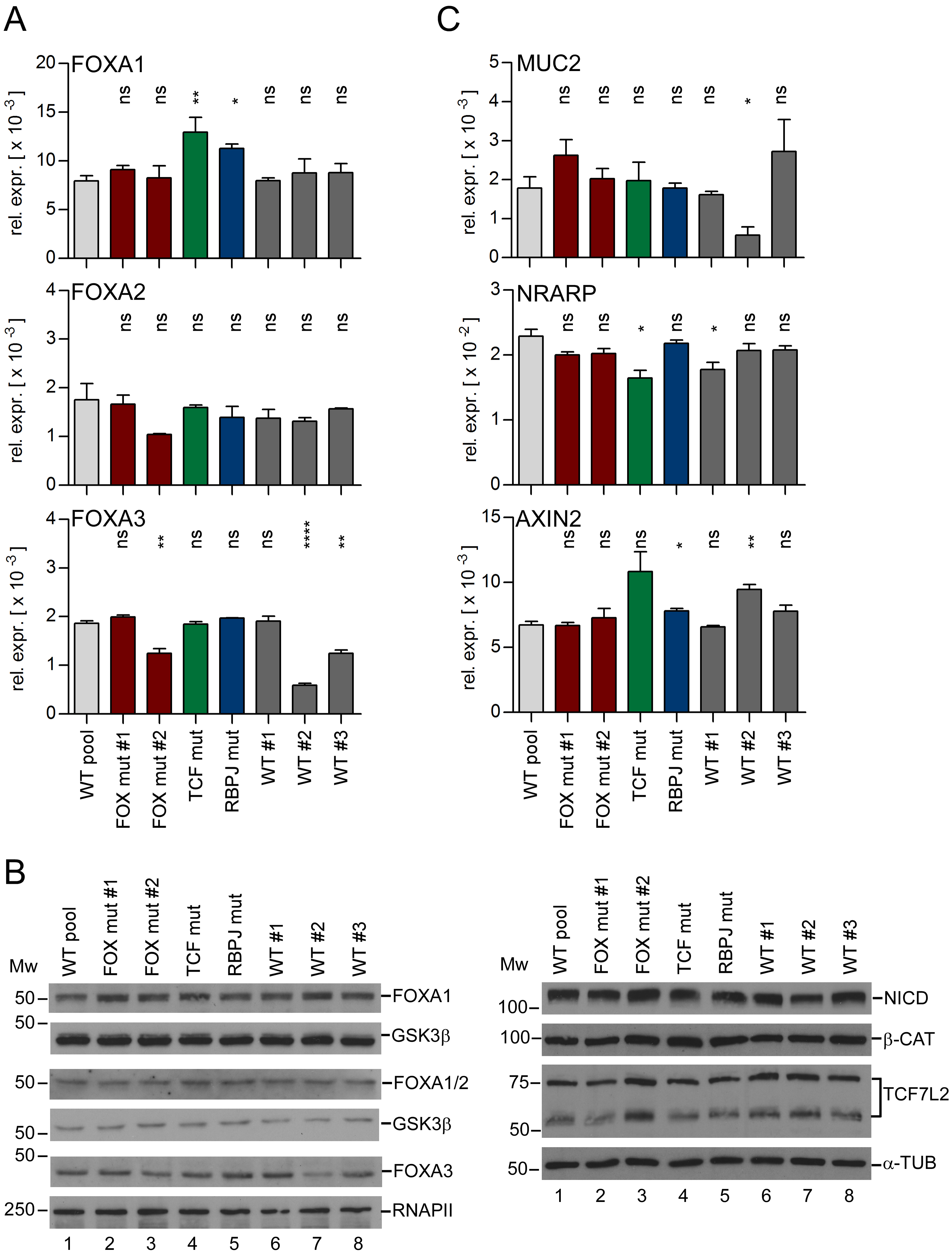

Supplement: S15 Fig — (A) qRT-PCR analyses to assess FOXA1, FOXA2, and FOXA3 relative expression (rel. expr.) levels in LS174T cell clones subjected to CRISPR/Cas9 genome editing. Data are shown as mean and SEM; n = 3. Statistical significance was calculated between the respective cell clone and the LS174T wild-type cell pool. (B) Western Blot to analyze abundance of the FOXA proteins FOXA1, FOXA1/2, and FOXA3 (left panel), NICD (indicative of active Notch signaling), and β-CATENIN (β-CAT) and TCF7L2 (mediators of Wnt/β-Catenin signaling) (right panel) in LS174T cell clones subjected to CRISPR/Cas9 genome editing. MW = molecular weight in kDa. α-TUBULIN (α-TUB), GSK3β and RNA polymerase II (RNAPII) immunodetections served as loading controls. (C) qRT-PCR analyses to test for expression levels of the FOXA1/2 target gene MUC2, the Notch target gene NRARP and the Wnt/β-Catenin target gene AXIN2 in LS174T cell clones subjected to CRISPR/Cas9 genome editing. Shown are the mean and SEM; n = 3. Statistical significance was calculated between the respective cell clone and the LS174T wild-type cell pool. (TIF) [file pgen.1007109.s015.tif]

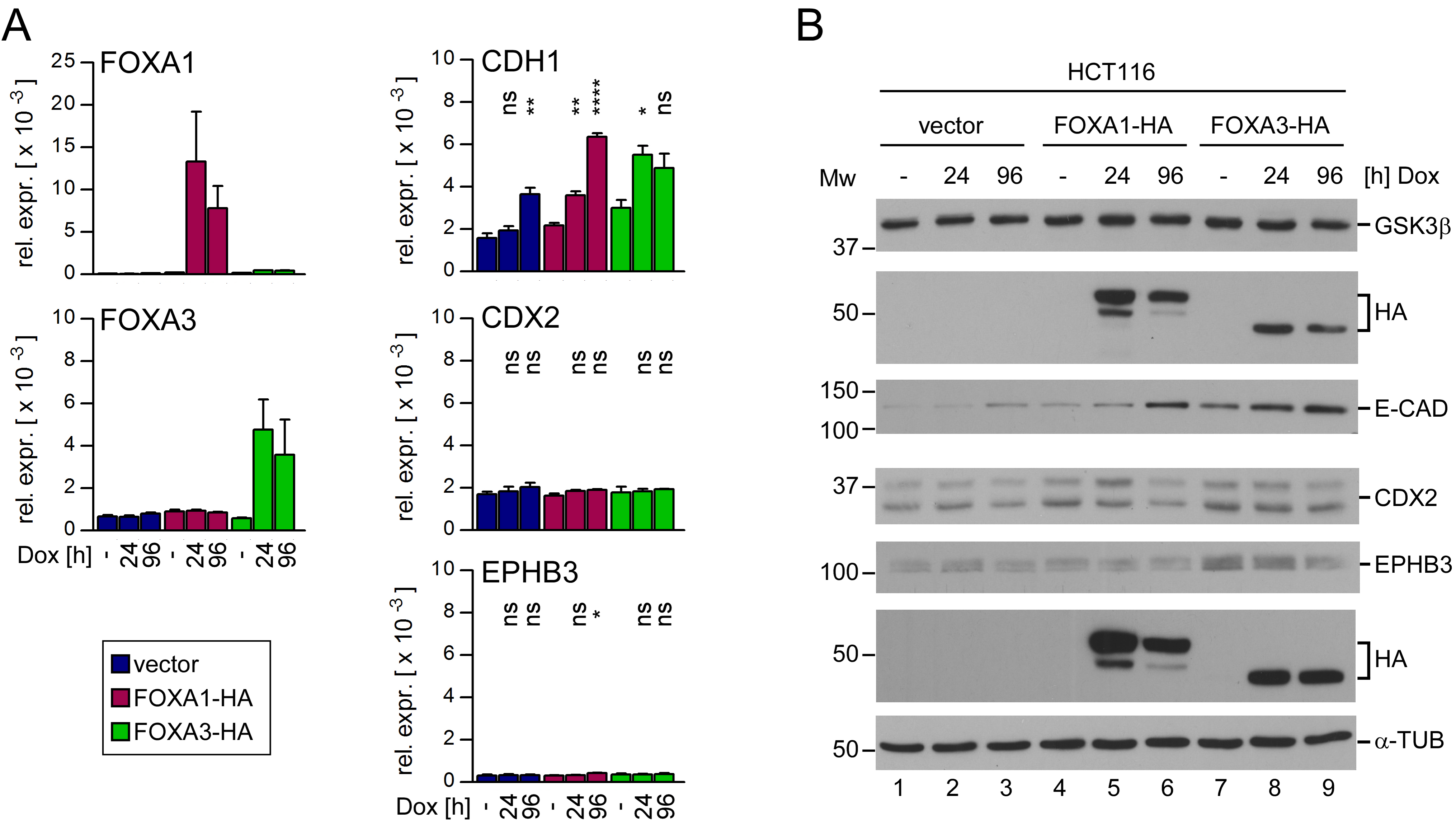

Supplement: S16 Fig — (A) qRT-PCR analyses to assess FOXA1, FOXA3, CDH1, CDX2, and EPHB3 relative expression (rel. expr.) levels in HCT116 cells stably transduced with Dox-inducible retroviral control, FOXA1-HA or FOXA3-HA expression vectors. Data are shown as mean and SEM; n = 3. (B) Western Blot to analyze FOXA1-HA, FOXA3-HA, E-CADHERIN, CDX2 and EPHB3 protein expression in HCT116 cells stably transduced with Dox-inducible retroviral control, FOXA1-HA or FOXA3-HA expression vectors. MW = molecular weight in kDa. GSK3β and α-TUBULIN (α-TUB) immunodetections served as loading controls. (TIF) [file pgen.1007109.s016.tif]
