# Supplementary material for: SNAIL1-mediated downregulation of FOXA proteins facilitates the inactivation of transcriptional enhancer elements at key epithelial genes in colorectal cancer cells
Source: PLoS Genet. 2017 Nov 20;13(11):e1007109. doi: 10.1371/journal.pgen.1007109 (PMC5714381; doi:10.1371/journal.pgen.1007109)
Supplement: S1 Table — (DOCX) [file pgen.1007109.s017.docx]

**S1 Table: Correlations between clinical parameters and expression levels of cluster 1 and cluster 2 genes**

| **Clinical parameter** | **Significance* of genes to be upregulated in association with clinical parameters** | |
| --- | --- | --- |
|  | Cluster 1 genes | Cluster 2 genes |
| Age (old vs. young) | 1.0 x 10^-15^ | 0.99 |
| Gender (female vs. male) | 0.6 | 0.02 |
| Disease-free survival (short vs. long) | 0.75 | 0.75 |
| Duke’s stage (D vs. A) | 1.8 x 10^-6^ | 0.99 |

* p-values are shown
