# Supplementary material for: SNAIL1-mediated downregulation of FOXA proteins facilitates the inactivation of transcriptional enhancer elements at key epithelial genes in colorectal cancer cells
Source: PLoS Genet. 2017 Nov 20;13(11):e1007109. doi: 10.1371/journal.pgen.1007109 (PMC5714381; doi:10.1371/journal.pgen.1007109)
Supplement: S2 Table — (DOCX) [file pgen.1007109.s018.docx]

# S2 Table: Compilation and nomenclature of genome-edited LS174T cell clones used in the study

| **Nomenclature in manuscript** | **LS174T cell clone** | **Description** | **Target region** |
| --- | --- | --- | --- |
| ***FOXA1* knockout** | | | |
| FOXA1^KO^A2A3^neg^ | 1C2 | *FOXA1* locus mutated;  FOXA1/FOXA2/FOXA3 triple-negative | FOXA1 exon 2 |
| FOXA1^WT^ | 1C6 | *FOXA1* locus wild-type |  |
| FOXA1^WT^ | 1E10 | *FOXA1* locus wild-type |  |
| FOXA1^KO^A2A3^neg^ | 2F8 | *FOXA1* locus mutated;  FOXA1/FOXA2/FOXA3 triple-negative |  |
| FOXA1^KO^A2A3^lo^ | 2F12 | *FOXA1* locus mutated;  FOXA1-negative; FOXA2/FOXA3 low |  |
| FOXA1^KO^ | 4F3 | *FOXA1* locus mutated;  FOXA1 negative; FOXA2/FOXA3 wild-type levels |  |
|  | | | |
| ***EPHB3* enhancer mutation** | | | |
| FOX mut #1 | 3F7 | Both FOX binding sites at the *EPHB3* enhancer are mutated on both alleles | FOX binding sites |
| FOX mut #2 | 5F9 | Both FOX binding sites at the *EPHB3* enhancer are mutated on both alleles |  |
| WT #1 | 3H11 | No changes in the DNA sequence at the *EPHB3* enhancer |  |
|  |  |  |  |
| TCF mut | 2F11 | The TCF binding site at the *EPHB3* enhancer is mutated on both alleles | TCF binding site |
| WT #2 | 1A11 | No changes in the DNA sequence at the *EPHB3* enhancer |  |
|  |  |  |  |
| RBPJ mut | 4A10 | The RBPJ binding site at the *EPHB3* enhancer is mutated on both alleles | RBPJ binding site |
| WT #3 | 4G5 | No changes in the DNA sequence at the *EPHB3* enhancer |  |
