# Supplementary material for: SNAIL1-mediated downregulation of FOXA proteins facilitates the inactivation of transcriptional enhancer elements at key epithelial genes in colorectal cancer cells
Source: PLoS Genet. 2017 Nov 20;13(11):e1007109. doi: 10.1371/journal.pgen.1007109 (PMC5714381; doi:10.1371/journal.pgen.1007109)
Supplement: S5 Table — (DOCX) [file pgen.1007109.s021.docx]

**S5 Table: List of EMT-associated epithelial and mesenchymal genes***

| **Epithelial genes** | | **Mesenchymal genes** | |
| --- | --- | --- | --- |
| CD24 | KRT18 | ACVR1 | MMRN2 |
| CD44 | KRT19 | ARMCX1 | MRAS |
| CDH1 | KRT8 | ASPN | MSN |
| CDX1 | LCN2 | AXL | NFIC |
| CDX2 | LYPD5 | CDH2 | RECK |
| CEACAM1 | MAL2 | CDON | RNF11 |
| CLDN4 | MECOM | CTGF | SFRP1 |
| CLDN7 | MET | DZIP1 | SH2D3C |
| CLDN9 | MLEC | ECM2 | SLC39A6 |
| CRB3 | MMP15 | EPHA3 | SMAD1 |
| DSC2 | MST1R | ETV5 | SMAD3 |
| ELF3 | PKP3 | FBLN1 | SNAI2 |
| EPHB3 | PRSS8 | FBLN5 | SPARC |
| ESRP1 | RSL1D1 | FGF1 | SRPX |
| ESRP2 | S100P | FGFR1 | STX2 |
| ETS2 | SDC1 | FLRT2 | TAGLN |
| FOXA2 | SFN | FN1 | TCF4 |
| FOXD2 | SHH | FOXC1 | TGFB1I1 |
| IHH | SOX9 | FOXC2 | TGFBR1 |
| ISX | SPINT1 | GLI2 | TIAM1 |
| ITGB4 | TMPRSS4 | GLI3 | TWIST1 |
| JUP | TNS4 | GLIS2 | TWIST2 |
| KAZALD1 |  | HTRA1 | VEGFB |
|  |  | LAMB2 | VIM |
|  |  | MAP3K3 | WASF3 |
|  |  | MEOX2 | WISP1 |
|  |  | MGP | ZFPM2 |

* EMT-associated genes extracted from Loboda et al. BMC Medical Genomics 4: 9. (2011)
