# Supplementary material for: SNAIL1-mediated downregulation of FOXA proteins facilitates the inactivation of transcriptional enhancer elements at key epithelial genes in colorectal cancer cells
Source: PLoS Genet. 2017 Nov 20;13(11):e1007109. doi: 10.1371/journal.pgen.1007109 (PMC5714381; doi:10.1371/journal.pgen.1007109)
Supplement: S6 Table — (DOCX) [file pgen.1007109.s022.docx]

**S6 Table: Sequences of oligonucleotides used in the study**

|  | **forward** **(5’ to 3’)** | **reverse** **(5’ to 3’)** |
| --- | --- | --- |
| **Primer for qRT-PCR** | | |
| AXIN2 | TGCTTTCGTGGAAATGACAG | AGGTGTGTGGAGGAAAGGTG |
| CDH1 | AAGAAGGAGGCGGAGAAGAG | GCCGCTTTCAGATTTTCATC |
| CDH11 | GTCCCTGAGCTCCCTAGAGT | TGAGAACGCCAGACACAGTT |
| CDX2 | CCCGAACAGGGACTTGTTTA | AGACCAACAACCCAAACAGC |
| CLDN3 | GGACTTCTACAACCCCGTGG | TGGTGGCCGTGTACTTCTTC |
| dnFOXA2 | CCTTCTACCGGCAGAACCAG | TTCTCGAACATGTTGCCCGA |
| EPHB3 | ACCACAGTCCATGCCATCACT | GTCCACCACCCTGTTGCTGTA |
| FOXA1 | AGCAGCAGCATAAGCTGGAC | GTGTTTAGGACGGGTCTGGA |
| FOXA2 | TGAGGCCCACCTGAAGCC | CTGGTAGTAGGAGGTATCTG |
| FOXA3 | ATTTCACTGGCCTGGAGCTC | GGCCCTGGTAGTAGACTCCA |
| GAPDH | ACCACAGTCCATGCCATCACT | GTCCACCACCCTGTTGCTGTA |
| LEf1 | CGAATGTCGTTGCTGAGTGT | GCAGACCAGCCTGGATAAAG |
| MUC2 | ACCCGCACTATGTCACCTTC | GGACAGGACACCTTGTCGTT |
| NRARP | CCCTTTTTAGCCTCCCAGAG | GGGCTGCATAGAAAATTGGA |
| SNAIL1 | GCTCCACAAGCACCAAGAGT | ATTCCATGGCAGTGAGAAGG |
| Snail1 | CTTGTGTCTGCACGACCTGT | CTTCACATCCGAGTGGGTTT |
| SNAIL2 | GAGCATTTGCAGACAGGTCA | GCTTCGGAGTGAAGAAATGC |
| **Primer for FAIRE and ChIP analyses** | | |
| AXIN2 (-11138/-10942) ^a^ | TTGACCTCGGGAATCTGTTC | CCATCCCCACCTTCTCTTCT |
| CDH1 (+7699/+7852) | CCCTGCCTGCTTCTGTGTTA | GGTCCCCTTTCCTAAGCCAC |
| CDH1 (+95995/+96342) | AGACTTCTTGCCCCAGATGA | AACCACCAGCAACGTGATTT |
| CDX2 (-4030/-3882) | AAACGGAGTGTCACCCTGTC | ATTAGCTGGGAGTGGAAGCG |
| CDX2 (+10222/+10377) | AGCAAGAGCACATGGGTTGT | CATCTCAGAGAGCAGCCGAG |
| EPHB3 (-11032/-10875) | TCTTTCACCTGGAGGTGGTT | CAATGTTGCCAGTTGGATTG |
| EPHB3 (-7059/-6919) ^a^ | TTCTTCGCAGTTGTCCAGTG | GGCCTTAGAGAGGGATTTGG |
| EPHB3 (-2390/-2223) | CAACATCCGGTGAGGACAG | ATCTGTGAAGCCGCACTTCT |
| EPHB3 (-2247/-2100) | GCTTCACAGATCCGCCTTAC | CCTAAGGCTCAGAGATGTGTCC |
| EPHB3 (-237/-144) ^a^ | tccgcccaatctaattcatc | agttcagttggcggtgtctc |
| EPHB3 (-4/+141) | GGGACAGCAGAAGTGAGTACG | GTGCAGGTGGTCGTGATT |
| FOXA1 (-986/-723) | CTCGGCTGACTCAGATGACC | TCCTGCTCTTTGCTTCCTCC |
| FOXA1 (-480/-298) | CCTCCGGGCCTAAACCAATT | CACAAAGACGCTCGCACCTA |
| **Primer for Surveyor assay** | | |
| EPHB3 (-2958/-2144) | TTATCAGGCTCCTGCAGTGC | CTGGGGTTGGACACAGAGAT |
| **Primer for generation of biotinylated EMSA probes** | | |
| CDH1 (+7659/+7770) | CTCCCTCTCACCCAGCAAAC | TCTTCCTAACAAGGCACTAGGC |
| CDX2 (+10172/+10283) | GCTATGCGTTGCCGTGAAAC | AGCGGGACAATGTCGACTTT |
| EPHB3 (-2459/-2330) | CTTGCCCTGGGCTGTTATAG | TCTGATAGCAGAGGCAGCAG |
| EPHB3 (-2344/-2222) | CCTCTGCTATCAGACCCAAGA | ATCTGTGAAGCCGCACTTCT |
| **Primer for site-directed mutagenesis (oligonucleotides (5’-3’), mutated bases are underlined)** | | |
| CDH1 FOX I mut | GCTTCTGTGTTAAGCGGGCAGTGCACTGCAAAC | |
| CDH1 FOX II mut | CAGTGCACTGCCGGCAGTGACTAAGCCTAGTGCC | |
| CDX2 FOX I mut | CTGTCAATAAACCCTGTCCGGACAGTGGAGCAAGAG | |
| CDX2 FOX II mut | GCAAGAGCACATGGGTTGCCGGCTTAGTGGTTAGAGGT | |
| EPHB3 FOX I mut | CGCCTGGCTGGCTGTGCCGGTCCAGGGTGAGGC | |
| EPHB3 FOX II mut | CTGGCACAGATGCTGAGCGCCCAGAAGTGCGGCTTCAC | |
| EPHB3 RBPJ mut | GAAGCATTCCCGTGGCAACCACAGTGCCAAC | |
| EPHB3 TCF mut | TGCTATCAGACCCAAGGCCAAAGGAGCTTGGTGC | |
| FOXA1 Ebox I mut | AAACCGGTGCTGTGACTAAGCTTCTCTTAGCCGCAGGTAC | |
| FOXA1 Ebox II mut | GGCGGCCGCCTTGAAGCTTCACCTCGGGCTTTGT | |

^a^ primer pairs used only for FAIRE experiments
