# Supplementary material for: SNAIL1-mediated downregulation of FOXA proteins facilitates the inactivation of transcriptional enhancer elements at key epithelial genes in colorectal cancer cells
Source: PLoS Genet. 2017 Nov 20;13(11):e1007109. doi: 10.1371/journal.pgen.1007109 (PMC5714381; doi:10.1371/journal.pgen.1007109)
Supplement: S7 Table — (DOCX) [file pgen.1007109.s023.docx]

# S7 Table: Antibodies used for immunoblotting, immunofluorescence, and ChIP

| **Primary antibodies used for immunoblotting (IB) and immunofluorescence (IF)** | | | |
| --- | --- | --- | --- |
| **Antigen** | **Species** | **Dilution** | **Supplier** |
| β-CATENIN | mouse | 1:1000 | 610154; BD Biosciences, Franklin Lakes, USA |
| CADHERIN11 | mouse | 1:500 (IB)  1:400 (IF) | #13577; Cell Signaling Technology, Danvers, USA |
| CDX2 | rabbit | 1:1000 | #12306; Cell Signaling Technology, Danvers, USA |
| CLAUDIN3 | rabbit | 1:1000 (IB)  1:200 (IF) | #83609; Cell Signaling Technology, Danvers, USA |
| E-CADHERIN | mouse | 1:1000 (IB)  1:200 (IF) | 610404; BD Biosciences, Franklin Lakes, USA  610182; BD Biosciences, Franklin Lakes, USA |
| EPHB3 | mouse | 1:1000 | H00002049-M01; Abnova, Taipei City, Taiwan |
| FOXA1 | goat | 1:1000 | ab5089; abcam, Cambridge, UK |
| FOXA2 | goat | 1:1000 | sc-6554X; Santa Cruz, Heidelberg, Germany |
| FOXA3 | goat | 1:1000 | sc-5361X; Santa Cruz, Heidelberg, Germany |
| GSK3β | mouse | 1:1000 | 610201; BD Biosciences, Franklin Lakes, USA |
| HA epitope tag | rat | 1:1000 | 3F10; Roche Applied Science, Mannheim, Germany |
| LEF1 | rabbit | 1:1000 (IB)  1:400 (IF) | #2330; Cell Signaling Technology, Danvers, USA |
| cleaved NOTCH1 (NICD) | rabbit | 1:1000 | #4147; Cell Signaling Technology, Danvers, USA |
| RNA Pol II | rabbit | 1:1000 | sc-899; Santa Cruz, Heidelberg, Germany |
| SNAIL1 | rabbit | 1:1000 | #3879; Cell Signaling Technology, Danvers, USA |
| SNAIL2 | rabbit | 1:1000 (IB)  1:400 (IF) | #9585; Cell Signaling Technology, Danvers, USA |
| TCF7L2 | rabbit | 1:1000 | #2565; Cell Signaling Technology, Danvers, USA |
| α-TUBULIN | mouse | 1:10000 | T9026; Sigma-Aldrich, Munich, Germany |
| **Primary antibodies used for ChIP analyses** | | | |
| **Antigen** | **Species** | **Amount/ChIP** | **Supplier** |
| FOXA1 | goat | 3 µg | ab5089; abcam, Cambridge, UK |
| FOXA3 | goat | 2 µg | sc-5361X; Santa Cruz, Heidelberg, Germany |
| H3 | rabbit | 2 µg | ab1791; abcam, Cambridge, UK |
| H3K27ac | rabbit | 2 µg | ab4729; abcam, Cambridge, UK |
| H3K4me1 | rabbit | 2 µg | pAb-037-050; Diagenode, Liège, Belgium |
| HA | rabbit | 1 µg | ab9110; abcam, Cambridge, UK |
